# A chromosome-level genome assembly and annotation of the desert horned lizard, *Phrynosoma platyrhinos*, provides insight into chromosomal rearrangements among reptiles

**DOI:** 10.1093/gigascience/giab098

**Published:** 2022-02-04

**Authors:** Nazila Koochekian, Alfredo Ascanio, Keaka Farleigh, Daren C Card, Drew R Schield, Todd A Castoe, Tereza Jezkova

**Affiliations:** Department of Biology, Miami University, Oxford, OH 45056, USA; Department of Biology, Miami University, Oxford, OH 45056, USA; Department of Biology, Miami University, Oxford, OH 45056, USA; Department of Organismic & Evolutionary Biology, Harvard University, Cambridge, MA 02138, USA; Museum of Comparative Zoology, Harvard University, Cambridge, MA 02138, USA; Department of Ecology and Evolutionary Biology, University of Colorado, Boulder, CO 80309, USA; Department of Biology, University of Texas at Arlington, Arlington, TX 76019, USA; Department of Biology, Miami University, Oxford, OH 45056, USA

**Keywords:** microchromosome, macrochromosome, gene content, synteny, Reptilia

## Abstract

**Background:**

The increasing number of chromosome-level genome assemblies has advanced our knowledge and understanding of macroevolutionary processes. Here, we introduce the genome of the desert horned lizard, *Phrynosoma platyrhinos*, an iguanid lizard occupying extreme desert conditions of the American southwest. We conduct analysis of the chromosomal structure and composition of this species and compare these features across genomes of 12 other reptiles (5 species of lizards, 3 snakes, 3 turtles, and 1 bird).

**Findings:**

The desert horned lizard genome was sequenced using Illumina paired-end reads and assembled and scaffolded using Dovetail Genomics Hi-C and Chicago long-range contact data. The resulting genome assembly has a total length of 1,901.85 Mb, scaffold N50 length of 273.213 Mb, and includes 5,294 scaffolds. The chromosome-level assembly is composed of 6 macrochromosomes and 11 microchromosomes. A total of 20,764 genes were annotated in the assembly. GC content and gene density are higher for microchromosomes than macrochromosomes, while repeat element distributions show the opposite trend. Pathway analyses provide preliminary evidence that microchromosome and macrochromosome gene content are functionally distinct. Synteny analysis indicates that large microchromosome blocks are conserved among closely related species, whereas macrochromosomes show evidence of frequent fusion and fission events among reptiles, even between closely related species.

**Conclusions:**

Our results demonstrate dynamic karyotypic evolution across Reptilia, with frequent inferred splits, fusions, and rearrangements that have resulted in shuffling of chromosomal blocks between macrochromosomes and microchromosomes. Our analyses also provide new evidence for distinct gene content and chromosomal structure between microchromosomes and macrochromosomes within reptiles.

## Background

**Figure 1:**
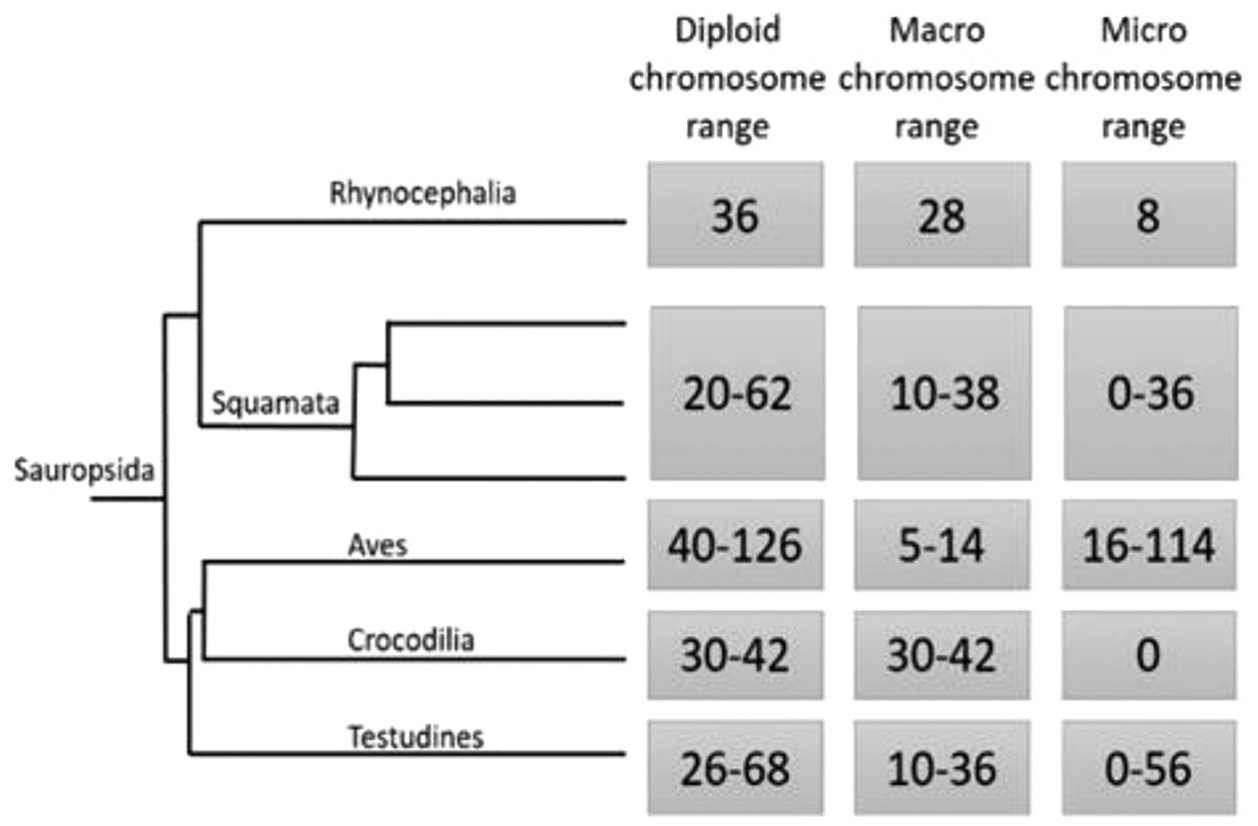
For each major clade, we list diploid chromosome numbers, macrochromosome numbers, and microchromosome numbers based on previous research [1]. The phylogeny was adapted from [2].

The increasing number of available chromosome-level genome assemblies of non-traditional model organisms has advanced our understanding of genome evolution over large time scales, including intra- and inter-chromosomal rearrangements and karyotype evolution across amniote vertebrates. A major gap in our understanding of amniote genome structure, composition, and evolution has been due to the lack of representative reptilian genomes of high enough quality to compare chromosome composition and structure. From data that are available, reptiles (the clade of Sauropsida) seem to exhibit particularly high levels of karyotypic variation (Fig. 1) [1, 2]. Much of this karyotypic variation seems to be due to frequent merging, splitting, and rearrangements among chromosomes, resulting in varying numbers and sizes of chromosomes even among closely related taxa (Fig. 1). Unlike mammalian genomes, which lack microchromosomes, most reptilian genomes contain both macrochromosomes and microchromosomes [3]. The condition of possessing both macro- and microchromosomes seems to represent an ancient ancestral state that spans 400–450 million years of evolutionary history because microchromosomes are present in many ancient chordates, fish, and amphibians and all amniote vertebrates except mammals and crocodilians [3]. Microchromosomes are generally identified by their smaller size (50-Mb threshold in squamates [4]). In the chicken, for example, microchromosomes range from 3.5 to 23 Mb [5], compared to macrochromosomes, which range from 40 to 250 Mb [6].

Although microchromosome organization in avian species is relatively conserved at a karyotypic level [7], microchromosomes of non-avian reptiles vary considerably in number and size [8, 9], potentially owing to relatively high recombination rates [10] that lead to higher rates of chromosomal rearrangement [3, 11]. Despite being a promising system in which to study karyotypic evolution, relatively little is known about the genomic features of macrochromosomes and microchromosomes and how these features evolve across Reptilia [12]. Moreover, microchromosomes seem structurally and functionally distinct from macrochromosomes [13], and a deeper characterization of these distinctions may improve our understanding of the functional and evolutionary significance of the presence/absence of microchromosomes, and the presence of genes on micro- versus macrochromosomes. Despite interest in the processes and patterns related to chromosome evolution in reptiles, progress has been limited by the availability of relatively few high-quality reptile genomes available for comparative study. In lizards, only 5 genomes are annotated and assembled at the level of chromosomes (i.e., chromosome-size scaffolds that in many cases have been ascribed to specific chromosomes): the green anole, *Anolis carolinensis*, with 6 chromosomes and 7 microchromosomal linkage groups [14]; the viviparous lizard, *Zootoca vivipara*, with 19 chromosomal linkage groups [15]; the sand lizard, *Lacerta agilis*, with 18 autosomes and Z and W sex chromosomes [16]; the common wall lizard, *Podarcis muralis*, with 18 autosomes and a Z sex chromosome [17]; and the Argentine black and white tegu, *Salvator merianae*, with chromosome-scale scaffolds that have not been fully ascribed to specific chromosomes [18].

Here we present a new chromosome-level genome assembly of the desert horned lizard (*Phrynosoma platyrhinos*; NCBI:txid52577) and use this genome to conduct comparative analysis of chromosome content and evolution across reptiles. This species is widely distributed across the southwestern deserts of north America, including some of the hottest and driest places on Earth (e.g., Death valley in the Mojave Desert [19]), which makes it an attractive model organism to study adaptation to extreme thermal environments. We have annotated the genome assembly and assessed large-scale structure and composition of the genome across macrochromosomes and microchromosomes. Using this new resource, we conduct synteny analyses to explore major changes in genome organization by making comparisons with existing chromosome-level annotated genomes of other lizards (*A. carolinensis, S. merianae, L. agilis, Z. vivipara*, and *P. muralis*), snakes (*Crotalus viridis* [20], *Thamnophis elegans* [21], and *Naja naja* [22]), 1 bird (*Gallus gallus* [23]), and turtles (*Trachemys scripta* [24], *Gopherus evgoodei* [25], and *Dermochelys coriacea* [9]). Our findings reveal differences in structure and gene content of macrochromosomes and microchromosomes in *P. platyrhinos* and highlight numerous chromosomal rearrangements among reptiles.

## Analysis

### Genome assembly, transcriptome assembly, and chromosome identification

The genome of *P. platyrhinos* was sequenced at 21,053.74-fold physical coverage using the Dovetail Genomics HiRise™ [26] sequencing and assembly approach that combines a contig-level assembly produced from shotgun Illumina sequencing with long-range scaffolding data from Chicago and Hi-C library preparations (Table 1). The final assembly included 5,294 total scaffolds, with 7 large scaffolds and 10 smaller scaffolds comprising 99.56% of the genome assembly. The known karyotype of the species is composed of 6 macrochromosomes and 11 microchromosomes [27, 28], and we assumed this karyotype when linking chromosomes to their representative assembly scaffolds. Using chromosome-linked gene markers from *A. carolinensis* and *Leiolepis reevesii* [29], the 7 largest scaffolds were assigned to macrochromosomes 1–6 (2 scaffolds corresponded to the 2 arms of macrochromosome 3; Supplementary Tables S1 and S2). Ten smaller scaffolds were assigned to microchromosomes, and 1 of these scaffolds was manually split into 2 microchromosomes (Supplementary Table S1). We followed previous studies [8] to infer the location of the putative split between chromosomes by combining evidence from physically linked Chicago scaffolds that cannot span multiple chromosomes, repeat element and GC composition, and synteny with chromosomes of other species (see Methods).

**Table 1:** Basic information about the *P. platyrhinos* genome assembly

Assembly	Chicago assembly	Chicago + Hi-C assembly
Longest scaffold (bp)	361,415,485	396,190,715
No. of scaffolds	5,458	5,294
No. of scaffolds >1 kb	5,458	5,294
Contig N50 (kb)	12.04	12.04
Scaffold N50 (kb)	63,431	273,213
No. of gaps	258,150	258,317
Percent of genome in gaps	1.54%	1.54%

The chromosome-linked gene markers used to identify chromosome scaffolds do not identify specific microchromosome numbers (Supplementary Table S2), so we ordered the assembled *P. platyrhinos* microchromosomes by descending length and numbered them microchromosomes 1–11 (Supplementary Table S1). Sex chromosomes are conserved across iguanid lizards [30], and we identified microchromosome 9 as the X chromosome in *P. platyrhinos* on the basis of homology with X-linked markers in *A. carolinensis* (*ATP2A2*, *FZD10*, and *TMEM132D* [30]; Supplementary Table S2).

RNA-sequencing of 8 tissues (liver, lungs, brain, muscle, testes, heart, eyes, and kidneys) was used to assemble the transcriptome of *P. platyrhinos* using Trinity r2014 0413p1 [31]. The final transcriptome assembly contained 199,541 transcripts comprising 199,500 Trinity-annotated genes, with an average length of 1,438 bp and an N50 length of 2,420 bp.

### Genome annotation and chromosomal composition

We annotated 20,764 protein-coding genes in the *P. platyrhinos* genome assembly (JAIPUX010000000) using the gene prediction software MAKER v. 2.31.10 [32] and gene predictions based on AUGUSTUS v. 3.2.3. [33]. Among the total annotated genes, 16,384 genes were identified using searches against protein sequences in databases NCBI and Interpro [34]. We identified 4,324 complete and fragmented BUSCO markers in the *P. platyrhinos* genome annotation from the total 5,310 BUSCO markers present in the library “tetrapoda_odb10.2019–11-20” (Table 2). Our repeat annotation identified 44.45% of the genome as repetitive elements (Supplementary Table S3) using RepeatModeler v. 1.0.11 [35] and RepeatMasker v. 4.0.8 [36]. The major components of the genomic repeat content included simple sequence repeats (6.90%), as well as L2/CR1/Rex (6.88%), hobo-Activator (5.98%), and Tourist/Harbinger (4.90%) transposable element families (Supplementary Table S3).

**Table 2:** BUSCO summary results

BUSCO benchmark	No. (%)
Present BUSCOs	4,324 (81.5)
Complete BUSCOs	3,640 (68.6)
Complete single-copy BUSCOs	3,609 (68.0)
Complete duplicated BUSCOs	31 (0.6)
Fragmented BUSCOs	684 (12.9)
Missing BUSCOs	986 (18.5)
Total BUSCO groups searched	5,310 (100)

**Figure 2:**
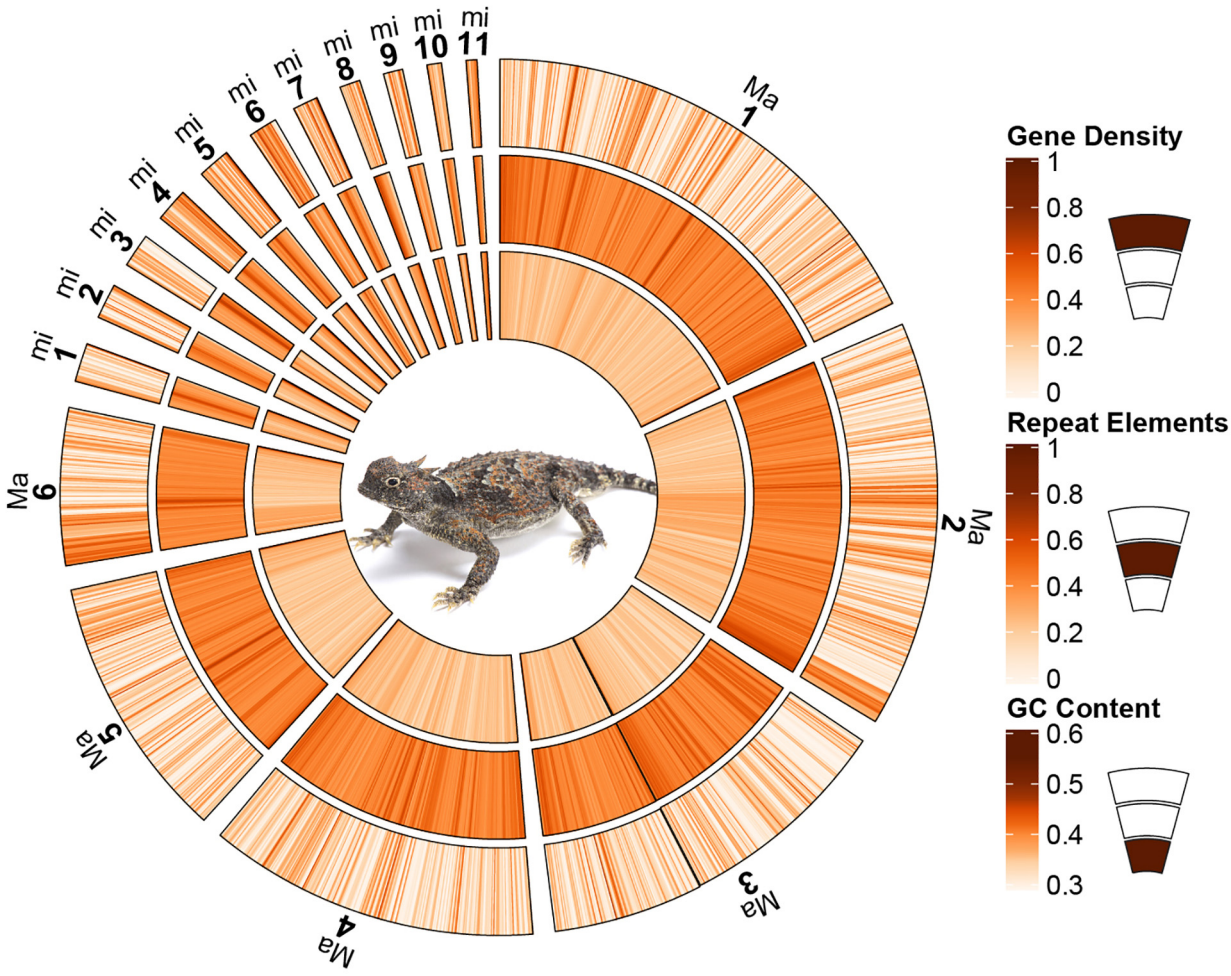
The genome content of P. *platyrhinos*. The outer circle shows gene density on each chromosome, the middle circle shows repeat element density, and the inner one shows GC content. Each estimate is calculated per 1 million base pair window in each chromosome. “Ma” indicates macrochromosomes, and “mi,” microchromosomes. Two scaffolds for macrochromosome 3 are attached together (the black line) and 2 microchromosomes (mi6 and mi10) resulting from a single scaffold are showed separately and in size order with the rest of the microchromosomes.

Chromosomal composition analyses indicate that overall gene density (GD) and GC content tended to be lower on *P. platyrhinos* macrochromosomes (mean GD = 0.19 [SD 0.14], median = 0.17 per Mb; mean GC% = 35.9% [SD 1.2], median = 35.9%) than microchromosomes (mean GD = 0.27 [SD 0.16], median = 0.29 per Mb; mean GC% = 38.5% [SD 2.8], median = 38.2%; Fig. 2 and Supplementary Fig. S1). Conversely, repeat element density tended to be higher on macrochromosomes (mean 44.6% [SD 5.6], median = 43.3% per Mb) than microchromosomes (mean 39.4% [SD 10], median = 38.1% per Mb; Fig. 2 and Supplementary Fig. S1). These differences in GD, GC content, and repeat elements between macro- and microchromosomes were statistically significant (Wilcoxon-W = 137,011, *P*-value = 5.7 * 10^–16^ for GD; Wilcoxon-W = 68,322, *P*-value < 2.2 * 10^–16^ for GC-content; and Wilcoxon-W = 283,330, *P*-value < 2.2 * 10^–16^ for repeat elements).

### Pathway analysis

We assessed whether macrochromosomes and microchromosomes contain distinct functional classes of genes using pathway analyses. From the total of 16,384 protein-coding genes that were identified by homology search, 9,590 gene IDs on macrochromosomes and 3,129 on microchromosomes were identifiable by PANTHER16.0 [37, 38] using the protein family/subfamily library (Supplementary Fig. S2). These genes were classified into a total of 164 pathways from ∼177 available pathways in PANTHER. The highest number of genes belonged to the “Wnt signaling pathway (P00057)” and “Gonadotropin-releasing hormone receptor pathway (P06664),” which together accounted for >10% (>5% each) of the macrochromosomal and microchromosomal genes. We compared the frequencies of genes in each PANTHER pathway between macrochromosomes and microchromosomes and found 37 pathways where all genes were located on macrochromosomes (Supplementary Table S4), with 13 pathways having all genes localized to a single macrochromosome. Among microchromosomes, we found that 3 pathways have genes exclusively found on only microchromosomes, and in all 3 pathways, these genes were located on a single microchromosome (Supplementary Table S4). These 40 pathways (37 for macrochromosomes and 3 for microchromosomes) mostly belong to biosynthesis, signaling, metabolism, and degradation pathways (in descending order).

### Synteny analysis

**Figure 3:**
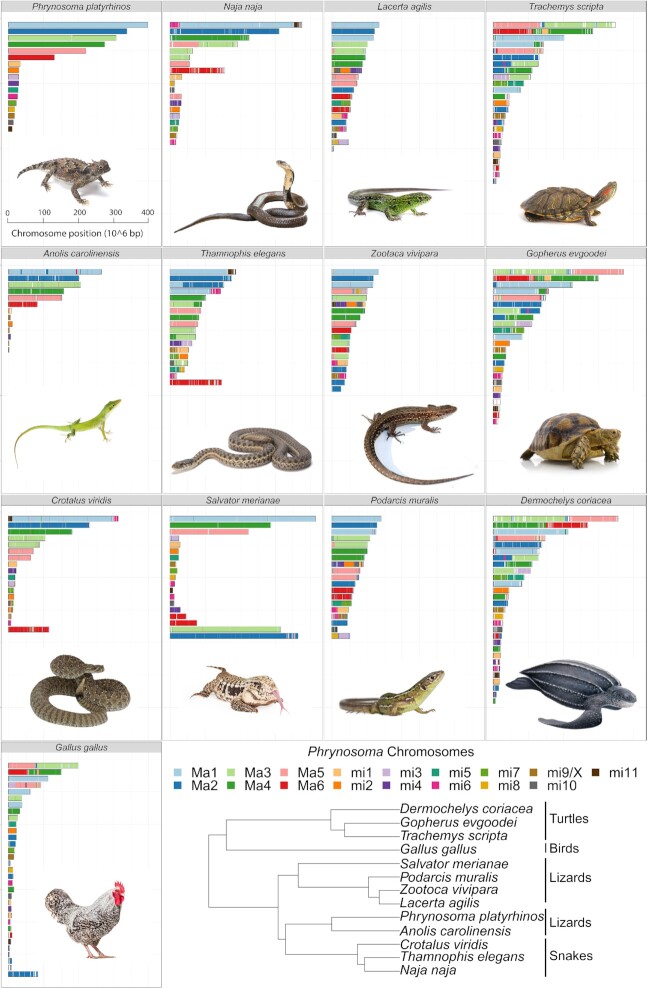
Synteny between *P. platyrhinos* and 12 reptilian taxa: 3 snakes (*N. naja, T. elegans*, and *C. viridis*), 5 lizards *(A. carolinensis, L. agilis, Z. vivipara, P. muralis*, and *S. merianae*), 3 turtles (*T. scripta, G. evgoodei*, and *D. coriacea*), and 1 bird (*G. gallus*). The cladogram shows the phylogenetic relationships among the sampled taxa [80] (2 scaffolds for macrochromosome 3 [3a and 3b] are concatenated in this figure).

**Figure 4:**
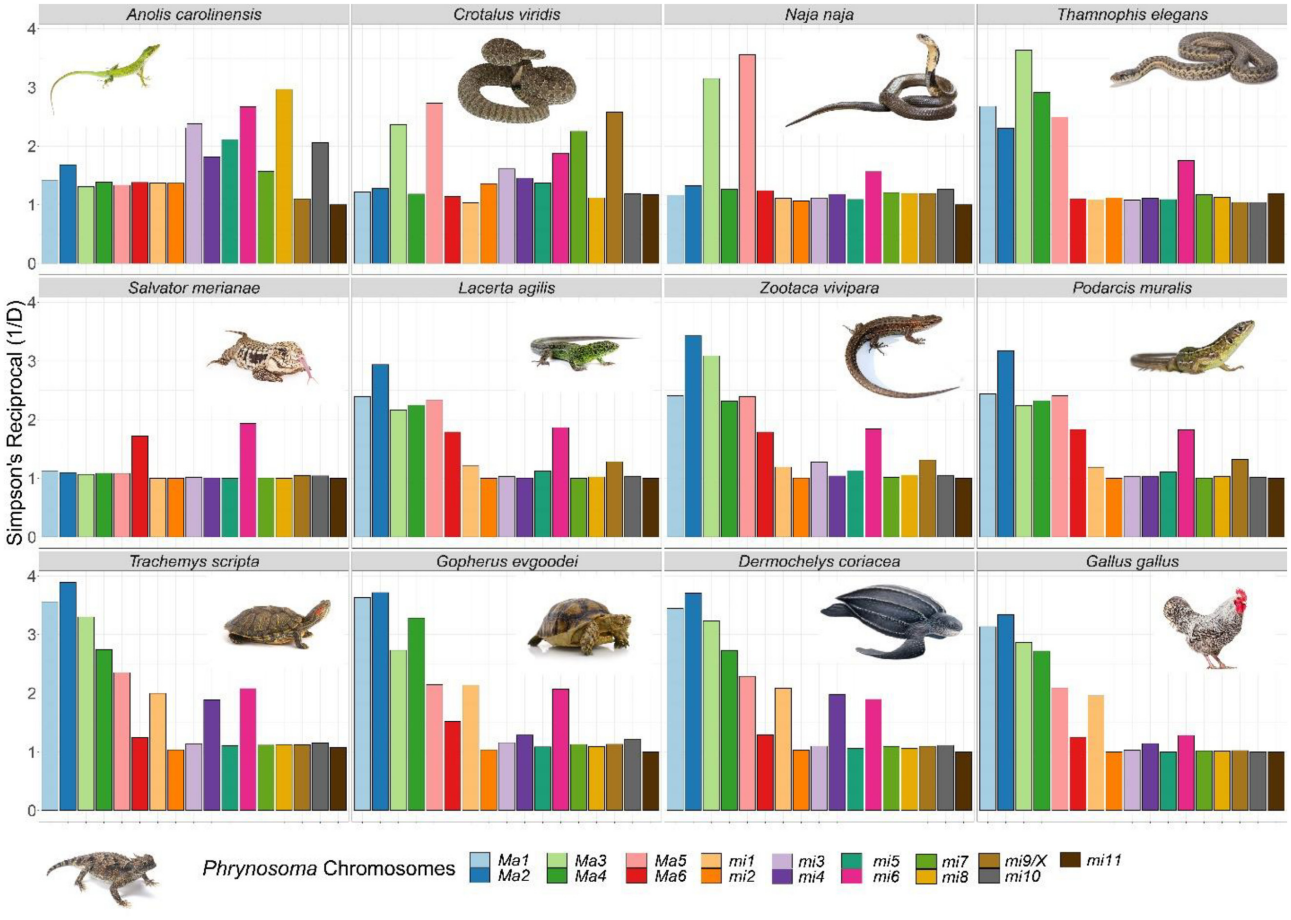
Effective number of chromosomes (C) assessed using the dominance analysis. Values close to 1 represent full dominance (homologies from a given *P. platyrhinos* chromosome are contained within a single chromosome/scaffold of another species). Values >1 mean a spread of homologies across multiple chromosomes/scaffolds.

We investigated how reptilian genome composition has been affected by chromosomal rearrangements through evolutionary time using comparative synteny analyses among reptiles. We conducted pairwise analyses of synteny between the *P. platyrhinos* genome and 12 species (5 lizards, 3 snakes, 3 turtles, and 1 bird) for which chromosome-level genome assemblies were available (Fig. 3) [25]. The genome of *S. merianae* has not been assembled to chromosomes, but the karyotype of this species is known (5 macrochromosomes and 14 microchromosomes [39]), so in this study we used the 19 largest scaffolds from the *S. merianae* assembly (with 5 scaffolds > 200 Mb and 75 Mb > 14 scaffolds > 6 Mb). We performed synteny analyses using a “chromosome painting” technique (see Methods), which established homology between sets of 100-bp *in silico* “markers” from the *P. platyrhinos* chromosome scaffolds and regions of the genomes of the other reptile species (Supplementary Table S5). We quantitatively assessed the degree to which syntenic blocks from each *P. platyrhinos* chromosome scaffold are dispersed across chromosomes of the other species (Fig. 4) using a dominance analysis [40], more commonly used in ecological community assessments. Specifically, dispersion was measured using the Simpson Dominance Index reciprocal (SR), with which we consider an effective number of target chromosomes in other species onto which the homologies of a given *P. platyrhinos* chromosome appear. This index ranges from 1 to *m*, where *m* is the number of chromosomes of the target species being compared to *P. platyrhinos*. A value of 1 represents high dominance, which in this context indicates that syntenic blocks from a chromosome of *P. platyrhinos* are restricted to a single chromosome of another species. A value of *m* would mean that all chromosomes of the target species contain an even proportion of *P. platyrhinos* syntenic blocks. If a large syntenic block is retained in 1 chromosome while a few proportionally small syntenic blocks are distributed across other target chromosomes, the resulting dominance value will trend toward 1.

**Figure 5:**
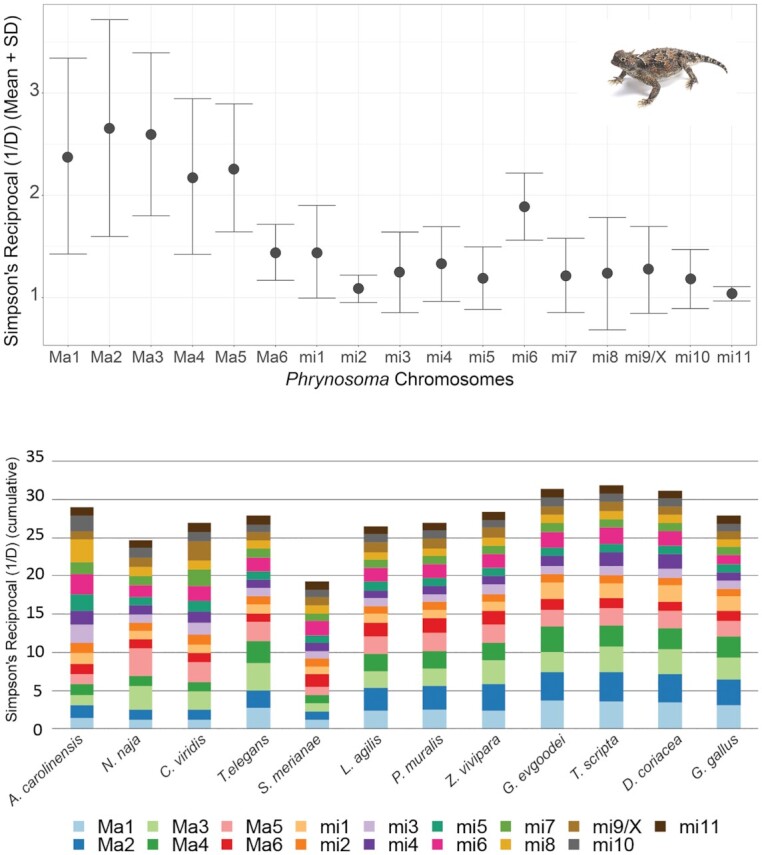
Summary of the effective number of chromosomes of *P. platyrhinos* in comparison with the 12 target species based on SR. (top) Mean (points) and SD (error bars) of SR for each chromosome among 12 species. Values close to 1 represent full dominance (homologies from a given *P. platyrhinos* chromosome are contained within a single chromosome/scaffold). Values >1 mean a spread of homologies across multiple chromosomes/scaffolds. (bottom) Cumulative SR for chromosomes of 12 reptilian species. The total amount of SR at greater phylogenetic distances is higher (cumulative SR ∼ 30 in turtles) and showing greater rearrangements and partitions of syntenic blocks in macrochromosomes than in microchromosomes.

Our results show that macrochromosomes tend to have a higher degree of dispersion across different chromosomes of other species than microchromosomes (e.g., macrochromosome 1 SR = 2.38 [SD 0.96]; microchromosome 1 SR = 1.45 [SD 0.45]), except for macrochromosome 6 (SR = 1.44 [SD 0.27]; Fig. 5,top). However, this chromosomal rearrangement does not follow the same pattern across species (Fig. 4). For example, *A. carolinensis* shows the highest values for SR in microchromosomes (Fig. 5, bottom), but this may be an artifact of this species having an incomplete genome assembly for microchromosomes. In other lizards and snakes (with the exception of *C. viridis*), SR ∼ 1 for all microchromosomes (except microchromosome 6). In *G. gallus*, SR ∼ 1 for all microchromosomes except microchromosome 1. In turtles, mean SR values for microchromosomes are >1, but this is largely driven by higher SR values on microchromosomes 1, 4, and 6 (Fig. 4).

Macrochromosome synteny seems highly conserved between *P. platyrhinos* and *S. merianae*. Among the closest relatives of *P. platyrhinos, A. carolinensis* has the same macrochromosome arrangement as *P. platyrhinos* (Figs. 3–5). In the more distantly related snakes, *N. naja* and *C. viridis*, however, macrochromosomes 3 and 5 show high SR values and the remaining macrochromosomes have SR ∼ 1. Compared to the other snakes, *T. elegans* (along with lizards in the family Lacertidae) generally possess a greater number of smaller macrochromosomes than *P. platyrhinos* and associated higher SR values. At greater phylogenetic distances, the breakdown of chromosomal synteny from lizards to other reptilian lineages becomes more apparent (cumulative SR ∼ 30 in turtles) and showing greater rearrangements and partitions of syntenic blocks in macrochromosomes than in microchromosomes (Figs 4 and 5).

Our results also show that rearrangements between macro- and microchromosomes are apparently common throughout the evolution of Reptilia, including macro- and microchromosomes fusing together to form single macrochromosomes. For example, microchromosomes 5 and 6 in *P. platyrhinos* form a macrochromosome in *L. agilis, Z. vivipara*, and *P. muralis*; chromosome 6 of *P. platyrhinos* is syntenic with a macrochromosome and a microchromosome in *S. merianae;* and microchromosome 6 of *P. platyrhinos* comprises 2 microchromosomes in *S. merianae, G. gallus*, and turtle species (Fig. 3).

## Discussion

The *P. platyrhinos* genome is only the second chromosome-level assembly available for the diverse lizard family Iguanidae (after *A. carolinensis*), and the only member of this family with well-assembled microchromosomes, thereby contributing a new valuable resource for comparative genomics of reptiles. For *P. platyrhinos*, we identified scaffolds representing the 6 macrochromosomes and 11 microchromosomes that comprise the known karyotype for the genus *Phrynosoma* [27, 28, 41]. We note that the chromosome number designations especially for microchromosomes, however, may differ from that of the known karyotype owing to multiple factors, including the lack of chromosome-linked markers for individual microchromosomes, our post hoc bioinformatic-driven inferences of microchromosome boundaries, and the completeness of our genome assembly potentially affecting the accuracy of estimates of the true relative sizes (and size differences) of all microchromosomes. Despite this, the higher contiguity and completeness of microchromosomal scaffolds in the *P. platyrhinos* genome relative to that of *A. carolinensis* does enable some of the first comparisons of chromosome evolution in lizards that incorporates patterns distinct to macro- versus microchromosomes. Our analyses of this and other comparative reptilian genomes highlight distinct functional classes of genes, chromosomal structure, and rearrangement patterns in microchromosomes compared with macrochromosomes.

Consistent with previous studies of reptilian chromosome composition [8, 10, 42], we find that in *P. platyrhinos*, GC content, GD, and repeat element density differ between macrochromosomes and microchromosomes, with GD and GC content being higher on microchromosomes and repeat elements being more densely distributed on macrochromosomes. Patterns of high GD on microchromosomes have been hypothesized to be an evolutionary solution to reduce overall DNA mass and increase recombination rates between coding regions, predominantly by reducing repeat element content [3]. High recombination rates further increase GC content owing to GC-biased gene conversion [43], leading to a higher frequency of GC bases on microchromosomes that can house functionally different gene content compared with macrochromosomes [13], a pattern that we also observed in the *P. platyrhinos* genome (Fig. 2 and Supplementary Fig. S1).

Our synteny analyses across reptile genomes revealed that splitting, fusion, and rearrangement events among chromosomes have occurred frequently and repeatedly throughout reptile evolution. This pattern of chromosome blocks shifting between macro- and microchromosome linkage likely explains some unusual patterns of GD density, GC content, and repeat elements, such as blocks of high GD on a macrochromosome that may represent ancestral fragments derived from microchromosomes. For example, high GC content and GD relative to other macrochromosomes on 1 end of macrochromosome 6 of *P. platyrhinos* (extending for ∼40 Mb; Fig. 2) supports the scenario that a microchromosomal region with higher gene and GC density was recently translocated to a macrochromosome in the ancestor of *P. platyrhinos*. This process may have also contributed to the observed variation in the numbers and sizes of macro- and microchromosomes, even among closely related species (e.g., *P. platyrhinos* versus *A. carolinensis*, and *C. viridis* versus *T. elegans*). Among macrochromosomes, fusion, splitting, and translocation to other chromosomes in more distantly related species such as turtles and chicken are common, whereas microchromosomes of *P. platyrhinos* typically remain in single homologous blocks in these other reptilian lineages, although there seem to be exceptions based on our analysis (Figs 4 and 5b). Broadly, these findings suggest that ancestral chromosomal rearrangements may have resulted in regions of reptilian genomes that have not yet reached mutational and compositional equilibria, which are otherwise characteristic of macro- and microchromosomal regions, following ancestral chromosomal rearrangement events.

Adding to the growing body of evidence for the structural, compositional, and evolutionary distinctions between micro- and macrochromosomes [10, 13, 44–48], our analyses suggest that the gene content of these 2 classes of chromosomes may be distinct in function. Our preliminary observation of enrichment of genes from certain pathways on individual chromosomes or on macro- and microchromosomes more generally warrants further investigation. These biases could be driven by ancestral contingencies of gene content or active translocations of genes across chromosome classes, which may suggest a functionally driven basis for such biases. Our results, however, need to be interpreted with caution because these pathways are incomplete. Many genes are still functionally unknown, and our genome assembly is partially fragmented and missing some expected genes in Tetrapoda (Table 2). Nevertheless, our inferences, together with other emerging evidence for the compositional and functional distinctiveness between micro- and macrochromosomes [10, 13, 44], suggest that there may be key functional, evolutionary, and mechanistic features that distinguish these chromosome classes that explain the significance of the presence and abundance of microchromosomes across eukaryote lineages.

## Methods

### Genome and transcriptome assembly

We sequenced and assembled the reference genome from a female desert horned lizard collected in Dry Lake Valley, Nevada (NCBI accession SAMN17187150). This specimen was collected and killed according to Miami University Institutional Animal Care and Use Committee protocol 992_2021_Apr. Liver tissue was snap frozen in liquid nitrogen and sent to Dovetail Genomics (Scotts Valley, CA) for extraction of DNA and construction of shotgun, Chicago, and Dovetail Hi-C paired-end libraries. DNA was extracted using buffer G2, and Qiagen protease. Three initial shotgun sequencing libraries were constructed by fragmenting DNA extracts to 475 bp and using a TruSeq PCR-free library prep kit to ligate sequencing adapters and amplify each library. The resulting libraries were sequenced on an Illumina HiSeqX (Illumina HiSeq X Ten, RRID:SCR_016385) and resulted in 859.9 million read pairs from paired-end libraries (totaling 246 Gb; see Table 3 for the number of sequenced reads for each library). Reads were trimmed for quality, sequencing adapters, and mate pair adapters using Trimmomatic (Trimmomatic, RRID:SCR_011848) [49]. Using these data, contigs and small scaffolds were assembled using Meraculous 2.2.4 (diploid_mode 1) (Meraculous, RRID:SCR_010700) [50] with a* k-*mer size of 49-mers, which produced an assembly with a scaffold N50 of 0.013 Mb.

**Table 3:** Sequencing: paired-end libraries used for the genome assembly of *P. platyrhinos*

Library	No. of reads	Assembly version	NCBI accession No.
Shotgun library 1 (150 bp)	311,540,000	Primary	SRR16071941
Shotgun library 2 (150 bp)	239,630,000	Primary	SRR16071940
Shotgun library 3 (150 bp)	308,750,000	Primary	SRR16071939
Chicago library 1 (151 bp)	402,000,000	Intermediate	SRR13811242
Chicago library 2 (151 bp)	398,000,000	Intermediate	SRR13811241
Chicago library 3 (151 bp)	256,000,000	Intermediate	SRR13811240
Hi-C library 1 (151 bp)	332,000,000	Final	SRR13811239
Hi-C library 2 (151 bp)	374,000,000	Final	SRR13811238
Hi-C library 3 (151 bp)	324,000,000	Final	SRR13811237

The original assembly was first scaffolded using a Chicago library according to the manufacturer's protocol. Three Chicago libraries were prepared as described previously [26]. Briefly, for each library, ∼500 ng of high molecular weight genomic DNA was reconstituted into chromatin *in vitro* and fixed with formaldehyde. Fixed chromatin was digested with DpnII, the 5′ overhangs filled in with biotinylated nucleotides, and then free blunt ends were ligated. After ligation, crosslinks were reversed, and the DNA purified from protein. Purified DNA was treated to remove biotin that was not internal to ligated fragments. The DNA was then sheared to ∼350 bp mean fragment size and sequencing libraries were generated using NEBNext Ultra enzymes and Illumina-compatible adapters. Biotin-containing fragments were isolated using streptavidin beads before PCR enrichment of each library. The libraries were sequenced on an Illumina HiSeqX. The number and length of read pairs produced for all libraries was 528 million 2 × 150 bp paired-end reads (see Table 3 for the number of sequenced reads for each library). The resulting scaffolded assembly was far more contiguous, with a scaffold N50 of 63.431 Mb. Last, a final round of scaffolding was performed using data from the Dovetail Hi-C library according to the manufacturer's protocols. Three Dovetail Hi-C libraries were prepared in a similar manner as described previously [51]. Briefly, for each library, chromatin was fixed in place with formaldehyde in the nucleus and then extracted. The following steps were the same as creating Chicago libraries. The number and length of read pairs produced for all libraries was 515 million 2 × 150 bp paired-end reads (see Table 3 for the number of sequenced reads for each library). The input  *de novo  *assembly, Chicago library reads, and Dovetail Hi-C library reads were used as input data for HiRise [52], a software pipeline designed specifically for using proximity ligation data to scaffold genome assemblies. First, Chicago library sequences were aligned to the draft input assembly using SNAP v1.0.0 [53]. The separations of Chicago read pairs mapped within draft scaffolds were analyzed by HiRise to produce a likelihood model for genomic distance between read pairs, and the model was used to identify and break putative misjoins, to score prospective joins, and make joins above a threshold. After aligning and scaffolding Chicago data, Dovetail Hi-C library sequences were aligned and scaffolded following the same method. The final assembly (NCBI accession PRJNA685451) has a length of 1,901.85 Mb with a contig N50 of 12.04 kb and a scaffold N50 of 273.213 Mb (see Table 1 for more statistics for this genome assembly).

Transcriptomic libraries were sequenced from 8 tissues (liver, lungs, brain, muscle, testes, heart, eyes, and kidneys) from a male lizard collected and killed according to Miami University Institutional Animal Care and Use Committee protocol 992_2021_Apr at the same locality as the genome animal. For each library, total RNA was extracted using Trizol reagent, and unstranded mRNAseq libraries were individually prepared using a NEBNext Ultra RNA Library Prep kit with library insert sizes of 250–300 bp and sequenced on an Illumina Hiseq4000 platform (Illumina HiSeq 4000 System, RRID:SCR_016386) using a paired-end 150 bp run by Novogene Corporation, Inc. (Table 4). We used Trinity r2014 0413p1 to assemble transcriptome reads from all tissues (using min_kmer_cov:1 and default settings).

**Table 4:** Number of reads obtained from 8 tissues of *P. platyrhinos*, used for transcriptome assembly

Sample ID	Tissue	Raw Reads	Quality trimmed reads	NCBI accession No.
TRO180600001	Liver	49,736,350	47,699,266	SRR13326553
TRO180600002	Lungs	40,643,066	39,124,052	SRR13326552
TRO180600003	Brain	85,097,044	81,754,486	SRR13326551
TRO180600004	Muscle	37,712,026	34,653,428	SRR13326550
TRO180600005	Testes	62,536,762	58,283,654	SRR13326549
TRO180600006	Heart	34,757,154	32,027,338	SRR13326548
TRO180600007	Eyes	46,140,488	42,334,272	SRR13326547
TRO180600008	Kidneys	41,776,926	38,635,176	SRR13326546

### Chromosome identification

**Figure 6:**
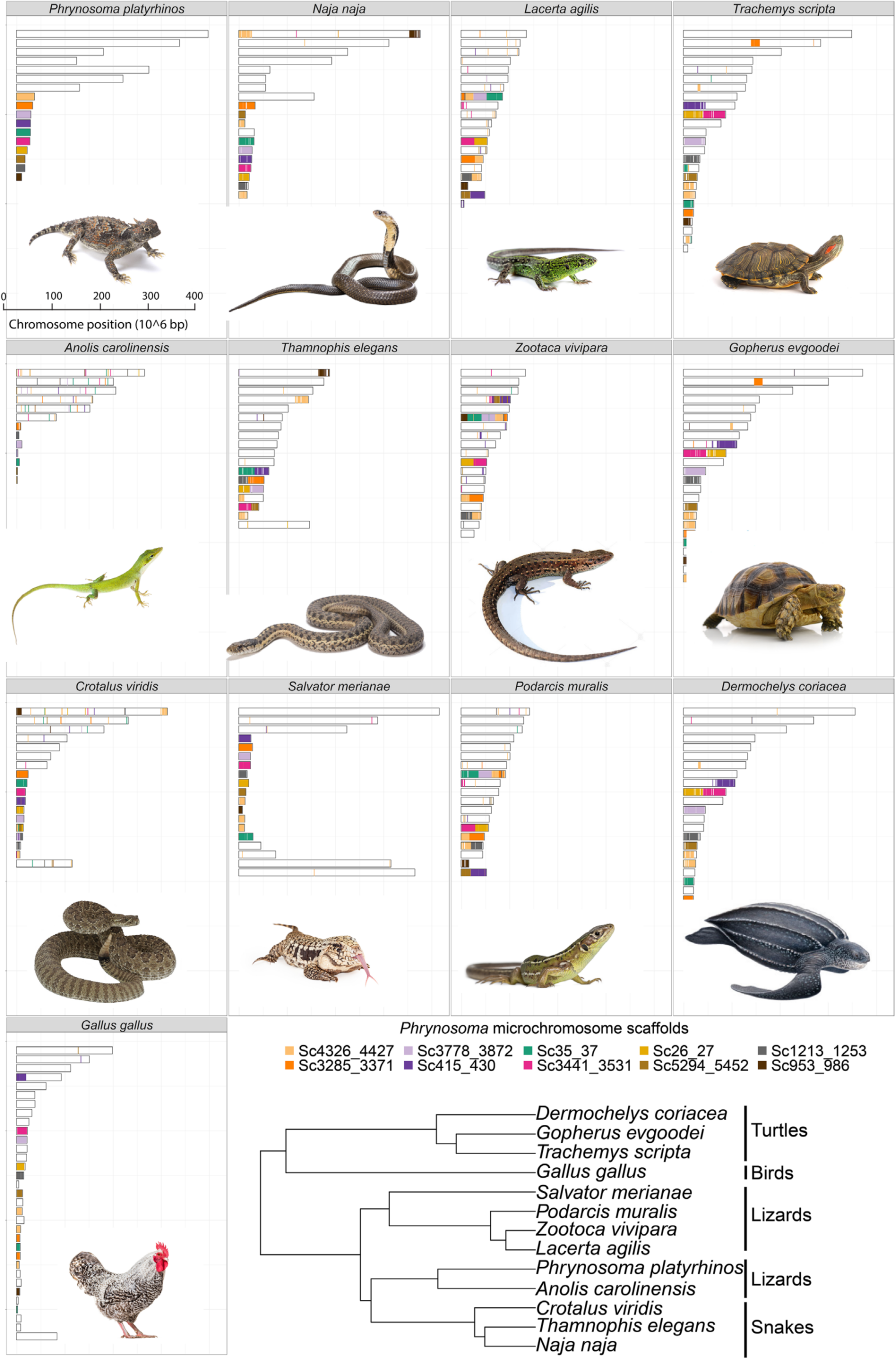
Synteny between *P. platyrhinos* potential microchromosomes (before assigning scaffolds to specific chromosomes) and the 12 reptilian genomes. The cladogram shows the phylogenetic relationships among the assessed taxa [80].

According to the karyotype for phrynosomatid [41] and *P. platyrhinos* [27, 54] (2n = 34), we expected 6 pairs of macrochromosomes and 11 pairs of microchromosomes (1 pair of microchromosomes is expected to be sex linked) for *P. platyrhinos*, and assumed that this karyotype was correct for organizing our scaffolded genome assembly. Assigning scaffolds to specific chromosomes was done using blast+2.8.0 [55] using program “blastx” (options “num_threads” = 4, “-max_target_seqs” = 10, “-evalue” = 1e-5, and “-outfmt” = 11). We used chromosome-linked gene markers in other close species (*A. carolinensis, L. reevesii*) [29] and X-linked markers in *A. carolinensis* [39] downloaded from NCBI (Supplementary Table S1) to identify the genomic location of each gene marker. Available markers for macrochromosomes in lizards were matched to 7 of the largest scaffolds (2 scaffolds for chromosome 3), which we sorted by size and named macrochromosomes 1–6. From the remaining scaffolds, 10 scaffolds (>8 Mb) were selected as potential microchromosomes. This suggested that 1 scaffold comprises 2 microchromosomes fused together because the expected number of microchromosomes was 11. Synteny analysis suggested that scaffold “Scf4326_4427” (Fig. 6) has ≥3 origins in other closely related species. For example, in *S. merianae*, 3 microchromosome account for this scaffold, while the rest of the scaffolds were linked to a specific microchromosome. Given that Chicago libraries reconstitute chromatin *in vitro*, interactions between distinct chromosomes are significantly reduced compared with *in vivo* Hi-C libraries [56]. Also, microchromosomes may have a greater frequency of inter-chromosomal contact [12] than expected in models used to scaffold on the basis of Hi-C sequencing data. Therefore, we scanned for breakpoints between Chicago scaffolds in microchromosome scaffolds, and for each of these breakpoints, we used multiple forms of evidence to assess whether a scaffold should be manually split. Following Schield et al. [8], patterns of GC content, repeat density, and GD at each breakpoint were assessed and we looked for instances in which there were abrupt shifts in these measures near breakpoints between Chicago scaffolds. At 2 of these breakpoints on the putatively artificially merged (with a window of ∼100 bp Ns/gaps) scaffold “Scf4326_4427,” we observed elevated GC content and reduced repeat element density (Supplementary Fig. S3). On the basis of these patterns, we chose to split this scaffold at the breakpoint location with reduced GD to produce a final, curated assembly with the expected number of microchromosomes and finally numbered them on the basis of their size.

### Genome annotation

Repeat elements were first identified using RepeatModeler v. 1.0.11 (RepeatModeler, RRID:SCR_015027) [35] for *de novo* prediction of repeat families. To annotate genome-wide complex repeats, we used RepeatMasker v. 4.0.8 (RepeatMasker, RRID:SCR_012954) [36] with default settings to identify known Tetrapoda repeats present in the curated Repbase database release 20,181,026 [57]. We then ran 2 iterative rounds of RepeatMasker to annotate the known and the unknown elements identified by RepeatModeler, respectively, where the genome sequence provided for each analysis was masked on the basis of all previous rounds of RepeatMasker.

We used MAKER v. 2.31.10 [32] as a consensus-based approach to annotate protein-coding genes in an iterative fashion. For annotation, a genome with complex, interspersed repeats hard masked as Ns was supplied and we set the “model_org” option to “simple” in the MAKER control file (maker_opts.ctl) to have MAKER soft mask simple repeats prior to gene annotation. The full *de novo P. platyrhinos* transcriptome assembly and protein datasets consisting of all annotated proteins for *A. carolinensis* [14] from NCBI were used as the evidence for protein-coding gene prediction. For the first round of annotation, “est2genome” and “protein2genome” were set to 1 to predict genes on the basis of the aligned transcripts and proteins. Using the gene models from the first round of MAKER, we were able to train gene prediction software AUGUSTUS v. 3.2.3. (Augustus, RRID:SCR_008417) [33]. To do so, we used BUSCO v. 2.0.1 (BUSCO, RRID:SCR_015008), which has an internal pipeline to automate the training of Augustus based on a set of conserved, single-copy orthologs for Tetrapoda (Tetrapoda odb9 dataset) [58]. We ran BUSCO in the “genome” mode and specified the “–long" option to have BUSCO perform internal Augustus parameter optimization. Then we ran MAKER with *ab initio* gene prediction ("est2genome = 0” and “protein2genome = 0” options set) using transcripts, proteins, and repeat elements resulting from the first MAKER round as the empirical evidence (in GFF format) to produce gene models using the AUGUSTUS within the MAKER. For all MAKER analyses, we used default settings, except for “trna” (set to 1), “max_dna_len” (set to 300,000), and “split_hit” (set to 20,000). We used the gene models from our second round of MAKER annotation to re-optimize AUGUSTUS as described above before running 1 final MAKER analysis (round 3) with the re-optimized AUGUSTUS settings (all other settings are identical to round 2). We compared annotation edit distance (AED) distributions, gene numbers, and average gene lengths across each round of Maker annotation to assess quality and used our final MAKER round (round 3; N = 20,764 genes) as our final gene annotation.

We ascribed gene IDs based on homology using reciprocal best-blast (with e-value thresholds of 1e−5) and stringent 1-way blast (with an e-value threshold of 1e−8) searches against protein sequences from NCBI for *A. carolinensis, Pogona vitticeps* [59]*, P. muralis* [17], *Gekko japonicus* [60], *Python molurus* [61], *Pseudonaja textilis* [62], *Notechis scutatus* [62], *Protobothrops mucrosquamatus* [63]*, Thamnophis sirtalis* [64]*, Alligator mississippiensis* [65]*, Alligator sinensis* [66, 67]*, Crocodylus porosus* [68]*, Chrysemys picta* [69]*, Terrapene carolina* [70]*, Chelonia mydas* [71]*, Pelodiscus sinensis* [71]*, G. gallus, Homo sapiens* [72]*, Mus musculus* [73], and Swiss-Prot [74] using a custom reciprocal best blast (RBB) script (orthorbb 2.2) [75]. We also searched our annotated transcriptome against the Interpro database via Interproscan–5.36–75.0 [76].

### Pathway analysis

To compare macrochromosomes and microchromosomes functionally, protein-coding genes on each chromosome were analyzed using gene IDs resulting from homology search. An ID list of all annotated genes on each chromosome was used for pathway analysis in PANTHER16.0 (via browser and “Gene List Analysis” tools option) classification system. Four model organisms (*A. carolinensis, G. gallus, M. musculus*, and *H. sapiens*) were selected as the reference for gene IDs. PANTHER assigned each gene to ≥1 of the 164 pathways identified for *P. platyrhinos* genome annotation (with a range of 2–759 genes in each pathway; Supplementary Fig. S4). The distributions of each pathway among different chromosomes were compared using pathway results for each chromosome to identify potential pathways that belong to a specific chromosome or group of chromosomes.

### Synteny and chromosomal composition

We used a Python script “slidingwindow_gc_content.py” [77] to estimate GC content genome-wide in windows of 1 Mb. We estimated gene and repeat element densities for the final genome assembly using Python script “window_quantify.py” with a window size of 1 Mb. Because the distribution of these variables (GD, GC content, repeated elements) was highly skewed/non-normal, we performed Wilcoxon rank sum tests to check for statistically significant differences between macro- and microchromosomes.

We explored broad-scale structural evolution across reptilian genomes using synteny analyses. We obtained chromosome-level genome assemblies from the NCBI database for 5 lizards (*A. carolinensis* [GCA_000090745.2]*, S. merianae* [GCA_003586115.2]*, L. agilis* [GCA_009819535.1]*, P. muralis* [GCA_004329235.1], and *Z. vivipara* [GCA_011800845.1]), 3 snakes (*C. viridis* [GCA_003400415.2], *T. elegans* [GCA_009769535.1], and *N. naja* [GCA_009733165.1]), 1 bird (*G. gallus* [GCA_000002315.5]), and 3 turtles (*T. scripta* [GCA_013100865.1], *G. evgoodei* [GCA_007399415.1], and *D. coriacea* [GCA_009764565.3]).

We used a previously established method for *in silico* painting [44, 78] to partition the *P. platyrhinos* genome to 18.39 million 100-bp markers. As input for this approach, we used blast+2.9.0 to blast the markers against each genome (with “blastn” program and setting “-max_hsps” and “-max_target_seqs” to 1, “outfmt” = 6 qseqid sseqid sstart length pident, “num_threads” = 3, and the rest as default). Following Schield et al. [8], homology signals for chromosome painting had 2 main conditions: (i) each marker should have an alignment length of ≥50 bp and (ii) ≥5 consecutive markers must be present to infer homology (Supplementary Table S5). This was determined for scaffolds from each species. For posterior analyses based on the synteny results, only the assembled chromosomes of each species (based on the reference assembly) were considered. *Salvator merianae* was the only species in our analysis without assembled chromosomes, so we analyzed the 19 longest scaffolds (because karyotype analysis showed 2n = 38) containing the majority of confirmed markers [39].

To assess the distribution of syntenic blocks of *P. platyrhinos* across scaffolds from the 12 target species, we calculated Simpson Dominance Index (D) and its reciprocal, which, in this context, can be considered the effective number of target chromosomes (*C*) containing homologies from a given *P. platyrhinos* chromosome:

\begin{eqnarray*}
{\mathrm{\,\,}}{D_{ij}} = \,\,\mathop \sum\limits_{k\,\, = \,\,1}^m p_{ijk}^2
\end{eqnarray*}

 

\begin{eqnarray*}
{\mathrm{\,\,}}{C_{ij}} = \frac{1}{{{D_{ij}}}},\,\, \end{eqnarray*}

where *i* represents a *P. platyrhinos* chromosome, *j* represents a target species, *m* is the number of scaffolds in the target species *j* containing homologies from the *i*th *P. platyrhinos* chromosome, and *k* represents a specific target scaffold. Values of *D* can range between 0 (low dominance, i.e., high spread of homologies) and 1 (full dominance, i.e., homologies remained in 1 target scaffold). Values of *C* can range between 1 (full dominance) and *m* (low dominance, i.e., equal spread of the *i*th homologies across *m* target scaffolds).

## Data Availability

The chromosome-level genome assembly, annotation files, and other supporting datasets are available in the *GigaScience* database (GigaDB) [79]. The raw genomic and transcriptomic sequencing reads, and genome assembly and annotation are available in the NCBI and can be accessed with BioProject No. PRJNA685451.

## Additional Files

**Figure S1:** Repeat elements, GC content, and gene density calculated in 1-Mb windows for each chromosome of *P. platyrhinos* (2 scaffolds for macrochromosome 3 are concatenated).

**Figure S2:** Proportion of identified gene IDs from protein-coding annotation to unidentified gene IDs by PANTHER (a) across the chromosomes (Ma indicates macrochromosome, and mi, microchromosome) and (b) between 2 groups of chromosomes (Macros = macrochromosomes, and Micros = microchromosomes).

**Figure S3:** Investigating potential misassembled point on a final scaffold. (a) Chicago scaffolds assembled to a final scaffold “Sc4326_4427” were used to investigate a possible misassembled point. (b) Repeat elements, GC content, and gene density calculated in 1-Mb windows were used as evidence to find breakpoint on this final scaffold. Outlined cells are where the breakpoint was placed. Then microchromosomes were numbered on the basis of size, so these 2 scaffolds were numbered as microchromosome 10 (left portion) and microchromosome 6 (right portion).

**Figure S4:** Distribution of *P. platyrhinos* total annotated protein-coding genes with identified IDs in PANTHER database. Among 164 PANTHER pathways assigned to *P. platyrhinos* protein-coding genes, each pathway accounts for a different number of genes (2 < genes per pathway < 759) that may belong to a specific chromosome (24 pathways only on macrochromosomes, and 3 only on microchromosomes) or group of chromosomes (13 pathways only in macrochromosomes group).

**Supplementary Table S1:** The corresponding scaffolds (first column) for each chromosome of*P. platyrhinos* (second column) and scaffold length (third column) in base pairs. *This scaffold was broken down into 2 microchromosomes (6 and 10).

**Supplementary Table S2:** Best blast hits of complementary DNA [29] and * indicates sex-linked markers [30] from *A. carolinensis* and *L. reevesii* against the genome of *P. platyrhinos*.

**Supplementary Table S3:** Number, length, and percentage of annotated repeat elements identified.

**Supplementary Table S4:** Comparison of molecular pathways analysis on macrochromosomes and microchromosomes. Second column shows the specific pathways identified on each chromosome. Third column shows the pathways that belong to specific group of chromosomes.

**Supplementary Table S5:** Genome assemblies and number of markers used for *in silico* painting. All assemblies are available through NCBI under the appropriate accession.

## Abbreviations

AED: annotation edit distance; BLAST: Basic Local Alignment Search Tool; bp: base pairs; BUSCO: Benchmarking Universal Single-Copy Orthologs; C: effective number of target chromosomes; D: Simpson Dominance index; GD: gene density; kb: kilobase pairs; Mb: megabase pairs; NCBI: National Center for Biotechnology Information; SR: Simpson Reciprocal.

## Ethics Approval

All animals were collected and killed according to Miami University Institutional Animal Care and Use Committee protocol 992_2021_Apr.

## Competing Interests

The authors declare that they have no competing interests.

## Funding

This work was supported by startup funds from Miami University to Tereza Jezkova. Keaka Farleigh was supported by the National Science Foundation Graduate Research Fellowship Program (Award No. 2037786).

## Authors' Contributions

N.K. and T.J. designed the project and wrote the first draft of the manuscript. N.K., A.A., K.F., D.C.C., and D.R.S. performed bioinformatics and data analyses. All authors contributed to writing and approved the final manuscript.

## Supplementary Material

giab098_GIGA-D-21-00044_Original_Submission

giab098_GIGA-D-21-00044_Revision_1

giab098_GIGA-D-21-00044_Revision_2

giab098_Response_to_Reviewer_Comments_Revision_1

giab098_Reviewer_1_Report_Original_Submission Hardip Patel -- 4/22/2021 Reviewed

giab098_Reviewer_1_Report_Revision_1 Hardip Patel -- 10/4/2021 Reviewed

giab098_Reviewer_2_Report_Original_Submission Tonia Schwartz -- 5/11/2021 Reviewed

giab098_Reviewer_2_Report_Revision_1 Tonia Schwartz -- 10/14/2021 Reviewed

giab098_Supplemental_Tables_and_Figures
