# Supplementary material for: A chromosome-level genome assembly and annotation of the desert horned lizard, Phrynosoma platyrhinos, provides insight into chromosomal rearrangements among reptiles
Source: Gigascience. 2022 Feb 4;11:giab098. doi: 10.1093/gigascience/giab098 (PMC8848323; doi:10.1093/gigascience/giab098)

GigaScience

A chromosome-level genome assembly and annotation of the Desert Horned Lizard, Phrynosoma platyrhinos, provides insight into chromosomal rearrangements among reptiles

--Manuscript Draft--

**Manuscript Number:**	GIGA-D-21-00044
**Full Title:**	A chromosome-level genome assembly and annotation of the Desert Horned Lizard, Phrynosoma platyrhinos, provides insight into chromosomal rearrangements among reptiles
**Article Type:**	Research
**Funding Information:**	Miami University — Dr Tereza Jezkova
**Abstract:**	Background. The increasing number of chromosome-level genome assemblies has advanced our knowledge and understanding of macroevolutionary processes. Herein, we introduce the genome of the Desert Horned Lizard, Phrynosoma platyrhinos, an iguanid lizard occupying extreme desert conditions of the American Southwest. We conduct analysis of the chromosomal structure and composition of this species and compare these features across genomes of 12 other reptiles (5 species of lizards, 3 snakes, 3 turtles, and 1 bird).<br><br>Findings. The Desert Horned Lizard genome was sequenced using Illumina short paired-end reads, assembled, and scaffolded using Dovetail Genomics Hi-C and Chicago long-range contact data. The resulting genome assembly had a total length of 1,901.85 Mb, scaffold N50 length of 273.213 Mb, and included 5,294 scaffolds. Our chromosome-level assembly includes 6 macrochromosomes and 11 microchromosomes, with a total of 20,764 annotated genes. GC content and gene density were higher across microchromosomes than macrochromosomes, while repeat element distributions showed the opposite trend. Gene ontology analyses indicated that microchromosome and macrochromosome gene content differs significantly in at least six molecular functions. Synteny analysis indicated that large microchromosome blocks are conserved among closely related species, whereas macrochromosomes show evidence of more frequent fusion and fission events, even between closely related species.<br><br>Conclusions: Our analyses provide new evidence for distinct gene content and chromosomal structure in microchromosomes versus macrochromosomes within reptiles. Our results also demonstrate significant karyotypic evolution across Reptilia, with frequent splits, fusions, and rearrangements that have resulted in shuffling of chromosomal blocks between macrochromosomes and microchromosomes.
**Corresponding Author:**	Nazila Koochekian, M.S.<br>Miami University College of Arts and Science<br>Oxford, OH UNITED STATES
**Corresponding Author Secondary Information:**	
**Corresponding Author's Institution:**	Miami University College of Arts and Science
**Corresponding Author's Secondary Institution:**	
**First Author:**	Nazila Koochekian, M.S.
**First Author Secondary Information:**	
**Order of Authors:**	Nazila Koochekian, M.S.
	Alfredo Ascanio
	Keaka Farleigh
	Daren c card

Powered by Editorial Manager® and ProduXion Manager® from Aries Systems Corporation

	Drew R Schield
	Todd A Castoe
	Tereza Jezkova
Order of Authors Secondary Information:	
Additional Information:	
**Question**	**Response**
Are you submitting this manuscript to a special series or article collection?	No
**Experimental design and statistics**<br><br>Full details of the experimental design and statistical methods used should be given in the Methods section, as detailed in our Minimum Standards Reporting Checklist. Information essential to interpreting the data presented should be made available in the figure legends.<br><br>Have you included all the information requested in your manuscript?	Yes
**Resources**<br><br>A description of all resources used, including antibodies, cell lines, animals and software tools, with enough information to allow them to be uniquely identified, should be included in the Methods section. Authors are strongly encouraged to cite Research Resource Identifiers (RRIDs) for antibodies, model organisms and tools, where possible.<br><br>Have you included the information requested as detailed in our Minimum Standards Reporting Checklist?	Yes
**Availability of data and materials**<br><br>All datasets and code on which the conclusions of the paper rely must be either included in your submission or deposited in publicly available repositories (where available and ethically	Yes

appropriate), referencing such data using a unique identifier in the references and in the “Availability of Data and Materials” section of your manuscript.<br><br>Have you have met the above requirement as detailed in our Minimum Standards Reporting Checklist?	

Powered by Editorial Manager® and ProduXion Manager® from Aries Systems Corporation

Manuscript
Click here to access/download;Manuscript;Phrynosoma_genome_GigaScienc

A chromosome-level genome assembly and annotation of the Desert Horned Lizard, *Phrynosoma platyrhinos,* provides insight into chromosomal rearrangements among reptiles

Running title: genome of *P. platyrhinos*

Nazila Koochekian (*Correspondence): Department of Biology, Miami University, Oxford, OH, 45056, USA. Email: koochen@miamioh.edu

Alfredo Ascanio: Department of Biology, Miami University, Oxford, OH, 45056, USA. Email: ascaniaa@miamioh.edu

Keaka Farleigh: Department of Biology, Miami University, Oxford, OH, 45056, USA. Email: farleik@miamioh.edu

Daren C. Card: Department of Organismic & Evolutionary Biology, Harvard University, Cambridge, MA, 02138, USA, and Museum of Comparative Zoology, Harvard University, Cambridge, MA, 02138, USA. Email: dcard@fas.harvard.edu

Drew R. Schield: Department of Ecology and Evolutionary Biology, University of Colorado, Boulder, CO, 80309, USA. Email: Drew.Schield@colorado.edu

Todd A. Castoe: Department of Biology, University of Texas at Arlington, Arlington, TX, 76019, USA. Email: todd.castoe@uta.edu

Tereza Jezkova: Department of Biology, Miami University, Oxford, OH, 45056, USA. Email: jezkovt@miamioh.edu

Abstract

Background. The increasing number of chromosome-level genome assemblies has advanced our knowledge and understanding of macroevolutionary processes. Herein, we introduce the genome of the Desert Horned Lizard, *Phrynosoma platyrhinos,* an iguanid lizard occupying extreme desert conditions of the American Southwest. We conduct analysis of the chromosomal structure and composition of this species and compare these features across genomes of 12 other reptiles (5 species of lizards, 3 snakes, 3 turtles, and 1 bird).

Findings. The Desert Horned Lizard genome was sequenced using Illumina short paired-end reads, assembled, and scaffolded using Dovetail Genomics Hi-C and Chicago long-range contact data. The resulting genome assembly had a total length of 1,901.85 Mb, scaffold N50 length of 273.213 Mb, and included 5,294 scaffolds. Our chromosome-level assembly includes 6 macrochromosomes and 11 microchromosomes, with a total of 20,764 annotated genes. GC content and gene density were higher across microchromosomes than macrochromosomes, while repeat element distributions showed the opposite trend. Gene ontology analyses indicated that microchromosome and macrochromosome gene content differs significantly in at least six molecular functions. Synteny analysis indicated that large microchromosome blocks are conserved among closely related species, whereas macrochromosomes show evidence of more frequent fusion and fission events, even between closely related species.

Conclusions: Our analyses provide new evidence for distinct gene content and chromosomal structure in microchromosomes versus macrochromosomes within reptiles. Our results also demonstrate significant karyotypic evolution across Reptilia, with frequent splits, fusions, and rearrangements that have resulted in shuffling of chromosomal blocks between macrochromosomes and microchromosomes.

Key words: microchromosome; macrochromosome; gene content; synteny; Reptilia

Background

The increasing number of available chromosome-level genome assemblies of non-traditional model organisms has advanced our understanding of genome evolution over large time scales, including intra- and inter-chromosomal rearrangements and karyotype evolution. Reptiles (herein defined as the clade of Sauropsida) exhibit particularly high levels of karyotypic variation (Fig. 1)[1, 2]. Much of this karyotypic variation is apparently due to frequent merging, splitting, and rearrangements events among chromosomes, resulting in varying numbers and sizes of chromosomes even among closely related taxa. Reptilian karyotypic variation is especially notable with regard to variation in the size and number of microchromosomes (Fig. 1), with microchromosomes having an average length of only 12 Mb in comparison with macrochromosomes that range from 40 to 250 Mb [3]. The presence of microchromosomes span 400–450 million years of evolutionary history, are present in many ancient chordates, fish, and amphibians, and are universally present in all reptiles, except crocodiles [4]. Interestingly, microchromosomes are absent from mammalian genomes and microchromosome organization in avian species is relatively conserved at a karyotypic level, except for occasional fusion to other chromosomes in some species [5]. In contrast, microchromosomes of non-avian reptiles are variable in number and size [6], potentially due to relatively high recombination rates [7] that lead to higher rates of chromosomal rearrangement [4,8]. Despite being a promising system in which to study karyotypic evolution, relatively little is known about the genomic features of macrochromosomes and microchromosomes and how these features evolve across Reptilia [9]. Moreover, microchromosomes are structurally and functionally distinct from macrochromosomes [10] that makes them interesting to study. Despite interest in the patterns and processes underlying chromosome evolution in reptiles, there have remained relatively few high-quality reptile genomes available to study these questions. Specifically for lizards, only four genomes are annotated and assembled at the level of chromosomes: the Green Anole, *Anolis*

carolinensis [11] (with 6 chromosomes and 7 microchromosomal linkage groups), the Viviparous Lizard, *Zootoca vivipara* [12](19 linkage groups), the Sand Lizard, *Lacerta agilis* [13](18 chromosomes and WZ sex chromosome), and the Common Wall Lizard, *Podarcis muralis* [14](18 chromosomes and a Z sex chromosome). There is also a fifth, nearly chromosome level genome assembly for the Argentine Black and White Tegu, *Salvator merianae* [15] (assembled to 4,512 scaffolds).

Here we present a new chromosome-level genome assembly of the Desert Horned Lizard (*P. platyrhinos*). This species is widely distributed across the southwestern deserts of North America, including some of the hottest and driest places on Earth (e.g. Death Valley in the Mojave Desert; [16]). We have annotated the genome and assessed large-scale structure and composition of the genome across macrochromosomes and microchromosomes. Using this new resource, we conduct synteny analyses to explore major changes in genome organization by comparing it with existing chromosome-level annotated genomes of other lizards (*A. carolinensis, S. merianae, L. agilis, Z. vivipara* and *P. muralis*), snakes (*Crotalus viridis* [17], *Thamnophis elegans* [18], and *Naja naja* [19]), a bird (*Gallus gallus* [20]), and turtles (*Trachemys scripta* [21], *Gopherus evgoodei* [22], and *Dermochelys coriacea* [23]). Our findings reveal differences in structure and gene content of macrochromosomes and microchromosomes and highlight numerous chromosomal rearrangements among reptilian lineages.

Analysis

Genome assembly and chromosome identification

The whole genome of *P. platyrhinos* was sequenced at 21,053.74-fold physical coverage using the Dovetail Genomics HiRise sequencing and assembly approach [24] that combines 150 bp paired-end reads from Chicago and Hi-C data (Table 1). The final assembly included 17 scaffolds comprising 99.56% of the genome assembly. Seven large scaffolds were assigned to

macrochromosomes 1-6 (with two scaffolds representing arms of chromosome 3). Ten smaller scaffolds were assigned to microchromosomes 1-11 (one scaffold was split into two microchromosomes) based on their size (Table 2). Since sex chromosomes are conserved across iguanas [25], microchromosome 9 was identified as the sex chromosome based on the homology with sex chromosome in *A. carolinensis.* Also, three X-linked genes in *A. carolinensis* (*ATP2A2*, *FZD10*, and *TMEM132D* [25]; Table S1) were identified on microchromosome 9 in *P. platyrhinos*.

Genome annotation and chromosomal composition

We annotated 20,764 protein-coding genes in the *P. platyrhinos* genome assembly. Overall, gene density (GD) and GC-content tended to be lower on *P. platyrhinos* macrochromosomes (mean = 0.18, and standard deviation = 0.14 for GD; mean = 0.36, and standard deviation = 0.12 for GC) and higher on microchromosomes (mean = 0.27, and standard deviation = 0.16 for GD; mean = 0.39, and standard deviation = 0.028 for GC; Fig. 2). The annotation of repeat elements identified 44.45% of the genome repetitive content (Table S2), and the density of repeat elements tended to be higher on macrochromosomes (mean = 0.45, and standard deviation = 0.056) than on microchromosomes (mean = 0.39, and standard deviation = 0.01; Fig. 2). The highest repeat content was found in Simple sequence repeats (6.90%), L2/CR1/Rex (6.88%), hobo-Activator (5.98%), and Tourist/Harbinger (4.90%) families (Table S2).

Gene ontology

To assess whether macrochromosomes and microchromosomes contain distinct functional classes of genes, we investigated the distribution of gene functional classes across chromosomes. From 8,634 genes on macrochromosomes and 2,251 genes on microchromosomes, PANTHER [26] annotated 5,323 molecular function hits on macrochromosomes and 1,379 on microchromosomes using a protein families/subfamilies

library. These were classified into eight “level 1” molecular functions, at least one of 42 “level 2”, and 142 “level 3” categories (Table S3). Binding and catalytic activity together accounted for more than 70% of the molecular functions of both macrochromosomal and microchromosomal genes, while translation regulator activity, structural molecule activity, and molecular transducer activity accounted for less than 10% of the total molecular function hits (Table S3). For “level 1” and “level 2” GO categories, the relative fraction of genes in particular categories were not significantly different between macro- and microchromosomes. For “level 3” GO categories, we identified significant differences between macrochromosomes and microchromosomes in functional categories including transcription coactivator activity, transcription corepressor activity, integrin binding, phosphatase inhibitor activity, histone deacetylase activity, and organic acid transmembrane transporter activity (Table S3). Interestingly, the frequency of genes with transcription coactivator activity was higher on macrochromosomes while the opposite function, transcription corepressor activity was higher on microchromosomes. Additionally, genes associated with the function ‘histone deacetylase activity as an enzymatic function in gene regulation at the transcriptional level’ [27] were more highly represented on microchromosomes, as were genes with phosphatase inhibitor activity.

Synteny analysis

To investigate how reptilian genome structure and content has been impacted by chromosomal rearrangements through evolutionary time, we conducted detailed analyses of synteny between *P. platyrhinos* genome and those of 12 species of reptiles for which chromosome-level genome assemblies were available. These results showed *A. carolinensis,* the closest relative to *P. platyrhinos,* has the same macrochromosome arrangement but microchromosomes in *S. merianae* have more similarity in arrangement to *P. platyrhinos* microchromosomes (Figs. 3-5; Table S4).

Based on our synteny inferences across species (Fig. 3), we applied dominance analysis [28], more commonly used in ecological community assessments, to quantitatively assess the degree to which syntenic blocks from each chromosome of *P. platyrhinos* are dispersed across chromosomes of the other species (Fig. 4). This dispersion was measured using the Simpson's Dominance Index reciprocal, which we can call an "effective number of target chromosomes" into which the homologies of a *P. platyrhinos* chromosome appear. This index ranges from 1 to *m*, where *m* is the number of chromosomes of the target species being compared to *P. platyrhinos*. A value of 1 represents high dominance, which in this context indicates that syntenic blocks from a chromosome of *P. platyrhinos* are restricted to a single chromosome of another species. A value of *m* would mean all chromosomes of the target species contain an even proportion of *P. platyrhinos* syntenic blocks. If a large syntenic block is retained in one chromosome while a few proportionally small syntenic blocks are distributed across other target chromosomes, our dominance value will tend to 1.

As expected, our results from chromosomal synteny dominance analysis show that *P. platyrhinos* macro- and microchromosomes have lower degrees of chromosomal rearrangement when compared to closely related species (1 to 3 effective chromosomes; Fig. 4). For example, *A. carolinensis* is the closest relative to *P. platyrhinos* (both species belong to the family Iguanidae) and has the highest synteny with *P. platyrhinos, S. merianae* has the second highest synteny with *P. platyrhinos* with 8 (out of 10) identical microchromosomes and identical macrochromosomes, with the exception of chromosome 6 which is split into two microchromosomes in *S. merianae*. Snake chromosomes also have high synteny with those of *P. platyrhinos*, but a noticeable distinction between macro- and microchromosomes becomes evident. For macrochromosomes' synteny, breaks and fusions into other chromosomes (macro and micro) are apparent in comparisons between snake and lizard genomes, indicative of dispersion of these homologies through the genome. However, for microchromosomes' synteny, in particular with the snakes *N. naja* and *T. elegans*, they appear to be constrained or poorly

dispersed through the genome, in comparison to macrochromosomes. This constrain on microchromosomes' synteny is noticeable even when one or multiple microchromosomes appear fused to others in the target species syntenies. At greater phylogenetic distances, the breakdown of chromosomal synteny and homology from lizards to other reptilian lineages becomes more apparent, showing greater rearrangements and partitions of syntenic blocks in macrochromosomes than in microchromosomes (Fig. 3). This is shown clearly by the dominance analyses, in which the macrochromosomes of *P. platyrhinos* are dispersed across a higher number of effective chromosomes in more distantly related species such as turtles or chicken (Fig. 4). Conversely, microchromosomes of *P. platyrhinos* typically remain in single homologous blocks, as the effective number of chromosomes is close to 1 for all but microchromosome 6. Overall, macrochromosomes tend to have a higher degree of dispersion across different chromosomes in other species than microchromosomes (eg. Ma1 = 2.38 ± 0.96; mi1 = 1.45 ± 0.45; Fig. 4), with the exception of macrochromosome 6 (Ma6 = 1.44 ± 0.27).

Discussion

The chromosome-level assembly and annotation of the *P. platyrhinos* genome is only the second of its kind in the family Iguanidae (after *A. carolinensis*) and contributes a new valuable resource for chromosome-level comparative genomics in reptiles. The higher contiguity of the genome assembly for microchromosomes in *P. platyrhinos* relative to that of *A. carolinensis* enables some of the first comparisons of chromosomal evolution in lizards that incorporates patterns distinct to macro- versus microchromosomes. Our results highlight distinct functional classes of gene content, chromosomal structure, and rearrangement patterns in microchromosomes compared to macrochromosomes. Our synteny analyses illustrate that chromosomes in reptiles have undergone a number of substantial splits, fusions, and rearrangements, often resulting in syntenic blocks shifting between macrochromosomes and microchromosomes. This ancestral pattern of chromatin shifting between macro- and

microchromosomes likely explains some unusual patterns of gene density, GC-content, and repeat elements, such as blocks of high gene density on macrochromosome that may represent ancestral fragments derived from microchromosomes. We also find evidence that gene content on microchromosomes and macrochromosomes differs in multiple functional ways, adding a new layer of functional differentiation that distinguish these types of chromosomes to recent accumulating evidence for their structural and functional distinction [10, 21].

Consistent with previous studies of reptilian chromosome composition [6,7,30], we find that in *P. platyrhinos*, GC content, gene density, and repeat element density differ between macrochromosomes and microchromosomes, with gene density and GC content being higher on microchromosomes and repeat elements being more densely distributed on macrochromosomes. Patterns of high gene density on microchromosomes have been hypothesized to be an evolutionary solution to reduce overall DNA mass and increase recombination rate, predominantly by reducing repeat element content [4]. High recombination rates further increase GC content due to GC-biased gene conversion (gBGC) [31], leading to a higher frequency of GC bases on microchromosomes that can represent functionally different gene content [10]. While gene and repeat element density are highly variable along chromosomes, GC content is known to be higher at subtelomeric regions [32], a pattern we also observed in the *P. platyrhinos* genome (Fig. 2). Interestingly, and in contrast to this broad pattern, in some chromosomes (e.g., microchromosome 6), there are regions of high GC content dispersed throughout the chromosome. This may be indicative of recent chromosomal rearrangements and/or translocations. This hypothesis is supported by our synteny analyses that suggest that microchromosome 6 of *P. platyrhinos* comprises two microchromosomes in *S. merianae*, *G. gallus,* and the two turtle species. Similarly, *P. platyrhinos* chromosome 6 has high GC content and gene density relative to other macrochromosomes. Chromosome 6 of *P. platyrhinos* is syntenic with a macrochromosome and a microchromosome in *S. merianae,* and the high gene density on one end of this chromosome (extending for ~40 Mbp; Fig. 2) supports

the scenario that a microchromosomal region with higher gene and GC density was recently translocated to a macrochromosome in the ancestor of *P. platyrhinos*. Broadly, these findings suggest that ancestral chromosomal translocations and fissions may have resulted in regions of reptilian genomes that have not yet reached mutational and compositional equilibria that are otherwise characteristic of macro- and microchromosomal regions.

Our analyses of synteny across reptilian genomes revealed that splitting, fusion, and rearrangement events among chromosomes are common and have occurred frequently and repeatedly throughout reptile evolution. This process has resulted in varying numbers and sizes of macro- and microchromosomes, even among closely related species (e.g., *P. platyrhinos* versus *A. carolinensis*, and *C. viridis* versus *T. elegans*). Furthermore, rearrangements and fusions appear to often occur between macro- and microchromosomes, including examples of macro and microchromosomes fusing together to form a single macrochromosome (e.g., several *P. platyrhinos* microchromosome form a macrochromosome in *L. agilis, Z. vivipara,* and *P. muralis*). Overall, however, syntenic blocks on macrochromosomes appear to have experienced a greater degree of fusion, splitting, and translocation than those from microchromosomes.

Among reptiles, microchromosomes show substantial variation in both number and size among lineages (Fig. 3). Some individual microchromosomes of *P. platyrhinos* appear to be fused in other lineages to form large microchromosomes or macrochromosomes, and portions of *P. platyrhinos* microchromosomes can be found dispersed across macrochromosomes of other species. We also observed evidence for a large-scale rearrangement of syntenic blocks between micro- and macrochromosomes in Lacertid lizards, based on evidence that while some lacertids (*S. merianae*) show high synteny with *P. platyrhinos,* other lacertid lizards (*L. agilis, Z. vivipara,* and *P. muralis*) show evidence of macrochromosomal blocks from *P. platyrhinos* comprising a substantial portion of microchromosomes, and vice-versa. For example,

macrochromosome 8 in *L. agilis* and *P. muralis,* and chromosome 6 in *Z. vivipara* are almost completely comprised of blocks from microchromosomes in *P. platyrhinos* and *S. merianae*. Macrochromosome synteny appears more highly conserved between *P. platyrhinos* and its closest relative, *A. carolinensis,* and between *P. platyrhinos* and *S. merianae*. Snakes as well as the three lizards in the family Lacertidae generally possess a greater number of smaller macrochromosomes than *P. platyrhinos*, whereas *P. platyrhinos*'s macrochromosomes are often syntenic with two different macrochromosomes in snakes and Lacertids. At greater levels of divergence, the macrochromosome organization in turtles is quite distinct from that of lizards and snakes, indicating that a number of fusion/fission events have occurred deep in the ancestral lineages of reptiles.

Our analyses further suggest that the gene content of microchromosomes versus macrochromosomes may be distinct in key functional aspects, including a greater prevalence of genes that play activating or positive regulatory roles being concentrated on macrochromosomes, versus genes with repressive or negative regulatory roles being concentrated on microchromosomes. Genes contained on microchromosomes are enriched for higher transcription corepressor, phosphatase inhibitor, and histone deacetylase functions. These and other signatures of differences in gene function across major chromosome classes (e.g., macrochromosomes having greater density of integrin binding and organic acid transmembrane transporter activity) suggest that further work to explore the mechanistic and evolutionary underpinnings of such biases may provide new insight into the relationships between genome structure and function, and the genomic location of functional classes of genes. These inferences, together with other emerging evidence for the compositional and functional distinctiveness between micro- and macrochromosomes [7,10,29] suggests that there may be key functional, evolutionary, and mechanistic features that distinguish these chromosome classes that explain the significance of the presence, absence, and variable abundance of microchromosomes across eukaryote lineages.

Methods

Genome and transcriptome assembly

We sequenced and assembled the reference genome from a female Desert Horned Lizard collected in Dry Lake Valley, Nevada (NCBI accession SAMN17187150). This specimen was collected and euthanized according to Miami University Institutional Animal Care and Use Committee protocol 992_2021_Apr. Liver tissue was snap frozen in liquid nitrogen and sent to Dovetail Genomics (Chicago, IL) for construction of Chicago and Dovetail Hi-C libraries used for sequencing on Illumnia platform (Table 3). Read data were used for *de novo* genome assembly (NCBI accession PRJNA685451) by HiRise™ scaffolding pipeline (Table 1). Transcriptomic libraries were sequenced from 8 tissues (liver, lungs, brain, muscle, testes, heart, eyes, and kidneys) from a male lizard collected at the same locality as the genome animal (Table 4). For each library, total RNA was extracted using Trizol reagent, and RNAseq libraries were individually prepared and sequenced by Novogene Corporation Inc using an Illumina HiSeq and 150 bp paired-end reads. We used Trinity r2014 0413p1 to assemble transcriptome reads from all tissues (using min_kmer_cov:1 and default settings). The assembly contained 199,541 transcripts comprising 199,500 Trinity-annotated genes, with an average length of 1438 base pairs and an N50 length of 2420 bp.

Chromosome identification

According to the phrynosomatid karyotype [33], 6 pairs of macrochromosomes and 11 pairs of microchromosomes (one pair sex-microchromosome) were expected to be identified for *P. platyrhinos*. Assigning scaffolds to specific chromosomes was done using chromosome gene markers from other close species (*A. carolinensis*, *Leiolepis reevesii*) (Table S1). Best BLAST with chromosome-linked markers in lizards [34] downloaded from NCBI was used to identify the

genomic location of each gene marker. The markers for macrochromosomes in lizards linked to 7 largest scaffolds (2 scaffolds for chromosome 3), which we sorted by size and named macrochromosomes 1-6. From the remaining scaffolds, 10 scaffolds (> 8 Mbp) were selected as potential microchromosomes. This suggested to us that one scaffold comprises two microchromosomes fused together as the expected number of microchromosomes was 11. Synteny analysis suggested that scaffold 8 (Fig. 6) has at least three origins in other closely related species. For example, in *S. merianae,* three microchromosome account for this scaffold, while the rest of scaffolds were linked to a specific microchromosome. GC content, repeat elements rate, and gene density were used as evidence [6] to find a break point on scaffold 8. We found two GC-rich spots on this scaffold, with significantly low repeat elements rate (Fig. S1). We chose the spot with significantly lower gene density to split this scaffold into two microchromosomes. Afterwards, microchromosomes were numbered based on their size. Finally, *A. carolinensis* X-linked markers [35] were used to identify the sex chromosome

Genome annotation

Repeat elements were first identified using RepeatModeler v. 1.0.11 [36] for *de novo* prediction of known and unknown repeat families. To annotate genome-wide complex repeats, we used RepeatMasker v. 4.0.8 [37] with default settings to identify known Tetrapoda repeats present in the curated Repbase database release 20181026 [38]. We then ran 2 iterative rounds of RepeatMasker to also annotate first the known and then the unknown elements identified by RepeatModeler, where the genome sequence provided for each analysis was masked based on all previous rounds of RepeatMasker. We used MAKER v. 2.31.10 [39] as a consensus-based approach to annotate protein-coding genes in an iterative fashion. Also, to annotate simple repetitive elements in the MAKER control file (maker_opts.ctl), we set the ‘model_org’ option to ‘simple’ to have MAKER soft mask them. The full *de novo P. platyrhinos* transcriptome assembly and protein datasets consisting of all annotated proteins for *A. carolinensis* [11] from

NCBI were used as the evidence for protein coding genes. For the first round of annotation, "est2genome" and "protein2genome" were set to 1 to predict genes based on the aligned transcripts and proteins. Using the gene models from the first round of MAKER, we were able to train gene prediction software AUGUSTUS v. 3.2.3. [40]. To do so, we used Benchmarking Universal Single-Copy Orthologs (BUSCOs) v. 2.0.1, which has an internal pipeline to automate the training of Augustus based on a set of conserved, single-copy orthologs for Tetrapoda (Tetrapoda odb9 dataset) [41]. We ran BUSCO in the 'genome' mode and specified the '--long' option to have BUSCO perform internal Augustus parameter optimization. Then we ran MAKER with *ab initio* gene prediction ('est2genome=0' and 'protein2genome=0' options set) using transcripts, proteins, and repeat elements resulted from the first MAKER round as the empirical evidence (in GFF format) to produce gene models using the AUGUSTUS within the MAKER. For all MAKER analyses, we used default settings, except for 'trna' (set to 1), 'max_dna_len' (set to 300,000) and 'split_hit' (set to 20,000). We used the gene models from our second round of MAKER annotation to re-optimize AUGUSTUS as described above before running one final MAKER analysis (round 3) with the re-optimized AUGUSTUS settings (all other settings are identical to round 2). We compared Annotation Edit Distance (AED) distributions, gene numbers, and average gene lengths across each round of Maker annotation to assess quality and used our final MAKER round (round 3; N = 20,764 genes) as our final gene annotation. We ascribed gene IDs based on homology using reciprocal best-blast (with e-value thresholds of 1e-5) and stringent one-way blast (with an e-value threshold of 1e-8) searches against protein sequences from NCBI for *A. carolinensis*, *Pogona vitticeps* [42], *P. muralis* [14], *Gekko Japanese* [43], *Python molurus* [44], *Pseudonaja textilis* [45], *Notechis scutatus* [45], *Protobothrops mucrosquamatus* [46], *Thamnophis sirtalis* [47], *Alligator mississippiensis* [48], *Alligator sinensis* [49], *Crocodylus porosus* [50], *Chrysemys picta* [51], *Terrapene Carolina* [52], *Chelonia mydas* [53], *Pelodiscus sinensis* [53], *G. gallus*, *Homo sapiens* [54], and *Mus*

musculus [55], also against Swiss-Prot [56] and Interpro database [57] using Reciprocal Best Blast (RBB) pipeline (https://darencard.net/blog/2019-01-25-UCSC-genome-track-setup/).

Gene ontology

A list of all annotated genes on each chromosome was used for ontology analysis in PANTHER (http://pantherdb.org/) classification system. PANTHER assigned each gene to one of 8 "level 1" molecular functions on chromosomes: binding (GO:0005488), catalytic activity (GO:0003824), molecular function regulator (GO:0098772), molecular transducer activity (GO:0060089), structural molecule activity (GO:0005198), transcription regulator activity (GO:0140110), translation regulator activity (GO:0045182), and transporter activity (GO:0005215) (Table S3). To be able to observe more detail about the functions of each category, we also compared "level 2" and "level 3" molecular functions between macrochromosomes and microchromosomes.

Synteny and chromosomal composition

GC content, gene density, and repeat elements rate were quantified by breaking each chromosome to 1Gb windows using bedtools-2.28 ("makewindows" option) [58]. We explored broad-scale structural evolution across reptilian genomes using synteny analyses. We obtained chromosome-level genome assemblies from NCBI database (https://www.ncbi.nlm.nih.gov/assembly/?term=reptiles) for five lizards (*A. carolinensis, S. merianae, L. agilis, P. muralis,* and *Z. vivipara*), three snakes (*C. viridis*, *T. elegans*, and *N. naja*), one bird (*G. gallus*), and three turtles (*T. scripta*, *G. evgoodei*, and *D. coriacea*). We used Blackmon's painting method [59] for silico painting to partition the *P. platyrhinos* genome to 18.39 million 100-bp markers. We then used these markers to BLAST (with setting "-max_hsps" and "-max_target_seqs" to 1) against each genome that painted numerous fragments in each genome assembly (Table S4).

Following the synteny analysis approach in Schield et al. (2019), homology signals for chromosome painting had two main conditions: 1) each marker should have an alignment length of 50 bp or greater, and 2) at least five consecutive markers must be present to infer homology (Table S4). This was determined for scaffolds from each species. For posterior analyses based on the synteny results, only the assembled chromosomes of each species were considered. *Salvator merianae* was the only species in our analysis without assembled chromosomes, so we analyzed the 19 longest scaffolds (since karyotype analysis showed 2n=38)[60] containing the majority of confirmed homologies (Table S4).

To assess the distribution of *P. platyrhinos* homologies across scaffolds from the 12 target species, we calculated Simpson's Dominance Index (D) and its reciprocal, which, in this context, can be considered the effective number of target chromosomes (C) containing homologies from a given *P. platyrhinos* chromosome:

$$D_{ij} = \sum_{k=1}^{m} p_{ijk}^2$$

$$C_{ij} = \frac{1}{D_{ij}}$$

Where i represents a *P. platyrhinos* chromosome, j represents a target species, m is the number of scaffolds in the target species j containing homologies from the ith *P. platyrhinos* chromosome, and k represents a specific target scaffold. Values of D can range between 0 (low dominance, i.e. high spread of homologies) and 1 (full dominance, i.e. homologies remained in one target scaffold). Values of C can range between 1 (full dominance) and m (low dominance, i.e. equal spread of the ith homologies across m target scaffolds).

Availability of supporting data and materials

The chromosome-level genome assembly, annotation files, and other supporting data sets are (will be) available in the *GigaScience* database (GigaDB). Raw genomic and transcriptomic sequencing reads were deposited in the NCBI SRA under accession number PRJNA685451.

List of abbreviations

AED: Annotation Edit Distance

BUSCO: Benchmarking Universal Single-Copy Orthologs

C: Effective number of target Chromosomes

D: Simpson's Dominance index

gBGC: GC-Biased Gene Conversion

GD: Gene Density

HAT: Histone Acetyl Transferase

HDAC: Histone Deacetylase

TFs: Transcription Factors

Ethics Approval

All animal collected and euthanized according to Miami University Institutional Animal Care and Use Committee protocol 992_2021_Apr.

Competing interests

The authors declare that they have no competing interests.

Authors' contributions

N.K., T.J., and T.C. designed the project and wrote the manuscript. T.J collected the samples and supervised the project. N.K., A.A., K.F., D.C., and D.S. performed bioinformatics and data analyses. All authors read and approved the final manuscript.

Acknowledgments

This work was supported by startup funds from Miami University to T.J.

References

1. Deakin, J. E., and Ezaz, T. (2019a). Understanding the Evolution of Reptile Chromosomes through Applications of Combined Cytogenetics and Genomics Approaches. *Cytogenetic and Genome Research*, *157*(1–2), 7–20. https://doi.org/10.1159/000495974

2. Gemmell, N. J., Rutherford, K., Prost, S., Tollis, M., Winter, D., Macey, J. R., Adelson, D. L., Suh, A., Bertozzi, T., Grau, J. H., Organ, C., Gardner, P. P., Muffato, M., Patricio, M., Billis, K., Martin, F. J., Flicek, P., Petersen, B., Kang, L., … Stone, C. (2020b). The tuatara genome reveals ancient features of amniote evolution. *Nature*, *584*(7821), 403–409. https://doi.org/10.1038/s41586-020-2561-9

3. Axelsson, E. (2005c). Comparison of the chicken and turkey genomes reveals a higher rate of nucleotide divergence on microchromosomes than macrochromosomes. *Genome Research*, *15*(1), 120–125. https://doi.org/10.1101/gr.3021305

4. Burt, D. W. (2002d). Origin and evolution of avian microchromosomes. *Cytogenetic and Genome Research*, *96*(1–4), 97–112. https://doi.org/10.1159/000063018

5. O'Connor, R. E., Kiazim, L., Skinner, B., Fonseka, G., Joseph, S., Jennings, R., Larkin, D. M., and Griffin, D. K. (2019e). Patterns of microchromosome organization remain highly conserved throughout avian evolution. *Chromosoma*, *128*(1), 21–29. https://doi.org/10.1007/s00412-018-0685-6

6. Schield, D. R., Card, D. C., Hales, N. R., Perry, B. W., Pasquesi, G. M., Blackmon, H., Adams, R. H., Corbin, A. B., Smith, C. F., Ramesh, B., Demuth, J. P., Betrán, E., Tollis, M., Meik, J. M., Mackessy, S. P., and Castoe, T. A. (2019f). The origins and evolution of chromosomes, dosage compensation, and mechanisms underlying venom regulation in snakes. *Genome Research*, *29*(4), 590–601. https://doi.org/10.1101/gr.240952.118

7. Schield, D. R., Pasquesi, G. I. M., Perry, B. W., Adams, R. H., Nikolakis, Z. L., Westfall, A. K., Orton, R. W., Meik, J. M., Mackessy, S. P., and Castoe, T. A. (2020g). Snake Recombination Landscapes Are Concentrated in Functional Regions despite PRDM9. *Molecular Biology and Evolution*, *37*(5), 1272–1294. https://doi.org/10.1093/molbev/msaa003

8. Damas, J., Kim, J., Farré, M., Griffin, D. K., and Larkin, D. M. (2018h). Reconstruction of avian ancestral karyotypes reveals differences in the evolutionary history of macro- and microchromosomes. *Genome Biology*, *19*(1), 155. https://doi.org/10.1186/s13059-018-1544-8

9. Axelsson, E., Webster, M. T., Smith, N. G. C., Burt, D. W., and Ellegren, H. (2005i). Comparison of the chicken and turkey genomes reveals a higher rate of nucleotide divergence on microchromosomes than macrochromosomes. *Genome Research*, *15*(1), 120–125. https://doi.org/10.1101/gr.3021305

10. Perry, B. W., Schield, D. R., Adams, R. H., and Castoe, T. A. (2020j). Microchromosomes Exhibit Distinct Features of Vertebrate Chromosome Structure and Function with Underappreciated Ramifications for Genome Evolution. *Molecular Biology and Evolution*, 1–7. https://doi.org/10.1093/molbev/msaa253

11. Alföldi, J., Di Palma, F., Grabherr, M., Williams, C., Kong, L., Mauceli, E., Russell, P., Lowe, C. B., Glor, R. E., Jaffe, J. D., Ray, D. A., Boissinot, S., Shedlock, A. M., Botka, C., Castoe, T. A., Colbourne, J. K., Fujita, M. K., Moreno, R. G., ten Hallers, B. F., … Lindblad-Toh, K. (2011k). The genome of the green anole lizard and a comparative analysis with birds and mammals. *Nature*, *477*(7366), 587–591. https://doi.org/10.1038/nature10390

12. Yurchenko, A. A., Recknagel, H., and Elmer, K. R. (2020l). Chromosome-level assembly of the common lizard (Zootoca vivipara) genome. *Genome Biology and Evolution*. https://doi.org/10.1093/gbe/evaa161

13. Gemmell, N., Haase, B., Formenti, G., Sims, Y., Wood, J., Howe, K., Mountcastle, J., Lillie, M., Olsson, M., Rhie, A., Fedrigo, O., and Jarvis, E. D. (2019m). Lacerta agilis (Sand lizard) genome, rLacAgi1, primary haplotype. *Vertebrate Genomes Project, G10K, 1230 York Avenue, New York, NY 10065, USA*.

14. Andrade, P., Pinho, C., Pérez i de Lanuza, G., Afonso, S., Brejcha, J., Rubin, C.-J., Wallerman, O., Pereira, P., Sabatino, S. J., Bellati, A., Pellitteri-Rosa, D., Bosakova, Z., Bunikis, I., Carretero, M. A., Feiner, N., Marsik, P., Paupério, F., Salvi, D., Soler, L., … Carneiro, M. (2019n). Regulatory changes in pterin and carotenoid genes underlie balanced color polymorphisms in the wall lizard. *Proceedings of the National Academy of Sciences*, *116*(12), 5633–5642. https://doi.org/10.1073/pnas.1820320116

15. Roscito, J. G., Sameith, K., Pippel, M., Francoijs, K.-J., Winkler, S., Dahl, A., Papoutsoglou, G., Myers, G., and Hiller, M. (2018o). The genome of the tegu lizard Salvator merianae : combining Illumina, PacBio, and optical mapping data to generate a highly contiguous assembly. *GigaScience*, *7*(12), 1–13. https://doi.org/10.1093/gigascience/giy141

16. Jezkova, T., Jaeger, J. R., Oláh-Hemmings, V., Jones, K. B., Lara-Resendiz, R. A., Mulcahy, D. G., and Riddle, B. R. (2016p). Range and niche shifts in response to past climate change in the desert horned lizard Phrynosoma platyrhinos. *Ecography*, *39*(5), 437–448. https://doi.org/10.1111/ecog.01464

17. Pasquesi, G. I. M., Adams, R. H., Card, D. C., Schield, D. R., Corbin, A. B., Perry, B. W., Reyes-Velasco, J., Ruggiero, R. P., Vandewege, M. W., Shortt, J. A., and Castoe, T. A. (2018q). Squamate reptiles challenge paradigms of genomic repeat element evolution set by birds and mammals. *Nature Communications*, *9*(1), 2774. https://doi.org/10.1038/s41467-018-05279-1

18. Bronikowski, A., Fedrigo, O., Fungtammasan, C., Rhie, A., Mountcastle, J., Haase, B., Howe,

K., Chow, W., Collins, J., and Jarvis, E. D. (2019r). hamnophis elegans (Western terrestrial garter snake) genome, rThaEle1, primary haplotype. *Vertebrate Genomes Project, G10K, 1230 York Avenue, New York, NY 10065, USA*.

19. Suryamohan, K., Seshagiri, S., and Guillory, J. (2019s). Nana_v5. *Molecular Biology, Genentech Inc., 1 DNA Way, South San Francisco, CA 94403, USA*.

20. Hillier, L. W., Miller, W., Birney, E., Warren, W., Hardison, R. C., Ponting, C. P., Bork, P., Burt, D. W., Groenen, M. A. M., Delany, M. E., Dodgson, J. B., Chinwalla, A. T., Cliften, P. F., Clifton, S. W., Delehaunty, K. D., Fronick, C., Fulton, R. S., Graves, T. A., Kremitzki, C., … Wilson, R. K. (2004t). Sequence and comparative analysis of the chicken genome provide unique perspectives on vertebrate evolution. *Nature*, *432*(7018), 695–716. https://doi.org/10.1038/nature03154

21. Brian Simison, W., Parham, J. F., Papenfuss, T. J., Lam, A. W., and Henderson, J. B. (2020u). An Annotated Chromosome-Level Reference Genome of the Red-Eared Slider Turtle (Trachemys scripta elegans). *Genome Biology and Evolution*, *12*(4), 456–462. https://doi.org/10.1093/gbe/evaa063

22. Murphy, B., Edwards, T., Rhie, A., Koren, S., Phillippy, A., Fedrigo, O., Haase, B., Mountcastle, J., Lewin, H., Damas, J., Howe, K., Formenti, G., Myers, G., Durbin, R., and Jarvis, E. D. (2019v). G10K-VGP Goodes thornscrub tortoise genome, primary haplotype. *Vertebrate Genomes Project, G10K, 1230 York Avenue, New York, NY 10065, USA*.

23. Omoroske, L., Fedrigo, O., Mountcastle, J., Uliano Da Silva, M., Haase, B., Formenti, G., Chow, W., Howe, K., Gilbert, M. T. P., Flicek, P., Gemmell, N., Marques, T., Scott, A., Murphy, R., Bjorndal, K., Braun, E., Rhie, A., Phillippy, A., and Jarvis, E. D. (2019w). (UPDATE)Dermochelys coriacea (Leatherback Sea Turtle) genome, rDerCor1, primary haplotype, v3. *Vertebrate Genomes Project, G10K, 1230 York Avenue, New York, NY*

10065, USA.

24. Putnam, N. H., O'Connell, B. L., Stites, J. C., Rice, B. J., Blanchette, M., Calef, R., Troll, C. J., Fields, A., Hartley, P. D., Sugnet, C. W., Haussler, D., Rokhsar, D. S., and Green, R. E. (2016x). Chromosome-scale shotgun assembly using an in vitro method for long-range linkage. *Genome Research*, *26*(3), 342–350. https://doi.org/10.1101/gr.193474.115

25. Rovatsos, M., Pokorná, M., Altmanová, M., and Kratochvíl, L. (2014y). Cretaceous park of sex determination: sex chromosomes are conserved across iguanas. *Biology Letters*, *10*(3), 20131093. https://doi.org/10.1098/rsbl.2013.1093

26. Mi, H., Muruganujan, A., Casagrande, J. T., and Thomas, P. D. (2013z). Large-scale gene function analysis with the PANTHER classification system. *Nature Protocols*, *8*(8), 1551–1566. https://doi.org/10.1038/nprot.2013.092

27. Lee, J. W., Cheong, J. H., Lee, Y. C., Na, S. Y., and Lee, S. K. (2000aa). Dissecting the molecular mechanism of nuclear receptor action: Transcription coactivators and corepressors. *Experimental and Molecular Medicine*, *32*(2), 53–60. https://doi.org/10.1038/emm.2000.10

28. Hill, M. O. (1973ab). Diversity and Evenness : A Unifying Notation and Its Consequences Author (s): M . O . Hill Published by : Ecological Society of America DIVERSITY AND EVENNESS : A UNIFYING NOTATION AND ITS CONSEQUENCES '. *Ecology*, *54*(2), 427–432.

29. Schield, D. R., Card, D. C., Hales, N. R., Perry, B. W., Pasquesi, G. M., Blackmon, H., Adams, R. H., Corbin, A. B., Smith, C. F., Ramesh, B., Demuth, J. P., Betrán, E., Tollis, M., Meik, J. M., Mackessy, S. P., and Castoe, T. A. (2019ac). The origins and evolution of chromosomes, dosage compensation, and mechanisms underlying venom regulation in snakes. *Genome Research*, *29*(4), 590–601. https://doi.org/10.1101/gr.240952.118

30. Backstrom, N., Forstmeier, W., Schielzeth, H., Mellenius, H., Nam, K., Bolund, E., Webster, M. T., Ost, T., Schneider, M., Kempenaers, B., and Ellegren, H. (2010ad). The recombination landscape of the zebra finch Taeniopygia guttata genome. *Genome Research*, *20*(4), 485–495. https://doi.org/10.1101/gr.101410.109

31. Huttener, R., Thorrez, L., in't Veld, T., Granvik, M., Snoeck, L., Van Lommel, L., and Schuit, F. (2019ae). GC content of vertebrate exome landscapes reveal areas of accelerated protein evolution. *BMC Evolutionary Biology*, *19*(1), 144. https://doi.org/10.1186/s12862-019-1469-1

32. Dreszer, T. R., Wall, G. D., Haussler, D., and Pollard, K. S. (2007af). Biased clustered substitutions in the human genome: the footprints of male-driven biased gene conversion. *Genome Research*, *17*(10), 1420–1430. https://doi.org/10.1101/gr.6395807

33. Leaché, A. D., and Sites, Jr., J. W. (2009ag). Chromosome Evolution and Diversification in North American Spiny Lizards (Genus *Sceloporus*). *Cytogenetic and Genome Research*, *127*(2–4), 166–181. https://doi.org/10.1159/000293285

34. Srikulnath, K., Nishida, C., Matsubara, K., Uno, Y., Thongpan, A., Suputtitada, S., Apisitwanich, S., and Matsuda, Y. (2009ah). Karyotypic evolution in squamate reptiles: comparative gene mapping revealed highly conserved linkage homology between the butterfly lizard (Leiolepis reevesii rubritaeniata, Agamidae, Lacertilia) and the Japanese four-striped rat snake (Elaphe quadrivirg. *Chromosome Research*, *17*(8), 975–986. https://doi.org/10.1007/s10577-009-9101-7

35. Srikulnath, K., Matsubara, K., Uno, Y., Nishida, C., Olsson, M., and Matsuda, Y. (2014ai). Identification of the linkage group of the Z sex chromosomes of the sand lizard (Lacerta agilis, Lacertidae) and elucidation of karyotype evolution in lacertid lizards. *Chromosoma*, *123*(6), 563–575. https://doi.org/10.1007/s00412-014-0467-8

36. Smit, A., and Hubley, R. (2015aj). *RepeatModeler* (Open-1.0. 2008-2015). http://www.repeatmasker.org

37. Smit, AFA, Hubley, R., and Green, P. (n.d.-ak). *RepeatMasker* (Open-4.0). http://www.repeatmasker.org

38. Bao, W., Kojima, K. K., and Kohany, O. (2015al). Repbase Update, a database of repetitive elements in eukaryotic genomes. *Mobile DNA*, *6*(1), 11. https://doi.org/10.1186/s13100-015-0041-9

39. Cantarel, B. L., Korf, I., Robb, S. M. C., Parra, G., Ross, E., Moore, B., Holt, C., Sanchez Alvarado, A., and Yandell, M. (2007am). MAKER: An easy-to-use annotation pipeline designed for emerging model organism genomes. *Genome Research*, *18*(1), 188–196. https://doi.org/10.1101/gr.6743907

40. Stanke, M., and Morgenstern, B. (2005an). AUGUSTUS: a web server for gene prediction in eukaryotes that allows user-defined constraints. *Nucleic Acids Research*, *33*(Web Server), W465–W467. https://doi.org/10.1093/nar/gki458

41. Simão, F. A., Waterhouse, R. M., Ioannidis, P., Kriventseva, E. V., and Zdobnov, E. M. (2015ao). BUSCO: assessing genome assembly and annotation completeness with single-copy orthologs. *Bioinformatics*, *31*(19), 3210–3212. https://doi.org/10.1093/bioinformatics/btv351

42. Georges, A., Li, Q., Lian, J., O'Meally, D., Deakin, J., Wang, Z., Zhang, P., Fujita, M., Patel, H. R., Holleley, C. E., Zhou, Y., Zhang, X., Matsubara, K., Waters, P., Graves, J. A. M., Sarre, S. D., and Zhang, G. (2015ap). High-coverage sequencing and annotated assembly of the genome of the Australian dragon lizard Pogona vitticeps. *GigaScience*, *4*(1), 45. https://doi.org/10.1186/s13742-015-0085-2

43. Liu, Y., Zhou, Q., Wang, Y., Luo, L., Yang, J., Yang, L., Liu, M., Li, Y., Qian, T., Zheng, Y., Li, M., Li, J., Gu, Y., Han, Z., Xu, M., Wang, Y., Zhu, C., Yu, B., Yang, Y., … Gu, X. (2015aq). Gekko japonicus genome reveals evolution of adhesive toe pads and tail regeneration. *Nature Communications*, *6*(1), 10033. https://doi.org/10.1038/ncomms10033

44. Castoe, T. A., de Koning, A. J., Hall, K. T., Yokoyama, K. D., Gu, W., Smith, E. N., Feschotte, C., Uetz, P., Ray, D. A., Dobry, J., Bogden, R., Mackessy, S. P., Bronikowski, A. M., Warren, W. C., Secor, S. M., and Pollock, D. D. (2011ar). Sequencing the genome of the Burmese python (Python molurus bivittatus) as a model for studying extreme adaptations in snakes. *Genome Biology*, *12*(7), 406. https://doi.org/10.1186/gb-2011-12-7-406

45. Edwards J, R. (2018as). Direct Submission. *UNIVERSITY OF NEW SOUTH WALES, Edwards Lab, E26/2110 BioScience South, UNSW SYDNEY, NSW 2052, Australia*.

46. Aird, S. D., Arora, J., Barua, A., Qiu, L., Terada, K., and Mikheyev, A. S. (2017at). Population Genomic Analysis of a Pitviper Reveals Microevolutionary Forces Underlying Venom Chemistry. *Genome Biology and Evolution*, *9*(10), 2640–2649. https://doi.org/10.1093/gbe/evx199

47. Warren, W. C., and Wilson, R. K. (2015au). Direct Submission. *The Genome Institute, Washington University School of Medicine, 4444 Forest Park, St. Louis, MO 63108, USA*.

48. St John, J. A., Braun, E. L., Isberg, S. R., Miles, L. G., Chong, A. Y., Gongora, J., Dalzell, P., Moran, C., Bed'Hom, B., Abzhanov, A., Burgess, S. C., Cooksey, A. M., Castoe, T. A., Crawford, N. G., Densmore, L. D., Drew, J. C., Edwards, S. V., Faircloth, B. C., Fujita, M. K., … Ray, D. A. (2012av). Sequencing three crocodilian genomes to illuminate the evolution of archosaurs and amniotes. *Genome Biology*, *13*(1), 415. https://doi.org/10.1186/gb-2012-13-1-415

49. Wan, Q., Pan, S., Hu, L., Zhu, Y., Xu, P., Xia, J., Chen, H., He, G., He, J., Ni, X., Hou, H.,

Liao, S., Yang, H., Chen, Y., Gao, S., Ge, Y., Cao, C., Li, P., Fang, L., … Fang, S. (2013aw). The genome sequence of Chinese alligator (Alligator sinensis). *BGI-Shenzhen, Beishan Industrial Zone, Yantian District, Shenzhen, Guangdong 518083, China*.

50. Pham, S., Fiddes, I., Deran, A., Armstrong, J., Rice, E. S., and Paten, B. (2016ax). Comparative assembly of saltwater crocodile genome. *Biomolecular Engineering, University of California, Santa Cruz, 1156 High St., Santa Cruz, CA 95064, USA*.

51. Badenhorst, D., Hillier, L. W., Literman, R., Montiel, E. E., Radhakrishnan, S., Shen, Y., Minx, P., Janes, D. E., Warren, W. C., Edwards, S. V., and Valenzuela, N. (2015ay). Physical Mapping and Refinement of the Painted Turtle Genome (Chrysemys picta) Inform Amniote Genome Evolution and Challenge Turtle-Bird Chromosomal Conservation. *Genome Biology and Evolution*, *7*(7), 2038–2050. https://doi.org/10.1093/gbe/evv119

52. Deem, S. L., and Warren, W. C. (2018az). Direct Submission. *McDonnell Genome Institute, Washington University School of Medicine, 4444 Forest Park, St. Louis, MO 63108, USA*.

53. Wang, Z., Pascual-anaya, J., Zadissa, A., Li, W., and Niimura, Y. (2014ba). *Europe PMC Funders Group The draft genomes of soft – shell turtle and green sea turtle yield insights into the development and evolution of the turtle – specific body plan*. *45*(6), 701–706. https://doi.org/10.1038/ng.2615.The

54. Venter, J. C., Adams, M. D., Myers, E. W., Li, P. W., Mural, R. J., Sutton, G. G., Smith, H. O., Yandell, M., Evans, C. A., Holt, R. A., Gocayne, J. D., Amanatides, P., Ballew, R. M., Huson, D. H., Wortman, J. R., Zhang, Q., Kodira, C. D., Zheng, X. H., Chen, L., … Zhu, X. (2001bb). The Sequence of the Human Genome. *Science*, *291*(5507), 1304–1351. https://doi.org/10.1126/science.1058040

55. Brent, M. R., Birren, B. W., Antonarakis, S. E., Alexandersson, M., Zody, M., Birney, E., Baertsch, R., Cuff, J., Parra, G., Slater, G., Waterston, R. H., Lindblad-Toh, K., Rogers, J.,

Abril, J. F., Agarwal, P., Agarwala, R., Ainscough, R., An, P., Attwood, J., … Lander, E. S. (2002bc). Initial sequencing and comparative analysis of the mouse genome. *Nature*, *420*(6915), 520–562. http://www.nature.com/nature/journal/v420/n6915/full/nature01262.html

56. Bateman, A. (2019bd). UniProt: a worldwide hub of protein knowledge. *Nucleic Acids Research*, *47*(D1), D506–D515. https://doi.org/10.1093/nar/gky1049

57. Blum, M., Chang, H.-Y., Chuguransky, S., Grego, T., Kandasaamy, S., Mitchell, A., Nuka, G., Paysan-Lafosse, T., Qureshi, M., Raj, S., Richardson, L., Salazar, G. A., Williams, L., Bork, P., Bridge, A., Gough, J., Haft, D. H., Letunic, I., Marchler-Bauer, A., … Finn, R. D. (2021be). The InterPro protein families and domains database: 20 years on. *Nucleic Acids Research*, *49*(D1), D344–D354. https://doi.org/10.1093/nar/gkaa977

58. Quinlan, A. R., and Hall, I. M. (2010bf). BEDTools: a flexible suite of utilities for comparing genomic features. *Bioinformatics*, *26*(6), 841–842. https://doi.org/10.1093/bioinformatics/btq033

59. McKenna, D. D., Scully, E. D., Pauchet, Y., Hoover, K., Kirsch, R., Geib, S. M., Mitchell, R. F., Waterhouse, R. M., Ahn, S.-J., Arsala, D., Benoit, J. B., Blackmon, H., Bledsoe, T., Bowsher, J. H., Busch, A., Calla, B., Chao, H., Childers, A. K., Childers, C., … Richards, S. (2016bg). Genome of the Asian longhorned beetle (Anoplophora glabripennis), a globally significant invasive species, reveals key functional and evolutionary innovations at the beetle–plant interface. *Genome Biology*, *17*(1), 227. https://doi.org/10.1186/s13059-016-1088-8

60. da Silva, M. J., de Araújo Vieira, A. P., Galvão Cipriano, F. M., dos Santos Cândido, M. R., de Oliveira, E. H. C., Gimenez Pinheiro, T., and da Silva, E. L. (2020bh). The Karyotype of Salvator merianae (Squamata, Teiidae): Analyses by Classical and Molecular Cytogenetic

Techniques. *Cytogenetic and Genome Research*, *160*(2), 94–99. https://doi.org/10.1159/000506140

61. Hedges, S. B., Dudley, J., and Kumar, S. (2006bi). TimeTree: a public knowledge-base of divergence times among organisms. *Bioinformatics*, *22*(23), 2971–2972. https://doi.org/10.1093/bioinformatics/btl505

FIGURES

Figure 1. Reptile phylogeny adapted from [2]. For each major clade, we list diploid chromosome numbers, macrochromosome numbers, and microchromosome numbers [1]

Figure 2. The genome content of *P. platyrhinos.* The outer circle shows gene density on each chromosome, the middle circle shows repeat element density, and the inner one shows GC content. Each estimate is calculated per 1 million base pair window.

Figure 3. Synteny between *P. platyrhinos* and 12 reptilian taxa: three snakes (*N. naja*, *T. elegance*, and *C. viridis*), five lizards *(A. carolinensis, L. agilis, Z. vivipara, P. muralis*, and *S. merianae*), three turtles (*T. scripta, G. evgoodei*, and *D. coriacea*), and a bird (*G. gallus*). The cladogram shows the phylogenetic relationships among the sampled taxa [61].

Figure 4. Effective number of chromosomes (C) assessed using the dominance analysis. Values close to 1 represent full dominance (homologies from a given *P. platyrhinos* chromosome are contained within a single chromosome/scaffold of another species). Values higher than 1 mean a spread of homologies across multiple chromosomes/scaffolds.

Figure 5. Summary of the effective number of chromosomes (C) of *P. platyrhinos* in comparison with the 12 target species. Values close to 1 represent full dominance (homologies from a given *P. platyrhinos* chromosome are contained within a single chromosome/scaffold). Values higher than 1 mean a spread of homologies across multiple chromosomes/scaffolds.

Figure 6. Synteny between *P. platyrhinos* potential microchromosomes before numbering them and the 12 reptilian genomes. The cladogram shows the phylogenetic relationships among the assessed taxa [61].

Figure S1. Repeat elements, GC content, and gene density calculated in 1Mb windows were used as evidence to find break point on scaffold 8.

TABLES

Table 1. Basic information about the *P. platyrhinos* genome assembly.

Assembly	Chicago Assembly	Chicago + Hi-C Assembly
Longest Scaffold (bp)	361,415,485	396,190.715
Number of Scaffolds	5,458	5,294
Number of Scaffolds > 1 kb	5,458	5,294
Contig N50 (kb)	12.04	12.04
Scaffold N50 (kb)	63,431	273,213
Number of Gaps	258,150	258,317
Percent of Genome in Gaps	1.54%	

Table 2. The length in base pairs for each chromosome of *P. platyrhinos*

Chromosome name	length (in base pairs)
Chromosome 1	396,190,715
Chromosome 2	336,734,411
Chromosome 3-a	178,616,284
Chromosome 3-b	123,146,639
Chromosome 4	273,212,746
Chromosome 5	219,432,639
Chromosome 6	129,273,435
Microchromosome 1	31,685,405
Microchromosome 2	28,086,253
Microchromosome 3	27,277,973
Microchromosome 4	27,087,043
Microchromosome 5	26,097,904
Microchromosome 6	23,702,528
Microchromosome 7	20,466,995
Microchromosome 8	16,009,790
Microchromosome 9/X	15,721,303
Microchromosome 10	11,894,615
Microchromosome 11	8,897,685

Table 3**.** Sequencing libraries used for the genome assembly of *P. platyrhinos.*

**Library**	**Read Type**	**Number of Reads**	**Assembly Version**
Chicago library 1 (151 bp)	paired end	402,000,000	Chicago
Chicago library 2 (151 bp)	paired end	398,000,000	Chicago
Chicago library 3 (151 bp)	paired end	256,000,000	Chicago
Hi-C library 1 (151 bp)	paired end	332,000,000	Chicago + Hi-C
Hi-C library 2 (151 bp)	paired end	374,000,000	Chicago + Hi-C
Hi-C library 3 (151 bp)	paired end	324,000,000	Chicago + Hi-C

692 Table 4. Number of reads obtained from 8 tissues of *P. platyrhinos*, used for transcriptome assembly.

Sample ID	Tissue	Raw Reads	Quality Trimmed Reads	NCBI accession number
TRO180600001	liver	49736350	47699266	SAMN17086063
TRO180600002	lungs	40643066	39124052	SAMN17086064
TRO180600003	brain	85097044	81754486	SAMN17086065
TRO180600004	muscle	37712026	34653428	SAMN17086066
TRO180600005	testes	62536762	58283654	SAMN17086067
TRO180600006	heart	34757154	32027338	SAMN17086068
TRO180600007	eyes	46140488	42334272	SAMN17086069
TRO180600008	kidneys	41776926	38635176	SAMN17086070

693

Table S1. Best BLAST hits of cDNA [34] and *sex linked markers [25] to the genome.

Marker	Accession	Chromosome	Scaffold	E-value
DYNC1H1	AB490348	1q	Chr1	2.95E-179
ESR1	AB490345	1p	Chr1	1.02E-113
WT1	XM_016992885	1	Chr1	2.19E-158
WT1	AB490347	1q	Chr1	7.53E-80
XAB1	AB490344	1p	Chr1	2.31E-35
CHD1	XM_008103079	2	Chr2	0
CHD1	AB480289	2p	Chr2	1.25E-144
DMRT1	XM_003216553	2	Chr2	0
DMRT1	AB480288	2p	Chr2	2.15E-64
GHR	XM_008102837	2	Chr2	0
GHR	AB480290	2p	Chr2	1.01E-104
RPS6	XM_003216606	2	Chr2	5.32E-123
RPS6	AB480287	2p	Chr2	2.39E-88
RUFY1	XM_008104854	2	Chr2	0
RUFY1	AB490352	2q	Chr2	3.45E-22
EIF2S3	XM_003218845	3	Chr3-a	0
EIF2S3	AB490361	3q	Chr3-a	5.58E-104
OCA2	XM_008107106	3	Chr3-a	0
OCA2	AB490360	3q	Chr3-a	1.78E-89
SH3PXD2A	XM_016992171	3	Chr3-b	0
SH3PXD2A	AB490356	3p	Chr3-b	5.98E-166
TLOC1	AB490355	3p	Chr3-b	1.71E-79
HDAC3	XM_003219886	4	Chr4	0
HDAC3	AB490365	4p	Chr4	4.16E-97
RBM12	XM_008109953	4	Chr4	0
RBM12	AB490367	4q	Chr4	3.92E-137
SS18	XM_003219645	4	Chr4	0
SS18	AB490397	4p	Chr4	1.75E-70
ZNF326	XM_008109275	4	Chr4	0

ZNF326	AB490366	4q	Chr4	1.00E-128
ACSL1	XM_008111814	5	Chr5	0
ACSL1	AB490370	5p	Chr5	1.00E-95
DCLK2	XM_008111991	5	Chr5	0
DCLK2	AB490369	5p	Chr5	2.06E-73
EXOC1	XM_008111693	5	Chr5	0
EXOC1	AB490371	5p	Chr5	3.08E-176
RANGAP1	XM_008110743	5	Chr5	0
RANGAP1	AB490374	5q	Chr5	6.70E-80
SOX5	XM_008110345	5	Chr5	0
SOX5	AB490376	5q	Chr5	1.78E-104
UCHL1	XM_003221541	5	Chr5	2.55E-63
UCHL1	AB490372	5p	Chr5	3.46E-59
CTNNB1	AB490379	6q	Chr6	0
GAD2	XM_003222133	6	Chr6	0
GAD2	AB490380	6q	Chr6	1.98E-76
MYST2	AB490378	6p	Chr6	0
WAC	XM_008112381	6	Chr6	0
WAC	AB490381	6q	Chr6	3.60E-159
AR	AB490385	micro	microchr	2.72E-152
TMEM132D*	XM_008113640.2	micro “b”/X	microchr9/X	0
FZD10*	XM_003222753.3	micro “b”/X	microchr9/X	0
ATP2A2*	XM_008113715	micro “b”/X	microchr9/X	0
ATP2A2	AB490391	micro	microchr9/X	4.05E-167
ATRX	AB490386	micro	microchr	7.88E-127
BRD7	AB490390	micro	microchr	3.95E-68
HSPA8	XM_003222794	micro “a”	Chr1	0
HSPA8	AB490395	micro	microchr5	3.70E-162

Table S2. Number, length, and percentage of annotated repeat elements identified using RepeatMasker v. 4.0.8.

Families of repeat elements	Numbers of elements	Length masked (bp)	% of sequence	% element masked
**Retroelements**	2082017	451287018	23.83	20.37
**SINEs**	648720	89280596	4.72	6.35
Penelope	254722	35799757	1.89	2.50
**LINEs**	1311944	319965632	16.90	12.84
L2/CR1/Rex	702907	160952766	8.50	6.88
R1/LOA/Jockey	36	3068	0.00	0.00
R2/R4/NeSL	5129	640551	0.03	0.05
RTE/Bov-B	257696	83172778	4.39	2.52
L1/CIN4	87958	38708200	2.04	0.86
**LTR elements**	121353	42040790	2.22	1.19
BEL/Pao	4074	768559	0.04	0.04
Ty1/Copia	18376	7918963	0.42	0.18
Gypsy/DIRS1	39227	14661509	0.77	0.38
Retroviral	34521	5663234	0.30	0.34
**DNA transposons**	1527111	204435133	10.80	14.94
hobo-Activator	610832	73847731	3.90	5.98
Tc1-IS630-Pogo	314462	42728561	2.26	3.08
PiggyBac	1795	445424	0.02	0.02
Tourist/Harbinger	500329	78020620	4.12	4.90
**Unclassified**	828472	146176330	7.72	8.11
**Total interspersed repeats**	9351681	801898481	42.35	91.51
Small RNA	33490	3376969	0.18	0.33
Satellites	51860	7242936	0.38	0.51
Simple repeats	705413	27116672	1.43	6.90
Low complexity	77452	3957871	0.21	0.76
**Total masked**	10219896	841750763	44.45	100.00

Table S3. Comparison of molecular functions on macrochromosomes and microchromosomes. Red shows statistically different between that group.

Molecular function (GO category)	Number of function hits		Percentage of function hits	
	Macrochr	Microchr	Macrochr	Microchr
**translation regulator activity (GO:0045182)**	**43**	**9**	**0.8**	**0.7**
**transcription regulator activity (GO:0140110)**	**325**	**89**	**6.1**	**6.5**
transcription coregulator activity (GO:0003712)	114	35	33.6	39.8
transcription coactivator activity (GO:0003713)	62	9	64.6	34.6
transcription corepressor activity (GO:0003714)	34	17	35.4	65.4
**molecular transducer activity (GO:0060089)**	**266**	**54**	**5.0**	**3.9**
**binding (GO:0005488)**	**2,041**	**527**	**38.3**	**38.2**
protein-containing complex binding (GO:0044877)	145	40	3.8	3.8
integrin binding (GO:0005178)	15	0	12.4	0
**structural molecule activity (GO:0005198)**	**102**	**22**	**1.9**	**1.6**

**molecular function regulator (GO:0098772)**	**284**	**57**	**5.3**	**4.1**
enzyme regulator activity (GO:0030234)	183	39	67	72.2
phosphatase inhibitor activity (GO:0019212)	3	2	16.7	28.6
**catalytic activity (GO:0003824)**	**1,863**	**519**	**35.0**	**37.6**
catalytic activity, acting on a protein (GO:0140096)	742	213	26.1	27.1
histone deacetylase activity (GO:0004407)	10	7	1.4	3.4
**transporter activity (GO:0005215)**	**399**	**102**	**7.5**	**7.4**
transmembrane transporter activity (GO:0022857)	361	95	89.4	90.5
organic acid transmembrane transporter activity (GO:0005342)	63	7	6.3	2.8
	**5,323**	**1,379**	**100**	**100**

Table S4. Genome assemblies and number of markers used for *in silico* painting.

Organism	Potential single markers	Total confirmed (5 consecutive) markers	Scaffolds with confirmed homologies	Confirmed markers in Scaffolds (%)	Assembly accession
A. carolinensis	2,616,045	87,155	13	57,006 (65.41)	GCF_000090745.1
S. merianae	390,847	31,955	19	31,805 (99.53)	GCA_003586115.2
L. agilis	755,639	44,200	20	44,199 (99.99)	GCF_009819535.1
P. muralis	719,822	46,093	19	45,731 (99.21)	GCF_004329235.1
Z. vivipara	751,121	43,371	19	42,224 (97.35)	GCF_011800845.1
C. viridis	299,173	18,161	18	17,891 (98.51)	GCA_003400415.2
T. elegans	282,458	17,817	18	17,725 (99.48)	GCF_009769535.1
N. naja	291, 209	19,898	19	19,805 (99.52)	GCA_009733165.1
T. scripta	177,241	15,287	25	15,252 (99.77)	GCF_013100865.1
G. evgoodei	152,748	14,864	24	14,614 (98.32)	GCF_007399415.2
D. coriacea	137,161	14,075	29	14,075 (100.00)	GCA_009764565.2
G. gallus	88,397	10,934	33	10,934 (100.00)	GCF_000002315.6

Click here to access/download;Figure;fig1.docx

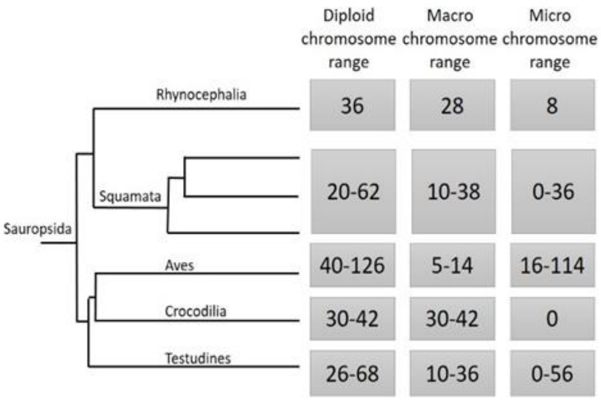

Click here to access/download;Figure;fig2.docx

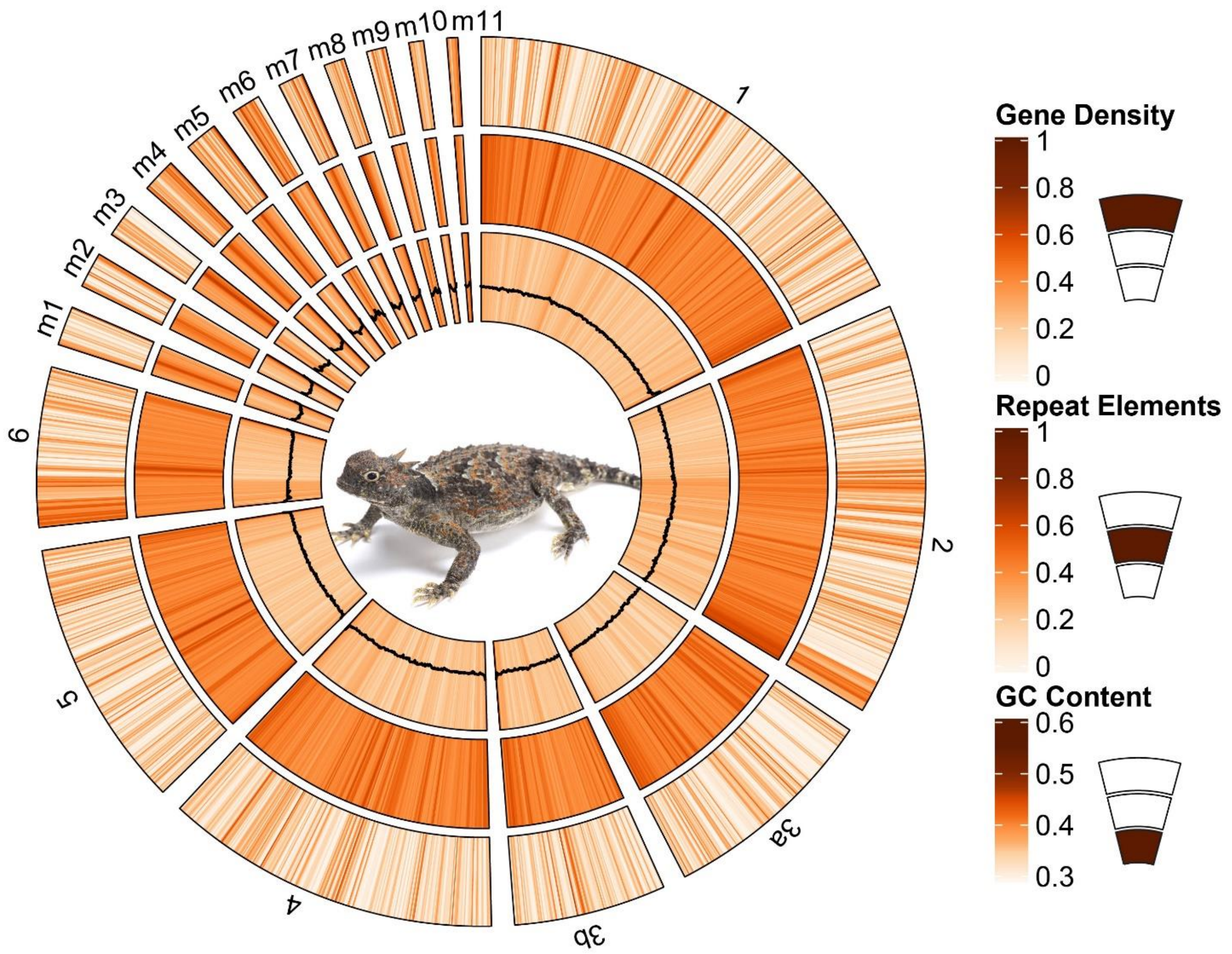

Click here to access/download;Figure;fig3.docx

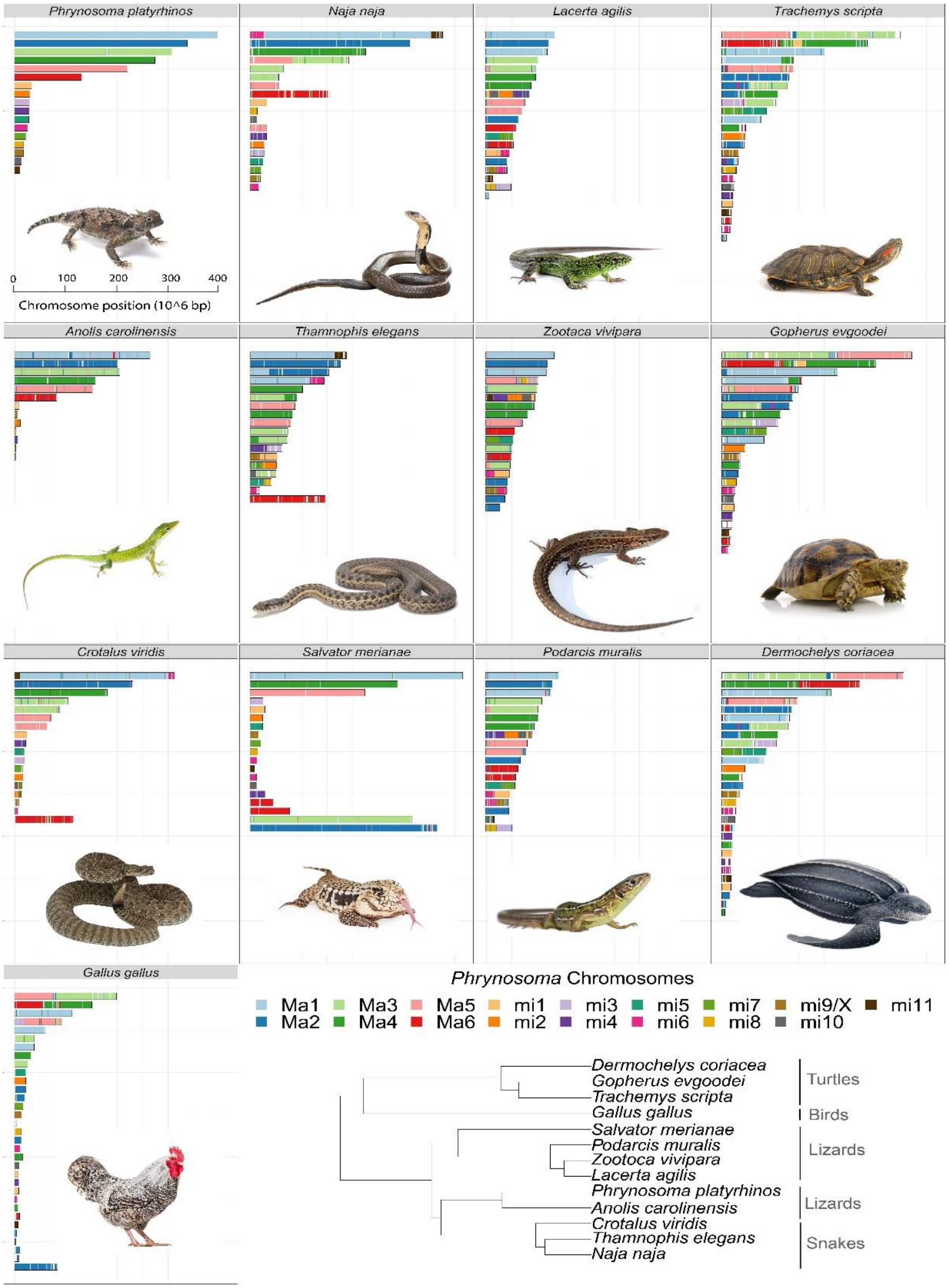

Click here to access/download;Figure;fig4.docx

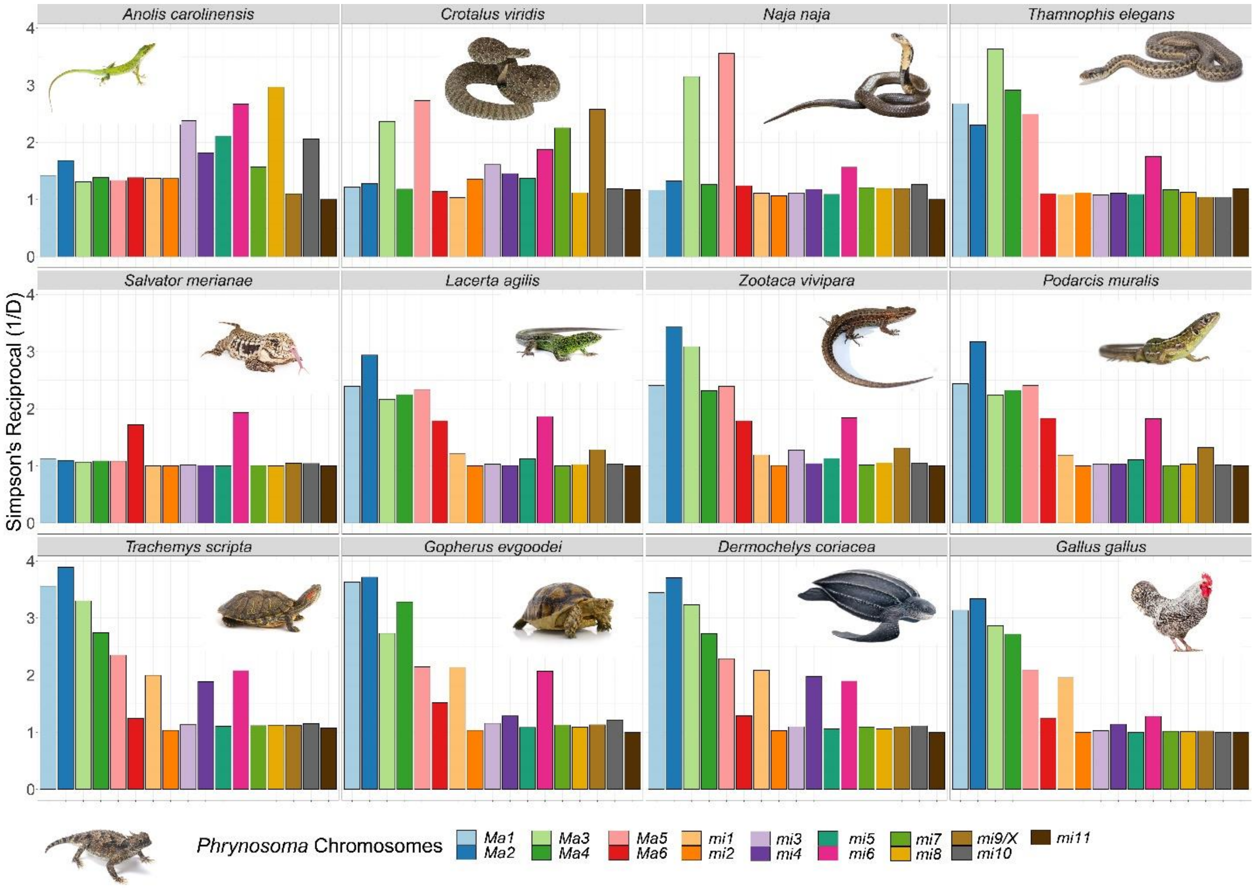

Click here to access/download;Figure;fig5.docx

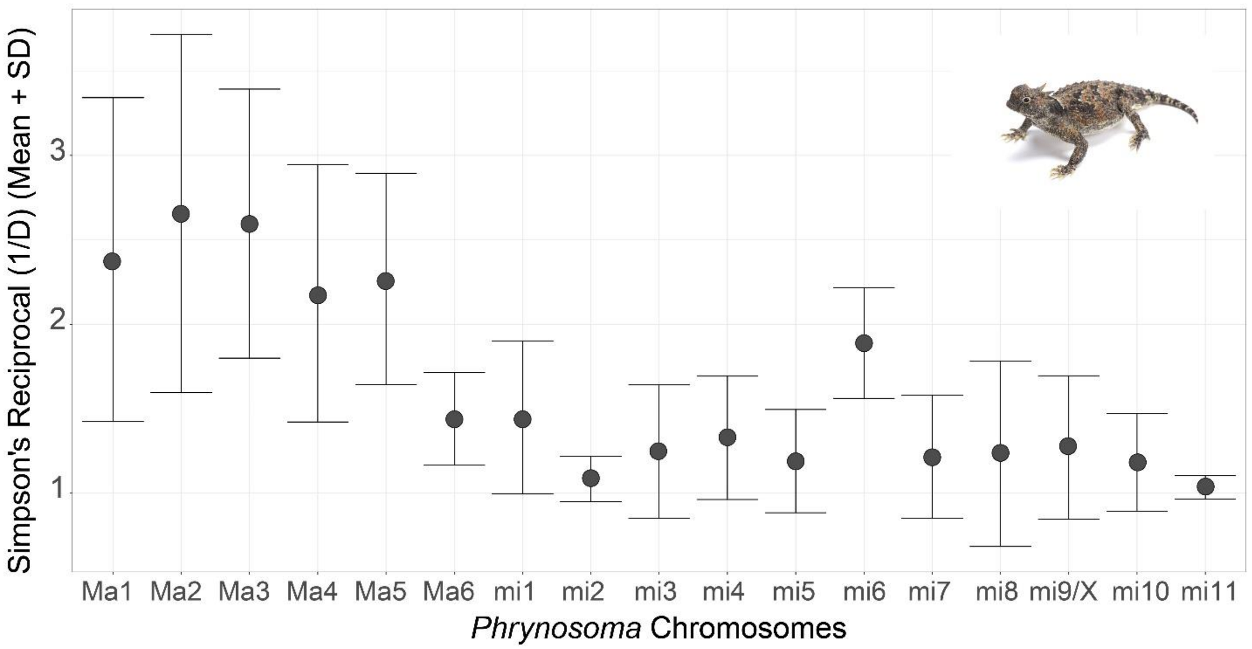

Click here to access/download;Figure;fig6.docx

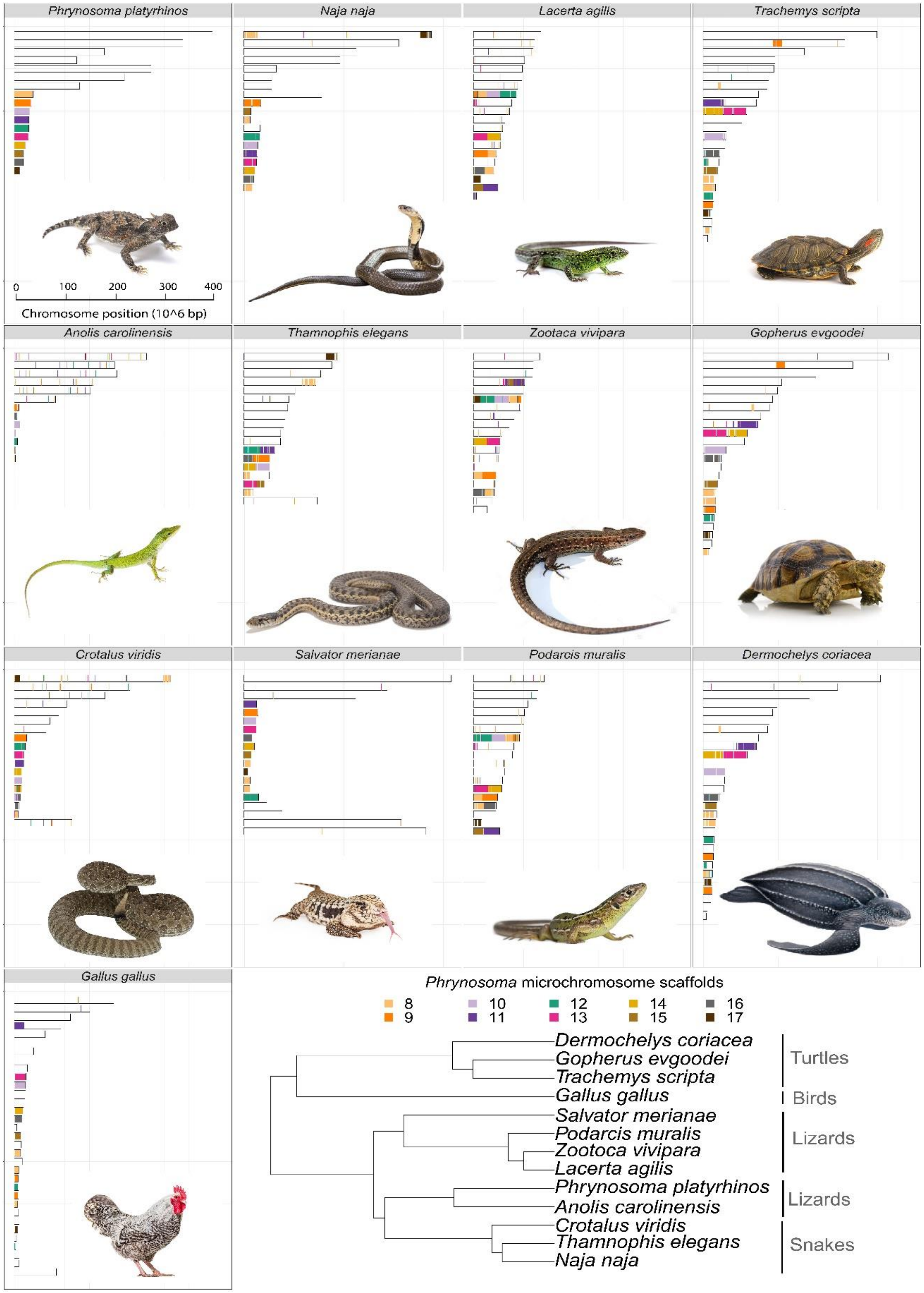

Click here to access/download;Figure;FigS1.docx

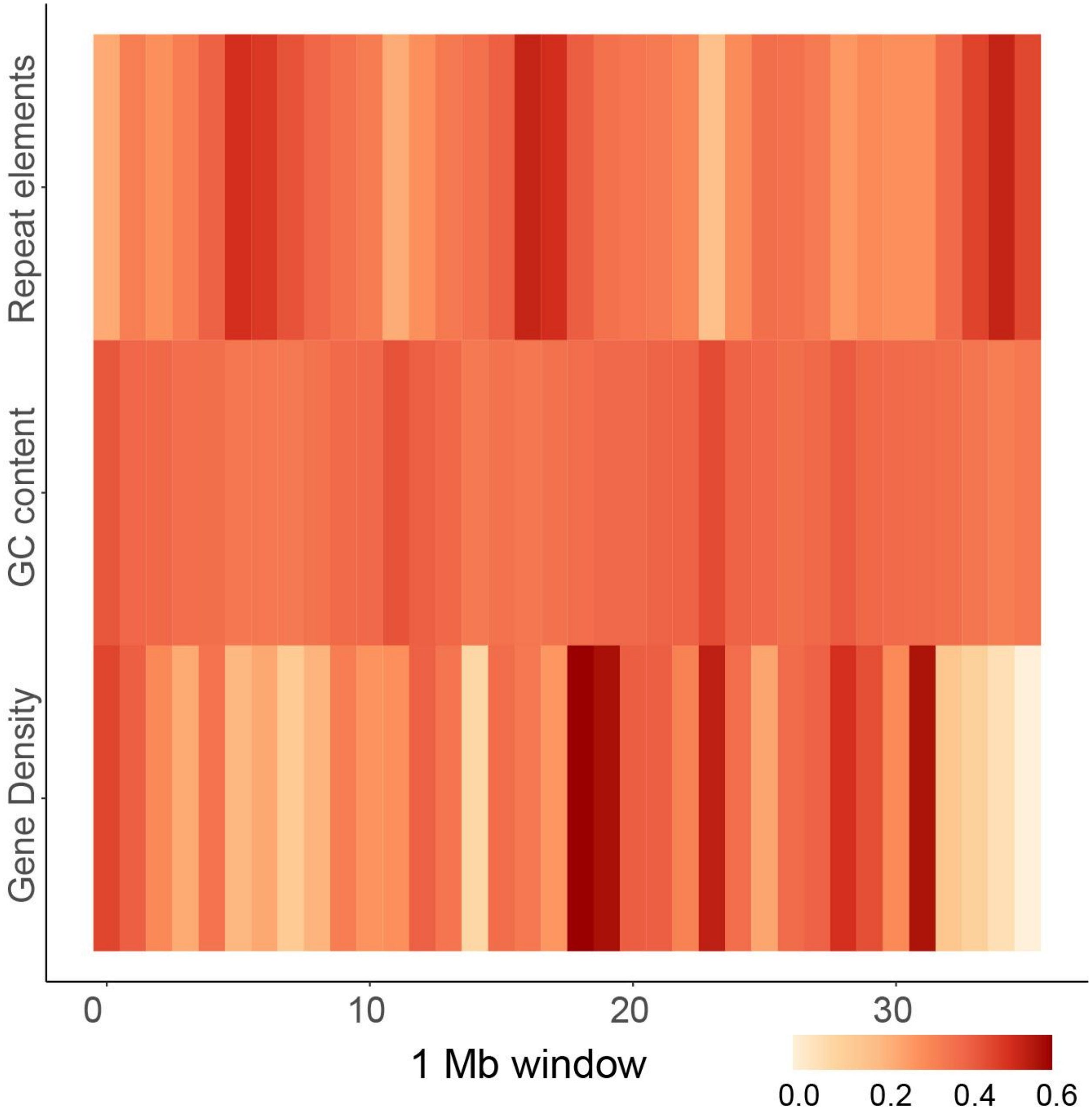

Supplement: giab098_GIGA-D-21-00044_Original_Submission [file giab098_giga-d-21-00044_original_submission.pdf]
