# Supplementary material for: A chromosome-level genome assembly and annotation of the desert horned lizard, Phrynosoma platyrhinos, provides insight into chromosomal rearrangements among reptiles
Source: Gigascience. 2022 Feb 4;11:giab098. doi: 10.1093/gigascience/giab098 (PMC8848323; doi:10.1093/gigascience/giab098)

GigaScience

A chromosome-level genome assembly and annotation of the desert horned lizard, Phrynosoma platyrhinos, provides insight into chromosomal rearrangements among reptiles

--Manuscript Draft--

**Manuscript Number:**	GIGA-D-21-00044R1
**Full Title:**	A chromosome-level genome assembly and annotation of the desert horned lizard, Phrynosoma platyrhinos, provides insight into chromosomal rearrangements among reptiles
**Article Type:**	Research
**Funding Information:**	Miami University — Dr Tereza Jezkova<br>National Science Foundation Graduate Research Fellowship Program (2037786) — Mr Keaka Farleigh
**Abstract:**	Background . The increasing number of chromosome-level genome assemblies has advanced our knowledge and understanding of macroevolutionary processes. Here, we introduce the genome of the desert horned lizard, Phrynosoma platyrhinos, an iguanid lizard occupying extreme desert conditions of the American southwest. We conduct analysis of the chromosomal structure and composition of this species and compare these features across genomes of 12 other reptiles (5 species of lizards, 3 snakes, 3 turtles, and 1 bird).<br>Findings . The desert horned lizard genome was sequenced using Illumina paired-end reads and assembled and scaffolded using Dovetail Genomics Hi-C and Chicago long-range contact data. The resulting genome assembly has a total length of 1,901.85 Mb, scaffold N50 length of 273.213 Mb, and includes 5,294 scaffolds. The chromosome-level assembly is composed of 6 macrochromosomes and 11 microchromosomes. A total of 20,764 genes were annotated in the assembly. GC content and gene density are higher for microchromosomes than macrochromosomes, while repeat element distributions show the opposite trend. Pathway analyses provide preliminary evidence that microchromosome and macrochromosome gene content are functionally distinct. Synteny analysis indicates that large microchromosome blocks are conserved among closely related species, whereas macrochromosomes show evidence of frequent fusion and fission events among reptiles, even between closely related species.<br>Conclusions : Our results demonstrate dynamic karyotypic evolution across Reptilia, with frequent inferred splits, fusions, and rearrangements that have resulted in shuffling of chromosomal blocks between macrochromosomes and microchromosomes. Our analyses also provide new evidence for distinct gene content and chromosomal structure between microchromosomes and macrochromosomes within reptiles.
**Corresponding Author:**	Nazila Koochekian<br>Miami University College of Arts and Science<br>Oxford, OH UNITED STATES
**Corresponding Author Secondary Information:**	
**Corresponding Author's Institution:**	Miami University College of Arts and Science
**Corresponding Author's Secondary Institution:**	
**First Author:**	Nazila Koochekian
**First Author Secondary Information:**	
**Order of Authors:**	Nazila Koochekian<br>Alfredo Ascanio<br>Keaka Farleigh<br>Daren C Card

Powered by Editorial Manager® and ProduXion Manager® from Aries Systems Corporation

	Drew R Schield
	Todd A Castoe
	Tereza Jezkova
**Order of Authors Secondary Information:**	
**Response to Reviewers:**	GIGA-D-21-00044<br>A chromosome-level genome assembly and annotation of the Desert Horned Lizard, Phrynosoma platyrhinos, provides insight into chromosomal rearrangements among reptiles<br>Nazila Koochekian, M.S.; Alfredo Ascanio; Keaka Farleigh; Daren C Card; Drew R Schield; Todd A Castoe; Tereza Jezkova<br>GigaScience<br><br>Reviewer reports:<br><br>Reviewer #1: Authors present the chromosome level genome assembly of the desert horned lizard. They have used Chicago and HiC libraries to construct the genome assembly, which is used for exploratory analyses of chromosomal conservation patterns and describe properties of microchromosomes compositions (repeat, gene and GC%). The manuscript requires substantial revision and report plenty of details that are missing in methods section.<br><br>General comments:<br><br>I had difficulty following the manuscript given the substantial number of technical details were lacking. Broadly, large number of conclusions seemed to be derived from visual observations of figures and graphs without quantitative analyses to back those claims. I also noticed that there were sections in discussions that could be transferred to results (analyses) section. I also found repetitive content in discussions and analyses section. Overall, the manuscript writing style was not up to the level expected of a scientific descriptive paper. Major revision in style of writing is essential to ensure completeness and accuracy of information for readers.<br>*** All coauthors have re-edited the MS to improve clarity, writing quality, and flow. We added details to the methodology (such as assembly and chromosome identification) and revised the writing to reduce repetitive statements. Additionally, because Figures 4 and 5 are used to visualize results from Simpson's Reciprocal analysis, we revised our results and discussion to explain this analysis.<br>Karyotype for the desert horned lizard is assumed from previous study, but not substantiated. Generally, it is OK. However, at least mentioning this in discussion and how those assumptions can have implications in understanding homology are not discussed. Similarly, the quality of the assembly is not verified by any orthogonal method and therefore some of the claims in the manuscript may be wrong. I would encourage authors to discuss their results in the context of the quality of the assembly.<br>*** We have now provided additional citations (27 and 28) showing that the karyotype of the desert horned lizard has been previously estimated by multiple studies. Therefore, we did not re-substantiate this result. We have now also made it clearer that our assembly assumes this karyotype is correct (line 111-113). We have increased discussion in the text about genome assembly, and inferences we made to assign chromosomes to scaffolds. However, we do politely disagree that we have not provided any orthogonal methods to compare our assembly – we have indeed conducted detailed synteny analysis (that shows a great degree of synteny with our genome and others), we have assessed genome completeness and quality in a number of ways (N50 contiguity metrics and BUSCO analyses), and we have shown that except for a small number of minor areas, our independent genome assembly was nearly a perfect match to expectations from multiple previous karyotype studies – all of these orthogonal comparisons are now more clearly pointed out in the text.<br>One of the major points I would like to raise is about the use of genome assemblies that Vertebrate Genome Project (Genome 10K) have generated for this kind of global analyses work. They have an embargo on the use of data as per documentation at https://genome10k.soe.ucsc.edu/data-use-policies/. First, I would like to state that, I am not a member of any of the Genome10K, VGP or other associated projects.<br>The embargo on data use is protected by the Fort Lauderdale Agreement (https://www.genome.gov/Pages/Research/WellcomeReport0303.pdf). Please refer to

Powered by Editorial Manager® and ProduXion Manager® from Aries Systems Corporation

the page 4, section C. "Resource Users" (points 2 and 3) as a shared responsibility by end users of the data. I personally prefer that the Fort Lauderdale Agreement be revised in the interest of research because embargo periods are substantially larger compared to the pace of data production these days. However, we collectively as scientists need to bring this change together in the interest of advancing science fairly for all. I leave it up to authors, journal and the editors to make the judgement call on the use of data that is under embargo.
*** We are aware of the embargo, but we do not conduct any comprehensive analyses of their data except for comparative purposes for the genome we published. It is therefore our understanding that we are in compliance with the Fort Lauderdale Agreement. One of the co-authors is on the Executive Council of the G10k group and they agree that this study does not violate the Ft Lauderdale Agreement from the perspective of their experience with the G10k group.

Specific comments:

Below I provide a list of specific comments.

Title: Please change common name to lower case.
*** Done.
Introduction:
1. Page 3, Line 59-60: Either use average for both or range for both to be consistent
*** We chose range for both (lines 66-68).
2. Line 77: Convention is to say "ZW sex chromosome". Perhaps authors can change if they wish.
***Changed it to “Z and W sex chromosomes” (line 85).
Analysis:
1. Line 109: Mean 0.18. The metric for gene density is unclear. Is it per Mb, or per 100Kb? Also, you have GC on the same fractional scale. Perhaps best to report GC as percentage as it is the widely accepted unit and gene content per Mb (or some other fixed scale). Same is the case for repeat content. Please specify the scales appropriately. Repeats being variable in size as units, perhaps report it as a proportion of the "chromosome" length.
*** Metrics for gene density are per Mb now and GC and repeat elements scales are percent. We now clarify this in the manuscript (lines 145-151).
2. Line 111:112: "elements identified 44.5%" is unclear. Is it that of all repeats, 44.5% repeats were identified and rest 55.5% missing from annotation? Or alternately and probably the case that 44.5% of the genome is composed of repeat elements.
*** The latter is the case, and we rephrased the sentence to make this clear in the revised manuscript (line 140-141).
3. Line 113: If repeat content is compared for macro vs micro, the results don't seem to be significant considering the SD of 0.056 for the macro. 0.45 - 3*0.056 is 0.28, lower than the 0.39 for micro. Differences are not significant I guess on this scale.
*** We redid our statistical analysis using a non-parametric approach, Wilcoxon tests, because the distribution of these variables is highly skewed, and results show these variables are statistically different between macro and microchromosomes (lines 152-155).
4. GO analyses is not performed using statistics. Mere assignments to GO terms is a stretch. Statistical test is not listed. Perhaps authors should list the test.
*** We have updated our functional analysis and used pathways instead of GO terms to make comparisons of present/absent pathways on each group of chromosomes (lines 157-172).
5. Line 178 - 181: Please provide information about what chromosome numbers are you talking about here. It is very difficult to read figures with large number of chromosomes and colors.
*** We agree that there is a lot of information in this figure and in this paragraph. We were trying to move from closest species (lizards) to more distant ones (turtles). We now modified this paragraph (lines 198-208) for clarity and used a new figure (5b) to summarize the information from the previous figures.
Discussion:
1. Line 211: gBGC acronym is used only once. Please remove it.
*** Done.
2. Figure 2: What is the black line for in the inner circle? Circos plots are pleasant to look at but don't convey the message using the heatmaps in this figure. Perhaps

Powered by Editorial Manager® and ProduXion Manager® from Aries Systems Corporation

authors may consider redrawing this figure with a line plot using karyoploteR package in R (https://www.bioconductor.org/packages/release/bioc/html/karyoploteR.html).
*** Since the fluctuation in GC content is small, we had “the black line” to make it easier to follow the increase of GC content across scaffolds (e.g., at scaffold ends). We added heatmap figures in the supplemental information (Fig. S1) and deleted this black line to avoid confusion.
3. Line 214: I had difficulty in observing higher GC content pattern in subtelomeric regions. Authors must provide statistical calculations to show if the pattern is statistically significant or not. Otherwise, they should remove the reference to this discussion point.
*** We agree with the reviewer that a statistical test should be included to support this claim. The issue is that a length of subtelomeric regions has not been defined in the literature. We now adjusted the writing and are no longer make a strong claim about the difference in GC in subtelomeric regions versus elsewhere (lines 247-250).
4. Line 218 - 225 should be migrated to the analysis section.
*** Done (lines 232-234)
5. Line 236: regarding "several microchromosomes", please provide specific chromosome numbers and perhaps think about moving factual informationyou're your observations of the data into result section.
*** We used an alternative way of explaining synteny results (SR numbers) in the revised manuscript and tried to avoid repeating results in discussion section (lines 251-270). So accordingly, “several microchromosomes” is deleted, but we have taken care to report specific details in the remainder of the manuscript.

Methods:
1. Line 284: Ethics approval for the male for transcriptome sequencing is not mentioned. Please list it.
***Done (lines 331-332).
2. Methods are very light on data and assembly generation. This requires major effort. Please see https://academic.oup.com/g3journal/article/10/4/1159/6026169 for an example of how this section should be described. DNA extraction method, fragment size for library prep, read lengths targeted, paired vs single end mode for sequencing, sequencing platform (x10, hiseq2500, novaseq), library method in details.
***Done (lines 286-338).
3. Genome and transcriptome assembly section: Methods are not very clear. It is mentioned that HiRise Scaffolding pipeline was used. No reference, no command line settings, availability of the software is listed.
***We have modified this section of the manuscript to provide all relevant information on genome assembly, including details of software used and how such software was run (lines 319-327).
4. Line 293: Karyotype information is not clear. The paper cited states, "The ancestral 2n = 34 (12M + 20m + XY) phrynosomatid karyotype that is found in several of the basal lineages of Sceloporus differs from the standard iguanian karyotype by the loss of a single pair of microchromosomes.
This to me suggests a karyotype of 6 macro and 10 micro-chromosomes including the sex chromosomes.
***The cited section states that “The ancestral 2n = 36 (12M + 22m + XY) karyotype for iguanian lizards is characterized by 6 pairs of bi-armed macrochromosomes and 12 pairs of micro- chromosomes (two of which are the sex chromosomes). The ancestral 2n = 34 (12M + 20m + XY) phrynosomatid karyotype that is found in several of the basal lineages of Sceloporus differs from the standard iguanian karyotype by the loss of a single pair of microchromosomes.” So, it is explaining that iguanian karyotype is 2n=36 but phrynosomatid karyotype is different from iguanian with one pair less microchromosomes. So, it will be 2n=34 for Phrynosomatidae. We also provide additional citations for the chromosome number in Phrynosomatidae (27 and 28).
Also, the main thesis of the work is about variable karyotype configurations in reptiles. It would be good if authors discussed accuracy of this information. If karyotype cannot be produced, then authors can at least discuss this limitation.
*** The karyotype for P. platyrhinos has been studied before. We added more references to be clear that at the species has a karyotype of 2n=34.
5. Line 296: "Best BLAST" needs to be elaborated clearly with version numbers of assemblies and annotations used for such analyses. Details of software and parameter settings need to be described.
*** Details for Best Blast version and command is now added. The genome accessions

for annotated genes used in this analysis are provided in Table S1.

6. Line 303: S. Merianae genome is used as a source of truth. However, that genome is fragmented as well with 4512 scaffolds. Therefore, the statement in the next line "For example, in S. merianae, three microchromosome account for this scaffold" cannot be robust. This leads to the fact that lines 306-308 cannot be trusted. If authors insist on splitting automatically generated scaffolds using manual curation, then the curation should be applied consistently across the genome and not at handpicked locations. This causes confusion for downstream use of the genome reference.

*** Our assembly results provided 5294 scaffolds but 16 of them were substantially longer (>8 Mbp), while the rest were 8 kbp or shorter. These patterns emerge in chromosome-level assemblies even though the total number of scaffolds can still number in the thousands, as most data will be assembled into a small number of very large scaffolds that approximately represent a chromosome while a small subset of data will remain largely unassembled in very short scaffolds/contigs. Therefore, scaffold number alone does not necessarily indicate that a genome assembly is more or less fragmented. Also, we only conduct manual curation when the karyotype indicates that the observed number of chromosome-length scaffolds is incorrect since these karyotype data are robust and more reliable than in silico assembly. So, we first used chromosomal gene markers (table S1) to 1) identify chromosomes still apparently split across two or more scaffolds and 2) identify chromosomes where we should split sequences because the assembler incorrectly assembled these together. As a result, 6 macrochromosomes were clearly identified (assigned to 7 scaffolds). In microchromosomes, we observed patterns that indicated that the assembler incorrectly assembled multiple microchromosomes together. For these scaffolds, we used a previously published “full evidence” approach to manually identify breakpoints to split these microchromsomes, which is now described in detail in the revised manuscript (lines 351-368).

7. Line 310: How was this performed?

*** To assign 10 scaffolds to 11 microchromosomes, we used several sources of information. First, chromosomal gene markers from Anolis carolinensis identified 4 of our original scaffolds as microchromosomes. One of these microchromosome scaffolds was noticeably larger than the others and preliminary synteny analyses indicated that this large microchromosome matched several distinct microchromosomes in all other species with microchomosomes used in our analysis (an example based on Salvator merianae is provided in the text). These two sources of information strongly suggested that multiple microchromosomes had been incorrectly scaffolded together due to Hi-C data, which has been observed in at least one previous study (Schield et al. 2019). Given this, we used the approach of Schield et al. (2019) to identify candidate breakpoints between microchomosome scaffolds using our Chicago data. For each candidate breakpoint, we used a “full evidence” approach to assess local measures of GC content, repeat density, and gene density to determine whether the scaffold should be split at this breakpoint. This approach allowed us to reliably split the large microchromosome scaffold into two distinct microchromosome scaffolds, which results in a microchromosome count that aligns with karyotypic data. We now clarify this method in greater detail in the manuscript (lines 351-368).

8. Line 316: Replace "unknown" with "novel".

*** We disagree with this point and have not made this edit because the accepted convention is to call these un-classified repeats “unknown” based on how RepeatModeler outputs are structured; this convention follows multiple previous genomes that have been published. Novel suggests that repeats are ‘new to science’, which has not been rigorously evaluated and is not the intended meaning.

9. Line 349: The RBB pipeline is nowhere described at the link provided as reference. The link only provides information about how to create annotation tracks. Please detail methods clearly.

*** We now provide a link for RBB that is called “orthorbb” (line 410).

10. Gene ontology section: Needs more detail about the software version, parameters, commands, and essential thresholds used to determine significance of enrichment or depletion.

*** Software version was added (line 415). Although the analysis is replaced by pathway analysis, the software is same as the previous one (PANTHER). Also, this software is browser-based and there is no parameters or command line that we used since we used their protocol for “gene list” using our genome annotation results. More details are added (lines 416-418)

11. Line 361-362: Please describe the command used to calculate GC content, gene

density, repeat elements etc. What were the sources of these annotations to be used with markwindows tool?
*** More details are added now (lines 424-430). Also, the script for GC content has been already published so we provide a link to it. A script for calculating repeat and gene density has been submitted to GigaDB that is called “window_quantify.py”.
12. Line 363 - 367: Please list assembly version for posterity.
***Done (lines 434-438).
13. Line 368: reference for painting method appears to be incorrect. Please provide accurate reference for the in-silico painting method.
***The reference is correct, but the first author is not the person who has done the synteny analysis and wrote the painting script. We took out the name of the author for synteny script from the text to avoid the confusion (line 439).
14. Painting method requires substantial addition in how the BLAST was performed. What was the tool (blastn, megablast, dcmegablast) used? If default parameters used, then say so.
***Done (line 441-445).

References:
1. Page, issue and volume numbers are present in some but not all references.
*** We have edited the references to be consistent with only page and volume information in the revised draft.
2. Remove letters next to the year. Perhaps something to do with the reference manager.
***Done.
3. Reference 2, 11, 14, 20, 54 are examples of references without complete list of authors. Use consistent style.
***Done.
4. Reference 18 has a typo for the species name. Please correct it.
***Done (reference 20).

Reviewer #2:
This is a review of the manuscript entitled "A chromosome-level genome assembly and annotation of the Desert Horned Lizard, Phrynosoma platyrhinos, provides insight into chromosomal rearrangements among reptiles". This manuscript presents the genome assembly and annotation of the desert horned lizard. Besides providing these resources the authors conduct some analyses that bring insight into micro and macrochromosime evolution. They indicate that gene density seems higher on the microchromosomes, and microchromosomes are more conserved as blocks based on the synteny analysis. The use of an ecological statistics is a clever way for assessing chromosomal dispersion. Overall I think this manuscript a very useful contribution to the field, however, a number of areas in this manuscript need to be clarified and perhaps reevaluated, as described below. I hope the authors find these useful for improving their manuscript.

Major Points:
1. In general, the methods section needs more details, and since the Analysis section is presented first it needs to be sufficient for understanding how you arrived at your findings without having read the methods that are at the end of the manuscript. In many sections I had to read the Methods section alongside the Analysis section to understand the Analysis section.
***We added brief methods to each section in analysis and more details are added in each section of methods.
2. Page 13, methods for breaking scaffold 8.
a. Can you further explain or provide references for why high GC, and low repeats would indicate a good break point. This is not intuitive in the context of telomeres at the end of chromosomes having high repeats of AT rich regions.
*** The reference for indicating the break point for misassembled microchromosomes scaffold is “The origins and evolution of chromosomes, dosage compensation, and mechanisms underlying venom regulation in snakes, Schield and et. al, 2019”. In their Dovetail/HiRise genome assembly they had one large scaffold that contained all microchromosomes, incorrectly merged. In this publication, the authors designed an approach for identifying putative chromosome-breaks within a Hi-C-based scaffold by

Powered by Editorial Manager® and ProduXion Manager® from Aries Systems Corporation

comparing this scaffold with chicken and Anole microchromosomes, together with comparisons of breakpoints between Chicago scaffolds (which cannot span multiple chromosomes). Then, synteny analysis was used to see if any breaks between synteny blocks are matched with Chicago scaffolds breakpoints. Finally, patterns of GC and repeat content were aligned to the overlapping evidence from synteny and Chicago scaffold breakpoints. Their results show that the breakpoints are matched with the shifts to higher GC content, lower repeat element density, and lower gene density, which were also observed near the ends of macrochromosomes in that analysis and in our analyses presented here. We followed this methodology to determine the putative breakpoint between the two P. platyrhinos microchromosomes, which we believe were also incorrectly over-assembled by the same Dovetail/HiRise assembly biases when it comes to Hi-C interpretation from microchromosomes.

b. For transparency, indicate on Figure 2 and Table 2 which microchromosomes derived from the scaffold 8 that was "broken".

*** We now provide this in Table S1. In Fig. 2, we now only show chromosomes as suggested by the reviewers.

c. Indicate on Supplemental Figure 1 where the scaffold was broken and label the ends with their respective microchromosome designations.

*** Done (Figure S3).

d. Why do you think these were put into the same scaffold? This information could be useful for others in trying to understand their assemblies

*** Based on previous studies (Perry and et. al. 2020, and Schield and et. al. 2019) microchromosomes may have a greater frequency of inter-chromosomal contact than expected in models used to scaffold based on Hi-C sequencing data (lines 356-359), thus introducing ‘overassembly’ of chromosomes into scaffolds. We clarified this in the methods for breaking this scaffold, and both of these cited papers describe this in further detail.

3. Scaffolds or Chromosomes: In Figure 2 it is confusing that the two scaffolds you think are chromosome 3 are separated, but scaffold 8 has been broken into the proposed macrochromosomes. I suggest you either use this figure to represent scaffolds with scaffolds for proposed chromosome 3a and 3b separated and the full scaffold 8 intact, OR you use this figure to represent proposed chromosomes with the two scaffolds representing proposed chromosome 3 together and the scaffold 8 broken to represent the proposed microcromosomes. Regardless, for transparency you should have both scaffold and chromosome labels around the circle.

***We chose to have a chromosome level figure instead of scaffolds. The two scaffolds for chromosome 3 are attached together (called chromosome 3 instead of 3a and 3b). Microchromosomes are also presented as the final order. But we believe adding the scaffolds names to it makes it confusing. Also, we have Table S2 that relates all chromosomes to their reference scaffolds.

4. Gene Ontology. I think this is interesting but more details are needed on the GO Function analysis, and I suggest backing off on some conclusions or putting them in the context of the limitations of the study. For example, in the Annotation section it is mentioned that 20,764 protein coding genes were annotated, but in the gene ontology only ~11,000 (~1/2 were used). Why is this the case? Are they predicted proteins without gene "names". And then further only 7000 (1/3 of the annotated protein coding genes) were able to be assigned a molecular function.

*** Among all the genes we annotated in P. platyrhinos genome, about 16,000 were identified with homology-base analysis because we cannot always attach functional information to a gene model based on homology or other options, so these gene models remain “unknown” and cannot be used in downstream analyses of any kind. Among these identified gene IDs, 12,719 genes were identified by PANTHER (considering that repetitive genes on each chromosome count as one, 1,471 gene IDs in total were not identified). We added stacked bar charts (Fig. S2) to visualize these numbers which shows about 90% of annotated genes were identifiable on each group of chromosomes. We also updated our analysis with pathway that includes groups of genes involved in a specific functional group. We believe this analysis is more straightforward along with further explanation.

a. If you are only able to use 1/3 of the annotated genes in your analysis, how confident are you in these results when most of the data are missing?

*** We mentioned in the conclusion that these results are preliminary and require further investigation (lines 274-276).

b. Is this 1/3 of genes that can be included in your analytical tests evenly distributed

among the chromosomes? For example, are 1/3 of the annotated genes on chromosome 1 included in your test, and are 1/3 of the annotated genes on microchromosome 3 included in your test? OR are these proportions very different across the chromosomes? If they are different what bias does that introduce in this test?
*** We estimated how many genes are annotated with pathway analysis on each individual chromosome and visualized it in Fig. S2 which showed the proportion of annotated genes are roughly the same on each chromosome and more than 80% of each chromosome genes are included in the analysis.
c. What statistical model was used for testing for different molecular functions associated with the micro vs macrochromosomes? How many tests were completed: 8 level 1, 42 level 2, 142 level 3 = 192 statistical tests? Was a false discovery rate used in determining statistical significance?
***Molecular function analysis is replaced with pathway analysis and absent/present of the pathways is compared.
d. Table S3 needs to have the adjusted P-values or FDR for statistical significance included as a column.
***This table is replaced with a summary table for new functional analysis.
e. Clarify how the "activating / positive regulatory" and "repressive/negative regulatory" roles are being defined?
***This section is deleted because of an updated analysis.
5. Page 6, Synteny analysis. It seems circular to say A. carolinensis had the same macrochromsome structure when you used genes from A. carolinensis to define the P. platyrhinos macrochromosomes (including the 3a and 3b). If you disagree, please explain to me and other readers why this would not be the case.
***The gene markers for chromosome identification are based on a limited number of genes (25 from A. carolinensis) for all chromosomes (Table S2) but for synteny analysis we aligned both genome assemblies against each other and got more than 57,000 markers for A. carolinensis (Table S5) that were used in this analysis, which is comparing chromosomes in terms of “synteny blocks”. So, this analysis is based on conservative blocks of sequences, not names of genes.
6. Metrics for quality of the assembly are needed. BUSCOs were run (in the online data) but the results not reported in the manuscript. Many BUSCOs are missing C:46.7%[S:46.2%,D:0.5%],F:7.2%,M:46.1%,n:5310
This is much more than I would expect if this is a high quality chromosome-level assembly. Why do you think this is? Was this run on the complete assembly or only the "chromosome" scaffolds? Include a description in the text of this BUSCO analysis and include a summary table of the BUSCO results. If there are other metrics you could use to further understand the quality of this assembly it would be encouraged.
***A BUSCO table was added to the manuscript (Table 2). The run was performed using the entire genome. We also updated the results in this revision of the manuscript (C:68.6%[S:68.0%,D:0.6%],F:12.9%,M:18.5%,n:5310). Using other metrics (line 334) such as contig N50 shows our data are short reads (relatively short N50), so we believe that gaps/misassemblies lead to missing BUSCOs.

Finer points.
1. Page 13: In methods define the best blast parameters.
***We added a link to the script was used (line 410).
2. Synteny Figures: the phylogeny lines are very faint and didn't show up on a print out
*** We edited the lines.
3. Table 1: Percent of genome in gaps for Chicago + Hi-C assembly is missing. It is interesting there are MORE gaps in the Chicago + Hi-C Assembly. Why is this?
***The percentage of gaps is added now. In general, we expected to see more gaps in the Chicago + Hi-C assembly than the Chicago or original assemblies alone, as the Hi-C data is used to scaffold contigs/scaffolds more. Whenever scaffolding occurs, gaps will be introduced, and the software cannot always fill these gaps.
4. Table 2, it seems this information could easily be incorporated into Figure 2.
*** We combined this table with the table the reviewer asked for in comment 8 (corresponding scaffolds; Table S1).
5. Table S1 could use a much better description. Is chromosome relative to Anole and Scaffold relative to P. platyrhinos? You have chromosome names rather than scaffold names, this is confusing because the terms are not interchangeable,

Powered by Editorial Manager® and ProduXion Manager® from Aries Systems Corporation

	especially when you are combining and splitting scaffolds to "define" the proposed chromosomes.<br>***We edited the chromosome column and added the species name (Table S2).<br>6. Figure 3. Why are the chromosomes in some species not sorted by size, when they seem to be in all the other species - is this meaning full in some way?<br>*** We used the order that the chromosomes were sorted in their assemblies, and we believe this way is easier for the readers to follow and check with Genbank if they are interested in a specific chromosome.<br>7. Indicate that you concatenated the 3a and 3b in the synteny figure legends.<br>*** Done for Figures 2, 3, and S1.<br>8. Describe either in the text or in the readme, the organization of the final assembly. Are the scaffolds organized by size? What are the scaffold names that correspond to each chromosome (this could be included a table or a figure). If the scaffold containing the two microscrosomes is still intact as a single scaffold, report the point at which you think it should be broken. That will be useful information for anyone wanting to use your assembly.<br>***The scaffolds are organized based on their scaffold name/number (described in the readme). A table was added to the supplemental tables (Table S1) that shows corresponding scaffolds for each chromosome. We already broke down the scaffold and more details for breaking this scaffold is added (lines 353-369, and Fig. S3).<br>9. Be sure to include a description for all the online files in the Read.me<br>*** Done.<br><br>--<br>Please also take a moment to check our website at https://www.editorialmanager.com/giga/l.asp?i=85421&l=PBWIF3YE for any additional comments that were saved as attachments. Please note that as GigaScience has a policy of open peer review, you will be able to see the names of the reviewers.<br><br>____<br>In compliance with data protection regulations, you may request that we remove your personal registration details at any time. (Use the following URL: https://www.editorialmanager.com/giga/login.asp?a=r). Please contact the publication office if you have any questions.
**Additional Information:**	
**Question**	**Response**
Are you submitting this manuscript to a special series or article collection?	No
**Experimental design and statistics**<br><br>Full details of the experimental design and statistical methods used should be given in the Methods section, as detailed in our Minimum Standards Reporting Checklist. Information essential to interpreting the data presented should be made available in the figure legends.<br><br>Have you included all the information requested in your manuscript?	Yes
**Resources**	Yes

A description of all resources used, including antibodies, cell lines, animals and software tools, with enough information to allow them to be uniquely identified, should be included in the Methods section. Authors are strongly encouraged to cite Research Resource Identifiers (RRIDs) for antibodies, model organisms and tools, where possible.<br><br>Have you included the information requested as detailed in our Minimum Standards Reporting Checklist?	
**Availability of data and materials**<br><br>All datasets and code on which the conclusions of the paper rely must be either included in your submission or deposited in publicly available repositories (where available and ethically appropriate), referencing such data using a unique identifier in the references and in the “Availability of Data and Materials” section of your manuscript.<br><br>Have you have met the above requirement as detailed in our Minimum Standards Reporting Checklist?	Yes

Manuscript

Click here to access/download;Manuscript;Phrynosoma_platyrhinos_genome_

A chromosome-level genome assembly and annotation of the desert horned lizard, *Phrynosoma platyrhinos,* provides insight into chromosomal rearrangements among reptiles

Running title: genome of *P. platyrhinos*

Nazila Koochekian (*Correspondence): Department of Biology, Miami University, Oxford, OH, 45056, USA. Email: koochen@miamioh.edu

Alfredo Ascanio: Department of Biology, Miami University, Oxford, OH, 45056, USA. Email: ascaniaa@miamioh.edu

Keaka Farleigh: Department of Biology, Miami University, Oxford, OH, 45056, USA. Email: farleik@miamioh.edu

Daren C. Card: Department of Organismic & Evolutionary Biology, Harvard University, Cambridge, MA, 02138, USA, and Museum of Comparative Zoology, Harvard University, Cambridge, MA, 02138, USA. Email: dcard@fas.harvard.edu

Drew R. Schield: Department of Ecology and Evolutionary Biology, University of Colorado, Boulder, CO, 80309, USA. Email: Drew.Schield@colorado.edu

Todd A. Castoe: Department of Biology, University of Texas at Arlington, Arlington, TX, 76019, USA. Email: todd.castoe@uta.edu

Tereza Jezkova: Department of Biology, Miami University, Oxford, OH, 45056, USA. Email: jezkovt@miamioh.edu

Abstract

Background. The increasing number of chromosome-level genome assemblies has advanced our knowledge and understanding of macroevolutionary processes. Here, we introduce the genome of the desert horned lizard, *Phrynosoma platyrhinos,* an iguanid lizard occupying extreme desert conditions of the American southwest. We conduct analysis of the chromosomal structure and composition of this species and compare these features across genomes of 12 other reptiles (5 species of lizards, 3 snakes, 3 turtles, and 1 bird).

Findings. The desert horned lizard genome was sequenced using Illumina paired-end reads and assembled and scaffolded using Dovetail Genomics Hi-C and Chicago long-range contact data. The resulting genome assembly has a total length of 1,901.85 Mb, scaffold N50 length of 273.213 Mb, and includes 5,294 scaffolds. The chromosome-level assembly is composed of 6 macrochromosomes and 11 microchromosomes. A total of 20,764 genes were annotated in the assembly. GC content and gene density are higher for microchromosomes than macrochromosomes, while repeat element distributions show the opposite trend. Pathway analyses provide preliminary evidence that microchromosome and macrochromosome gene content are functionally distinct. Synteny analysis indicates that large microchromosome blocks are conserved among closely related species, whereas macrochromosomes show evidence of frequent fusion and fission events among reptiles, even between closely related species.

Conclusions: Our results demonstrate dynamic karyotypic evolution across Reptilia, with frequent inferred splits, fusions, and rearrangements that have resulted in shuffling of chromosomal blocks between macrochromosomes and microchromosomes. Our analyses also provide new evidence for distinct gene content and chromosomal structure between microchromosomes and macrochromosomes within reptiles.

Key words: microchromosome; macrochromosome; gene content; synteny; Reptilia

Background

The increasing number of available chromosome-level genome assemblies of non-traditional model organisms has advanced our understanding of genome evolution over large time scales, including intra- and inter-chromosomal rearrangements and karyotype evolution across amniote vertebrates. A major gap in our understanding of amniote genome structure, composition, and evolution has been due to the lack of representative reptilian genomes of high enough quality to compare chromosome composition and structure. From data that is available, reptiles (the clade of Sauropsida) appear to exhibit particularly high levels of karyotypic variation (Fig. 1)[1, 2]. Much of this karyotypic variation is apparently due to frequent merging, splitting, and rearrangements among chromosomes, resulting in varying numbers and sizes of chromosomes even among closely related taxa (Fig. 1). Unlike mammalian genomes which lack microchromosomes, most reptilian genomes contain both macrochromosomes and microchromosomes [3]. The condition of possessing both macro- and microchromosomes appears to represent an ancient ancestral state that spans 400–450 million years of evolutionary history, as microchromosomes are present in many ancient chordates, fish, and amphibians, and all amniote vertebrates except mammals and crocodilians [3]. Microchromosomes are generally identified by their smaller size (50 Mb threshold in squamates [4]). In the chicken, for example, microchromosomes range from 3.5 to 23 Mb [5], compared to macrochromosomes which range from 40 to 250 Mb [6].

Although microchromosome organization in avian species is relatively conserved at a karyotypic level [7], microchromosomes of non-avian reptiles vary considerably in number and size [8], potentially due to relatively high recombination rates [9] that lead to higher rates of chromosomal rearrangement [3,10]. Despite being a promising system in which to study karyotypic evolution, relatively little is known about the genomic features of macrochromosomes and microchromosomes and how these features evolve across Reptilia [11]. Moreover,

microchromosomes appear structurally and functionally distinct from macrochromosomes [12], and a deeper characterization of these distinctions may improve our understanding of the functional and evolutionary significance of the presence/absence of microchromosomes, and the presence of genes on micro- versus macrochromosomes. Despite interest in the processes and patterns related to chromosome evolution in reptiles, progress has been limited by the availability of relatively few high-quality reptile genomes available for comparative study. In lizards, only five genomes are annotated and assembled at the level of chromosomes (i.e., chromosome-size scaffolds that in many cases have been ascribed to specific chromosomes): the green anole, *Anolis carolinensis* with 6 chromosomes and 7 microchromosomal linkage groups [13], the viviparous lizard, *Zootoca vivipara* with 19 chromosomal linkage groups [14], the sand lizard, *Lacerta agilis* with 18 autosomes and Z and W sex chromosomes [15], the common wall lizard, *Podarcis muralis* with 18 autosomes and a Z sex chromosome [16], and the Argentine black and white tegu, *Salvator merianae*, with chromosome-scale scaffolds that have not been fully ascribed to specific chromosomes [17].

Here we present a new chromosome-level genome assembly of the desert horned lizard (*P. platyrhinos*) and use this genome to conduct comparative analysis of chromosome content and evolution across reptiles. This species is widely distributed across the southwestern deserts of north America, including some of the hottest and driest places on Earth (e.g. Death valley in the Mojave Desert; [18]) which makes it an attractive model organism to study adaptation to extreme thermal environments. We have annotated the genome assembly and assessed large-scale structure and composition of the genome across macrochromosomes and microchromosomes. Using this new resource, we conduct synteny analyses to explore major changes in genome organization by making comparisons with existing chromosome-level annotated genomes of other lizards (*A. carolinensis, S. merianae, L. agilis, Z. vivipara* and *P. muralis*), snakes (*Crotalus viridis* [19], *Thamnophis elegans* [20], and *Naja naja* [21]), a bird (*Gallus gallus* [22]), and turtles (*Trachemys scripta* [23], *Gopherus evgoodei* [24], and

Dermochelys coriacea [25]). Our findings reveal differences in structure and gene content of macrochromosomes and microchromosomes in *P. platyrhinos* and highlight numerous chromosomal rearrangements among reptiles.

Analysis

Genome assembly, transcriptome assembly, and chromosome identification

The genome of *P. platyrhinos* was sequenced at 21,053.74-fold physical coverage using the Dovetail Genomics HiRise™ [26] sequencing and assembly approach that combines a contig-level assembly produced from shotgun Illumina sequencing with long-range scaffolding data from Chicago and Hi-C library preparations (Table 1). The final assembly included 5,294 total scaffolds, with 7 large scaffolds and 10 smaller scaffolds comprising 99.56% of the genome assembly. The known karyotype of the species is composed of 6 macrochromosomes and 11 microchromosomes [27,28] and we assumed this karyotype when linking chromosomes to their representative assembly scaffolds. Using chromosome-linked gene markers from *A. carolinensis* and *Leiolepis reevesii* [29], the 7 largest scaffolds were assigned to macrochromosomes 1-6 (two scaffolds corresponded to the two arms of macrochromosome 3; Table S1 and Table S2). Ten smaller scaffolds were assigned to microchromosomes, and one of these scaffolds was manually split into two microchromosomes (Table S1). We followed previous studies [8] to infer the location of the putative split between chromosomes by combining evidence from physically-linked Chicago scaffolds that cannot span multiple chromosomes, repeat element and GC composition, and synteny with chromosomes of other species (see Methods).

The chromosome-linked gene markers used to identify chromosome scaffolds do not identify specific microchromosome numbers (Table S2), so we ordered the assembled *P. platyrhinos* microchromosomes by descending length and numbered them microchromosomes

1-11 (Table S1). Sex chromosomes are conserved across iguanid lizards [30] and we identified microchromosome 9 as the X chromosome in *P. platyrhinos* based on homology with X-linked markers in *A. carolinensis* (*ATP2A2*, *FZD10*, and *TMEM132D* [30]; Table S2).

RNA-sequencing of 8 tissues (liver, lungs, brain, muscle, testes, heart, eyes, and kidneys) was used to assemble the transcriptome of *P. platyrhinos* using Trinity r2014 0413p1 [31]. The final transcriptome assembly contained 199,541 transcripts comprising 199,500 Trinity-annotated genes, with an average length of 1,438 base pairs and an N50 length of 2,420 bp.

Genome annotation and chromosomal composition

We annotated 20,764 protein-coding genes in the *P. platyrhinos* genome assembly (JAIPUX010000000) using the gene prediction software MAKER v. 2.31.10 [32] and gene predictions based on AUGUSTUS v. 3.2.3. [33]. Among the total annotated genes, 16,384 genes were identified using searches against protein sequences in databases NCBI and Interpro [34]. We identified 4,324 complete and fragmented BUSCO markers in the *P. platyrhinos* genome annotation from the total 5,310 BUSCO markers present in the library "tetrapoda_odb10.2019-11-20" (Table 2). Our repeat annotation identified 44.45% of the genome as repetitive elements (Table S3) using RepeatModeler v. 1.0.11 [35] and RepeatMasker v. 4.0.8 [36]. The major components of the genomic repeat content included simple sequence repeats (6.90%), as well as L2/CR1/Rex (6.88%), hobo-Activator (5.98%), and Tourist/Harbinger (4.90%) transposable element families (Table S3).

Chromosomal composition analyses indicate that overall gene density (GD) and GC-content tended to be lower on *P. platyrhinos* macrochromosomes (mean ± sd GD = 0.19 ± 0.14, median = 0.17 per Mb; mean ± sd GC% = 35.9 ± 1.2%, median = 35.9%) than microchromosomes (mean ± sd GD = 0.27 ± 0.16, median = 0.29 per Mb; mean ± sd GC% = 38.5 ± 2.8%, median = 38.2%; Fig. 2 and S1). Conversely, repeat elements density tended to

be higher on macrochromosomes (mean ± sd = 44.6 ± 5.6%, median = 43.3% per Mb) than microchromosomes (mean = 39.4 ± 10%, median = 38.1% per Mb; Fig. 2 and S1). These differences in GD, GC-content, and repeat elements between macro and microchromosomes were statistically significant (Wilcoxon-W = 137011, p-value = $5.7*10^{-16}$ for GD; Wilcoxon-W = 68322, p-value < $2.2*10^{-16}$ for GC-content; and Wilcoxon-W = 283330, p-value < $2.2*10^{-16}$ for repeat elements).

Pathway analysis

We assessed whether macrochromosomes and microchromosomes contain distinct functional classes of genes using pathway analyses. From the total of 16,384 protein coding genes that were identified by homology search, 9,590 gene IDs on macrochromosomes and 3,129 on microchromosomes were identifiable by PANTHER16.0 [37,38] using the protein family/subfamily library (Fig. S2). These genes were classified into a total of 164 pathways from about 177 available pathways in PANTHER. The highest number of genes belonged to the "Wnt signaling pathway (P00057)" and "Gonadotropin-releasing hormone receptor pathway (P06664)", which together accounted for more than 10% (more than 5% each) of the macrochromosomal and microchromosomal genes. We compared the frequencies of genes in each PANTHER pathway between macrochromosomes and microchromosomes and found 37 pathways where all genes were located on macrochromosomes (Table S4), with 13 pathways having all genes localized to a single macrochromosome. Among microchromosomes, we found that three pathways have genes exclusively found on only microchromosomes and in all three pathways, these genes were located on a single microchromosome (Table S4). These 40 pathways (37 for macrochromosomes + 3 for microchromosomes) mostly belong to biosynthesis, signaling, metabolism, and degradation pathways (in descending order).

Synteny analysis

We investigated how reptilian genome composition has been impacted by chromosomal rearrangements through evolutionary time using comparative synteny analyses among reptiles. We conducted pairwise analyses of synteny between the *P. platyrhinos* genome and 12 species (five lizards, three snakes, three turtles, and a bird) for which chromosome-level genome assemblies were available (Fig. 3)[39]. The genome of *S. merianae* has not been assembled to chromosomes but the karyotype of this species is known (5 macrochromosome and 14 microchromosomes; [40]) so in this study we used 19 largest scaffolds from the *S. merianae* assembly with 5 scaffolds > 200 Mb and 14 scaffolds > 6 Mb). We performed synteny analyses using a 'chromosome painting' technique (see Methods), which established homology between sets of 100 bp *in silico* 'markers' from the *P. platyrhinos* chromosome scaffolds and regions of the genomes of the other reptile species (Table S5). We quantitatively assessed the degree to which syntenic blocks from each *P. platyrhinos* chromosome scaffold are dispersed across chromosomes of the other species (Fig. 4) using a dominance analysis [41], more commonly used in ecological community assessments. Specifically, dispersion was measured using the Simpson's Dominance Index reciprocal (SR), with which we consider an effective number of target chromosomes in other species onto which the homologies of a given *P. platyrhinos* chromosome appear. This index ranges from 1 to *m*, where *m* is the number of chromosomes of the target species being compared to *P. platyrhinos*. A value of 1 represents high dominance, which in this context indicates that syntenic blocks from a chromosome of *P. platyrhinos* are restricted to a single chromosome of another species. A value of *m* would mean all chromosomes of the target species contain an even proportion of *P. platyrhinos* syntenic blocks. If a large syntenic block is retained in one chromosome while a few proportionally small syntenic blocks are distributed across other target chromosomes, the resulting dominance value will trend toward 1.

Our results show that macrochromosomes tend to have a higher degree of dispersion across different chromosomes of other species than microchromosomes (e.g., macrochromosome 1 SR = 2.38 ± 0.96; microchromosome 1 SR = 1.45 ± 0.45), except for macrochromosome 6 (SR = 1.44 ± 0.27; Fig. 5a). However, this chromosomal rearrangement does not follow the same pattern across species (Fig. 4). For example, *A. carolinensis* shows the highest values for SR in microchromosomes (Fig. 5b), but this may be an artifact of this species having an incomplete genome assembly for microchromosomes. In other lizards and snakes (with the exception of *C. viridis*), SR ~ 1 for all microchromosomes (except microchromosome 6). In *G. gallus*, SR ~ 1 for all microchromosomes except microchromosome 1. In turtles, mean SR values for microchromosomes are > 1, but this is largely driven by higher SR values on microchromosomes 1, 4, and 6 (Fig. 4).

Macrochromosome synteny appears highly conserved between *P. platyrhinos* and *S. merianae*. Among the closest relatives of *P. platyrhinos*, *A. carolinensis* has the same macrochromosome arrangement as *P. platyrhinos* (Figs. 3-5). In the more distantly related snakes, *N. naja* and *C. viridis*, however, macrochromosomes 3 and 5 show high SR values and the remaining macrochromosomes have SR ~ 1. Compared to the other snakes, *T. elegans* (along with lizards in the family Lacertidae) generally possess a greater number of smaller macrochromosomes than *P. platyrhinos* and associated higher SR values. At greater phylogenetic distances, the breakdown of chromosomal synteny from lizards to other reptilian lineages becomes more apparent (cumulative SR ~ 30 in turtles) and showing greater rearrangements and partitions of syntenic blocks in macrochromosomes than in microchromosomes (Fig. 4 and 5b).

Our results also show that rearrangements between macro- and microchromosomes are apparently common throughout the evolution of Reptilia, including macro and microchromosomes fusing together to form single macrochromosomes. For example, microchromosomes 5 and 6 in *P. platyrhinos* form a macrochromosome in *L. agilis, Z. vivipara*,

and *P. muralis*, chromosome 6 of *P. platyrhinos* is syntenic with a macrochromosome and a microchromosome in *S. merianae,* and microchromosome 6 of *P. platyrhinos* comprises two microchromosomes in *S. merianae*, *G. gallus,* and turtle species (Fig. 3).

Discussion

The *P. platyrhinos* genome is only the second chromosome-level assembly available for the diverse lizard family Iguanidae (after *A. carolinensis*), and the only member of this family with well assembled microchromosomes, thereby contributing a new valuable resource for comparative genomics of reptiles. For *P. platyrhinos*, we identified scaffolds representing the 6 macrochromosomes and 11 microchromosomes that comprise the known karyotype for the genus *Phrynosoma* [27,28,42]. The higher contiguity and completeness of microchromosomal scaffolds in the *P. platyrhinos* genome relative to that of *A. carolinensis* enables some of the first comparisons of chromosome evolution in lizards that incorporates patterns distinct to macro- versus microchromosomes. Our analyses of this and other comparative reptilian genomes highlight distinct functional classes of genes, chromosomal structure, and rearrangement patterns in microchromosomes compared to macrochromosomes.

Consistent with previous studies of reptilian chromosome composition [8,9,43], we find that in *P. platyrhinos*, GC content, gene density, and repeat element density differ between macrochromosomes and microchromosomes, with gene density and GC content being higher on microchromosomes and repeat elements being more densely distributed on macrochromosomes. Patterns of high gene density on microchromosomes have been hypothesized to be an evolutionary solution to reduce overall DNA mass and increase recombination rates between coding regions, predominantly by reducing repeat element content [3]. High recombination rates further increase GC content due to GC-biased gene conversion [44], leading to a higher frequency of GC bases on microchromosomes that can house

functionally different gene content compared to macrochromosomes [12], a pattern we also observed in the *P. platyrhinos* genome (Fig. 2 and S1).

Our synteny analyses across reptile genomes revealed that splitting, fusion, and rearrangement events among chromosomes have occurred frequently and repeatedly throughout reptile evolution. This pattern of chromosome blocks shifting between macro-, and microchromosome-linkage likely explains some unusual patterns of gene density, GC-content, and repeat elements, such as blocks of high gene density on a macrochromosome that may represent ancestral fragments derived from microchromosomes. For example, high GC content and gene density relative to other macrochromosomes on one end of macrochromosome 6 of *P. platyrhinos* (extending for ~40 Mbp; Fig. 2) supports the scenario that a microchromosomal region with higher gene and GC density was recently translocated to a macrochromosome in the ancestor of *P. platyrhinos*. This process may have also contributed to the observed variation in the numbers and sizes of macro- and microchromosomes, even among closely related species (e.g., *P. platyrhinos* versus *A. carolinensis*, and *C. viridis* versus *T. elegans*). Among macrochromosomes, fusion, splitting, and translocation to other chromosomes in more distantly related species such as turtles and chicken are common, whereas microchromosomes of *P. platyrhinos* typically remain in single homologous blocks in these other reptilian lineages, though there are exceptions (Fig. 4 and Fig. 5b). Broadly, these findings suggest that ancestral chromosomal rearrangements may have resulted in regions of reptilian genomes that have not yet reached mutational and compositional equilibria, which are otherwise characteristic of macro- and microchromosomal regions, following ancestral chromosomal rearrangement events.

Adding to the growing body of evidence for the structural, compositional, and evolutionary distinctions between micro- and macrochromosomes [9,12,45,46,47,48,49], our analyses suggest that the gene content of these two classes of chromosomes may be distinct in function. Our preliminary observation of enrichment of genes from certain pathways on

individual chromosomes or on macro- and microchromosomes more generally warrants further investigation. These biases could be driven by ancestral contingencies of gene content or active translocations of genes across chromosome classes, which may suggest a functionally driven basis for such biases. These inferences, together with other emerging evidence for the compositional and functional distinctiveness between micro- and macrochromosomes [9,12,45] suggest that there may be key functional, evolutionary, and mechanistic features that distinguish these chromosome classes that explain the significance of the presence and abundance of microchromosomes across eukaryote lineages.

Methods

Genome and transcriptome assembly

We sequenced and assembled the reference genome from a female desert horned lizard collected in Dry Lake Valley, Nevada (NCBI accession SAMN17187150). This specimen was collected and euthanized according to Miami University Institutional Animal Care and Use Committee protocol 992_2021_Apr. Liver tissue was snap frozen in liquid nitrogen and sent to Dovetail Genomics (Scotts Valley, CL) for extraction of DNA and construction of shotgun, Chicago, and Dovetail Hi-C paired end libraries. DNA was extracted using buffer G2, and Qiagen protease. Three initial shotgun sequencing libraries were constructed by fragmenting DNA extracts to 475 bp and using a TruSeq PCR-free library prep kit to ligate sequencing adapters and amplify each library. The resulting libraries were sequenced on an Illumina HiSeqX and resulted in 859.9 million read pairs from paired end libraries (totaling 246 Gbp; see Table 3 for the number of sequenced reads for each library). Reads were trimmed for quality, sequencing adapters, and mate pair adapters using Trimmomatic [50], Using these data, contigs and small

scaffolds were assembled using Meraculous 2.2.4 (diploid_mode 1) [51] with a kmer size of 49-mers. which produced an assembly with a scaffold N50 of 0.013 Mb.

The original assembly was first scaffolded using a Chicago library according to the manufacturer's protocol. Three Chicago libraries were prepared as described previously [26]. Briefly, for each library, ~500ng of HMW gDNA was reconstituted into chromatin *in vitro* and fixed with formaldehyde. Fixed chromatin was digested with DpnII, the 5' overhangs filled in with biotinylated nucleotides, and then free blunt ends were ligated. After ligation, crosslinks were reversed, and the DNA purified from protein. Purified DNA was treated to remove biotin that was not internal to ligated fragments. The DNA was then sheared to ~350 bp mean fragment size and sequencing libraries were generated using NEBNext Ultra enzymes and Illumina-compatible adapters. Biotin-containing fragments were isolated using streptavidin beads before PCR enrichment of each library. The libraries were sequenced on an Illumina HiSeqX. The number and length of read pairs produced for all libraries was 528 million 2x150 bp paired end reads (see Table 3 for the number of sequenced reads for each library). The resulting scaffolded assembly was far more contiguous with a scaffold N50 of 63.431 Mb. Lastly, a final round of scaffolding was performed using data from the Dovetail Hi-C library according to the manufacturer's protocols. Three Dovetail Hi-C libraries were prepared in a similar manner as described previously [50]. Briefly, for each library, chromatin was fixed in place with formaldehyde in the nucleus and then extracted. The following steps were the same as creating Chicago libraries. The number and length of read pairs produced for all libraries was 515 million 2x150 bp paired end reads (see Table 3 for the number of sequenced reads for each library). The input *de novo* assembly, Chicago library reads, and Dovetail Hi-C library reads were used as input data for HiRise, a software pipeline (https://github.com/DovetailGenomics/HiRise_July2015_GR) designed specifically for using proximity ligation data to scaffold genome assemblies. First, Chicago library sequences were aligned to the draft input assembly using SNAP v1.0.0 [51] (http://snap.cs.berkeley.edu). The separations of Chicago read pairs mapped within draft scaffolds were analyzed by HiRise to

produce a likelihood model for genomic distance between read pairs, and the model was used to identify and break putative misjoins, to score prospective joins, and make joins above a threshold. After aligning and scaffolding Chicago data, Dovetail Hi-C library sequences were aligned and scaffolded following the same method. The final assembly (NCBI accession PRJNA685451) has a length of 1,901.85 Mb with a contig N50 of 12.04 kb and a scaffold N50 of 273.213 Mb (see Table 1 for more statistics for this genome assembly).

Transcriptomic libraries were sequenced from 8 tissues (liver, lungs, brain, muscle, testes, heart, eyes, and kidneys) from a male lizard collected and euthanized according to Miami University Institutional Animal Care and Use Committee protocol 992_2021_Apr at the same locality as the genome animal. For each library, total RNA was extracted using Trizol reagent, and unstranded mRNAseq libraries were individually prepared using an NEBNext Ultra RNA Library Prep kit with library insert sizes of 250-300 bp and sequenced on an Illumina Hiseq4000 platform using a paired-end 150 bp run by Novogene Corporation Inc (Table 4). We used Trinity r2014 0413p1 to assemble transcriptome reads from all tissues (using min_kmer_cov:1 and default settings).

Chromosome identification

According to the karyotype for phrynosomatid [42] and *P. platyrhinos* [27,52] (2n=34), we expected 6 pairs of macrochromosomes and 11 pairs of microchromosomes (one pair of microchromosomes is expected to be sex linked) for *P. platyrhinos*, and assumed this karyotype was correct for organizing our scaffolded genome assembly. Assigning scaffolds to specific chromosomes was done using blast+2.8.0 [53] using program “blastx” (options “num_threads”=4, “-max_target_seqs”=10, “-evalue”= 1e-5, and “-outfmt”=11). We used chromosome-linked gene markers in other close species (*A. carolinensis*, *Leiolepis reevesii*) [29] and X-linked markers in *A. carolinensis* [39] downloaded from NCBI (Table S1) to identify the genomic location of each gene marker. Available markers for macrochromosomes in lizards

were matched to seven of the largest scaffolds (two scaffolds for chromosome 3), which we sorted by size and named macrochromosomes 1-6. From the remaining scaffolds, 10 scaffolds (> 8 Mbp) were selected as potential microchromosomes. This suggested that one scaffold comprises two microchromosomes fused together as the expected number of microchromosomes was 11. Synteny analysis suggested that scaffold "Scf4326_4427" (Fig. 6) has at least three origins in other closely related species. For example, in *S. merianae,* three microchromosome account for this scaffold, while the rest of scaffolds were linked to a specific microchromosome. Given that Chicago libraries reconstitute chromatin *in vitro*, interactions between distinct chromosomes are significantly reduced compared to *in vivo* Hi-C libraries [54]. Also, microchromosomes may have a greater frequency of inter-chromosomal contact [12] than expected in models used to scaffold based on Hi-C sequencing data. Therefore, we scanned for breakpoints between Chicago scaffolds in microchromosome scaffolds and for each of these breakpoints, we used multiple forms of evidence to assess whether a scaffold should be manually split. Following Schield [8], patterns of GC content, repeat density, and gene density at each breakpoint were assessed and we looked for instances in which there were abrupt shifts in these measures near breakpoints between Chicago scaffolds. At two of these breakpoints on the putatively artificially-merged (with a window of about 100 bp Ns/gaps) scaffold "Scf4326_4427", we observed elevated GC content, and reduced repeat elements density (Fig. S3). Based on these patterns, we chose to split this scaffold at the breakpoint location with reduced gene density to produce a final, curated assembly with the expected number of microchromosomes and finally numbered them based on their size.

Genome annotation

Repeat elements were first identified using RepeatModeler v. 1.0.11 [35] for *de novo* prediction of repeat families. To annotate genome-wide complex repeats, we used RepeatMasker v. 4.0.8 [36] with default settings to identify known Tetrapoda repeats present in the curated Repbase

database release 20181026 [55]. We then ran 2 iterative rounds of RepeatMasker to annotate the known and the unknown elements identified by RepeatModeler, respectively, where the genome sequence provided for each analysis was masked based on all previous rounds of RepeatMasker.

We used MAKER v. 2.31.10 [32] as a consensus-based approach to annotate protein-coding genes in an iterative fashion. For annotation, a genome with complex, interspersed repeats hard masked as Ns was supplied and we set the ‘model_org’ option to ‘simple’ in the MAKER control file (maker_opts.ctl) to have MAKER soft mask simple repeats prior to gene annotation. The full *de novo P. platyrhinos* transcriptome assembly and protein datasets consisting of all annotated proteins for *A. carolinensis* [13] from NCBI were used as the evidence for protein coding gene prediction. For the first round of annotation, “est2genome” and “protein2genome” were set to 1 to predict genes based on the aligned transcripts and proteins. Using the gene models from the first round of MAKER, we were able to train gene prediction software AUGUSTUS v. 3.2.3. [33]. To do so, we used Benchmarking Universal Single-Copy Orthologs (BUSCOs) v. 2.0.1, which has an internal pipeline to automate the training of Augustus based on a set of conserved, single-copy orthologs for Tetrapoda (Tetrapoda odb9 dataset) [56]. We ran BUSCO in the ‘genome’ mode and specified the ‘--long' option to have BUSCO perform internal Augustus parameter optimization. Then we ran MAKER with *ab initio* gene prediction (‘est2genome=0’ and ‘protein2genome=0’ options set) using transcripts, proteins, and repeat elements resulted from the first MAKER round as the empirical evidence (in GFF format) to produce gene models using the AUGUSTUS within the MAKER. For all MAKER analyses, we used default settings, except for ‘trna’ (set to 1), ‘max_dna_len’ (set to 300,000) and ‘split_hit’ (set to 20,000). We used the gene models from our second round of MAKER annotation to re-optimize AUGUSTUS as described above before running one final MAKER analysis (round 3) with the re-optimized AUGUSTUS settings (all other settings are identical to round 2). We compared Annotation Edit Distance (AED) distributions, gene numbers, and average gene lengths across each round of

Maker annotation to assess quality and used our final MAKER round (round 3; N = 20,764 genes) as our final gene annotation.

We ascribed gene IDs based on homology using reciprocal best-blast (with e-value thresholds of 1e-5) and stringent one-way blast (with an e-value threshold of 1e-8) searches against protein sequences from NCBI for *A. carolinensis*, *Pogona vitticeps* [57], *P. muralis* [16], *Gekko Japanese* [58], *Python molurus* [59], *Pseudonaja textilis* [60], *Notechis scutatus* [60], *Protobothrops mucrosquamatus* [61], *Thamnophis sirtalis* [62], *Alligator mississippiensis* [63], *Alligator sinensis* [64], *Crocodylus porosus* [65], *Chrysemys picta* [66], *Terrapene carolina* [67], *Chelonia mydas* [68], *Pelodiscus sinensis* [68], *G. gallus, Homo sapiens* [69], *Mus musculus* [70], and Swiss-Prot [71] using a custom reciprocal best blast (RBB) script (orthorbb 2.2; see https://github.com/darencard/GenomeAnnotation/blob/master/orthorbb). We also searched our annotated transcriptome against Interpro database via Interproscan--5.36-75.0 [72].

Pathway analysis

To compare macrochromosomes and microchromosomes functionally, protein coding genes on each chromosome were analyzed using gene IDs resulted from homology search. An ID list of all annotated genes on each chromosome was used for pathway analysis in PANTHER16.0 (http://pantherdb.org/ via browser and "Gene List Analysis" tools option) classification system. Four model organisms (*A. carolinensis*, *G. gallus, M. musculus,* and *H. sapiens*) were selected as the reference for gene IDs. PANTHER assigned each gene to at least one of the 164 pathways identified for *P. platyrhinos* genome annotation (with a range from 2 to 759 genes in each pathway; Fig. S4). The distributions of each pathway among different chromosomes were compared using pathway results for each chromosome to identify potential pathways that belong to a specific chromosome/group of chromosomes.

Synteny and chromosomal composition

We used a python script “slidingwindow_gc_content.py” (https://github.com/drewschield/Comparative-Genomics-Tools) to estimate GC content genome wide in windows of 1 Mbp. We estimated gene and repeat elements densities for the final genome assembly using python script “window_quantify.py” with a window size of 1 Mbp. As the distribution of these variables (GD, GC-content, repeated elements) was highly skewed/non-normal, we performed Wilcoxon rank sum tests to check for statistically significant differences between macro and microchromosomes.

We explored broad-scale structural evolution across reptilian genomes using synteny analyses. We obtained chromosome-level genome assemblies from NCBI database (https://www.ncbi.nlm.nih.gov/assembly/?term=reptiles) for five lizards (*A. carolinensis* (GCA_000090745.2), *S. merianae* (GCA_003586115.2), *L. agilis* (GCA_009819535.1), *P. muralis* (GCA_004329235.1), and *Z. vivipara* (GCA_011800845.1)), three snakes (*C. viridis* (GCA_003400415.2), *T. elegans* (GCA_009769535.1), and *N. naja* (GCA_009733165.1)), one bird (*G. gallus* (GCA_000002315.5)), and three turtles (*T. scripta* (GCA_013100865.1), *G. evgoodei* (GCA_007399415.1), and *D. coriacea* (GCA_009764565.3)).

We used a previously established method for in silico painting [45,73] to partition the *P. platyrhinos* genome to 18.39 million 100-bp markers. As input for this approach, we used blast+2.9.0 to blast the markers against each genome (with “blastn” program and setting “-max_hsps” and “-max_target_seqs” to 1, “outfmt”=6 qseqid sseqid sstart length pident, “num_threads”=3, and the rest as default). Following Schield et al. (2019), homology signals for chromosome painting had two main conditions: 1) each marker should have an alignment length of 50 bp or greater, and 2) at least five consecutive markers must be present to infer homology (Table S5). This was determined for scaffolds from each species. For posterior analyses based on the synteny results, only the assembled chromosomes of each species (based on the

reference assembly) were considered. *Salvator merianae* was the only species in our analysis without assembled chromosomes, so we analyzed the 19 longest scaffolds (since karyotype analysis showed 2n=38) containing the majority of confirmed markers [40].

To assess the distribution of syntenic blocks of *P. platyrhinos* across scaffolds from the 12 target species, we calculated Simpson's Dominance Index (D) and its reciprocal, which, in this context, can be considered the effective number of target chromosomes (C) containing homologies from a given *P. platyrhinos* chromosome:

$$D_{ij} = \sum_{k=1}^{m} p_{ijk}^2$$

$$C_{ij} = \frac{1}{D_{ij}}$$

Where i represents a *P. platyrhinos* chromosome, j represents a target species, m is the number of scaffolds in the target species j containing homologies from the ith *P. platyrhinos* chromosome, and k represents a specific target scaffold. Values of D can range between 0 (low dominance, i.e., high spread of homologies) and 1 (full dominance, i.e., homologies remained in one target scaffold). Values of C can range between 1 (full dominance) and m (low dominance, i.e., equal spread of the ith homologies across m target scaffolds).

Availability of supporting data and materials

The chromosome-level genome assembly, annotation files, and other supporting data sets are available in the *GigaScience* database (GigaDB). Raw genomic and transcriptomic sequencing reads, and genome assembly and annotation were deposited in the NCBI under BioProject number PRJNA685451.

List of abbreviations

AED: Annotation Edit Distance

BUSCO: Benchmarking Universal Single-Copy Orthologs

C: Effective number of target Chromosomes

D: Simpson’s Dominance index

GD: Gene Density

SR: Simpson’s Reciprocal

Ethics Approval

All animal collected and euthanized according to Miami University Institutional Animal Care and Use Committee protocol 992_2021_Apr.

Competing interests

The authors declare that they have no competing interests.

Authors' contributions

N.K. and T.J. designed the project and wrote the first draft of the manuscript. N.K., A.A., K.F., D.C.C., and D.R.S. performed bioinformatics and data analyses. All authors contributed to writing and approved the final manuscript.

Acknowledgments

This work was supported by startup funds from Miami University to T. Jezkova. K. Farleigh was supported by the National Science Foundation Graduate Research Fellowship Program (Award # 2037786). We thank Aaron Ambos and Dr. Jef Jaeger for help with obtaining specimens. The analyses were performed on Miami University Redhawk cluster with incredible assistance from Dr. Jens Mueller.

References

1. Deakin, J. E., and Ezaz, T. (2019). Understanding the Evolution of Reptile Chromosomes through Applications of Combined Cytogenetics and Genomics Approaches. *Cytogenetic and Genome Research*, *157*, 7–20. https://doi.org/10.1159/000495974

2. Gemmell, N. J., Rutherford, K., Prost, S., Tollis, M., Winter, D., Macey, J. R., Adelson, D. L., Suh, A., Bertozzi, T., Grau, J. H., Organ, C., Gardner, P. P., Muffato, M., Patricio, M., Billis, K., Martin, F. J., Flicek, P., Petersen, B., Kang, L., … Stone, C. (2020). The tuatara genome reveals ancient features of amniote evolution. *Nature*, *584*, 403–409. https://doi.org/10.1038/s41586-020-2561-9

3. Burt, D. W. (2002). Origin and evolution of avian microchromosomes. *Cytogenetic and Genome Research*, *96*, 97–112. https://doi.org/10.1159/000063018

4. Waters, P. D., Simakov, O., Ezaz, T., Kaur, P., Frere, C., Grützner, F., Georges, A., Graves, J. A. M., Ruiz-herrera, A., Nicholas, C., Waters, P., Grützner, F., and Georges, A. (2021). *Microchromosomes are building blocks of bird , reptile and mammal chromosomes † Equal contribution * Corresponding author ORCID numbers of all authors Author Contributions : Competing Interest Statement : Classification : Keywords : This PDF file includes*.

5. Solinhac, R., Leroux, S., Galkina, S., Chazara, O., Feve, K., Vignoles, F., Morisson, M., Derjusheva, S., Bed'hom, B., Vignal, A., Fillon, V., and Pitel, F. (2010). Integrative mapping analysis of chicken microchromosome 16 organization. *BMC Genomics*, *11*, 616. https://doi.org/10.1186/1471-2164-11-616

6. Axelsson, E. (2005). Comparison of the chicken and turkey genomes reveals a higher rate of nucleotide divergence on microchromosomes than macrochromosomes. *Genome Research*, *15*, 120–125. https://doi.org/10.1101/gr.3021305

7. O’Connor, R. E., Kiazim, L., Skinner, B., Fonseka, G., Joseph, S., Jennings, R., Larkin, D. M., and Griffin, D. K. (2019). Patterns of microchromosome organization remain highly conserved throughout avian evolution. *Chromosoma*, *128*, 21–29. https://doi.org/10.1007/s00412-018-0685-6

8. Schield, D. R., Card, D. C., Hales, N. R., Perry, B. W., Pasquesi, G. M., Blackmon, H., Adams, R. H., Corbin, A. B., Smith, C. F., Ramesh, B., Demuth, J. P., Betrán, E., Tollis, M., Meik, J. M., Mackessy, S. P., and Castoe, T. A. (2019). The origins and evolution of chromosomes, dosage compensation, and mechanisms underlying venom regulation in snakes. *Genome Research*, *29*, 590–601. https://doi.org/10.1101/gr.240952.118

9. Schield, D. R., Pasquesi, G. I. M., Perry, B. W., Adams, R. H., Nikolakis, Z. L., Westfall, A. K., Orton, R. W., Meik, J. M., Mackessy, S. P., and Castoe, T. A. (2020). Snake Recombination Landscapes Are Concentrated in Functional Regions despite PRDM9. *Molecular Biology and Evolution*, *37*, 1272–1294. https://doi.org/10.1093/molbev/msaa003

10. Damas, J., Kim, J., Farré, M., Griffin, D. K., and Larkin, D. M. (2018). Reconstruction of avian ancestral karyotypes reveals differences in the evolutionary history of macro- and microchromosomes. *Genome Biology*, *19*, 155. https://doi.org/10.1186/s13059-018-1544-8

11. Axelsson, E., Webster, M. T., Smith, N. G. C., Burt, D. W., and Ellegren, H. (2005). Comparison of the chicken and turkey genomes reveals a higher rate of nucleotide divergence on microchromosomes than macrochromosomes. *Genome Research*, *15*, 120–125. https://doi.org/10.1101/gr.3021305

12. Perry, B. W., Schield, D. R., Adams, R. H., and Castoe, T. A. (2020). Microchromosomes Exhibit Distinct Features of Vertebrate Chromosome Structure and Function with Underappreciated Ramifications for Genome Evolution. *Molecular Biology and Evolution*, 1–7. https://doi.org/10.1093/molbev/msaa253

13. Alföldi, J., Di Palma, F., Grabherr, M., Williams, C., Kong, L., Mauceli, E., Russell, P., Lowe, C. B., Glor, R. E., Jaffe, J. D., Ray, D. A., Boissinot, S., Shedlock, A. M., Botka, C., Castoe, T. A., Colbourne, J. K., Fujita, M. K., Moreno, R. G., ten Hallers, B. F., … Lindblad-Toh, K. (2011). The genome of the green anole lizard and a comparative analysis with birds and mammals. *Nature*, *477*, 587–591. https://doi.org/10.1038/nature10390

14. Yurchenko, A. A., Recknagel, H., and Elmer, K. R. (2020). Chromosome-level assembly of the common lizard (Zootoca vivipara) genome. *Genome Biology and Evolution*. https://doi.org/10.1093/gbe/evaa161

15. Gemmell, N., Haase, B., Formenti, G., Sims, Y., Wood, J., Howe, K., Mountcastle, J., Lillie, M., Olsson, M., Rhie, A., Fedrigo, O., and Jarvis, E. D. (2019). Lacerta agilis (Sand lizard) genome, rLacAgi1, primary haplotype. *Vertebrate Genomes Project, G10K, 1230 York Avenue, New York, NY 10065, USA*.

16. Andrade, P., Pinho, C., Pérez i de Lanuza, G., Afonso, S., Brejcha, J., Rubin, C.-J., Wallerman, O., Pereira, P., Sabatino, S. J., Bellati, A., Pellitteri-Rosa, D., Bosakova, Z., Bunikis, I., Carretero, M. A., Feiner, N., Marsik, P., Paupério, F., Salvi, D., Soler, L., … Carneiro, M. (2019). Regulatory changes in pterin and carotenoid genes underlie balanced color polymorphisms in the wall lizard. *Proceedings of the National Academy of Sciences*, *116*, 5633–5642. https://doi.org/10.1073/pnas.1820320116

17. Roscito, J. G., Sameith, K., Pippel, M., Francoijs, K.-J., Winkler, S., Dahl, A., Papoutsoglou, G., Myers, G., and Hiller, M. (2018). The genome of the tegu lizard Salvator merianae : combining Illumina, PacBio, and optical mapping data to generate a highly contiguous assembly. *GigaScience*, *7*, 1–13. https://doi.org/10.1093/gigascience/giy141

18. Jezkova, T., Jaeger, J. R., Oláh-Hemmings, V., Jones, K. B., Lara-Resendiz, R. A., Mulcahy, D. G., and Riddle, B. R. (2016). Range and niche shifts in response to past climate change

in the desert horned lizard Phrynosoma platyrhinos. *Ecography*, *39*, 437–448. https://doi.org/10.1111/ecog.01464

19. Pasquesi, G. I. M., Adams, R. H., Card, D. C., Schield, D. R., Corbin, A. B., Perry, B. W., Reyes-Velasco, J., Ruggiero, R. P., Vandewege, M. W., Shortt, J. A., and Castoe, T. A. (2018). Squamate reptiles challenge paradigms of genomic repeat element evolution set by birds and mammals. *Nature Communications*, *9*, 2774. https://doi.org/10.1038/s41467-018-05279-1

20. Bronikowski, A., Fedrigo, O., Fungtammasan, C., Rhie, A., Mountcastle, J., Haase, B., Howe, K., Chow, W., Collins, J., and Jarvis, E. D. (2019). Thamnophis elegans (Western terrestrial garter snake) genome, rThaEle1, primary haplotype. *Vertebrate Genomes Project, G10K, 1230 York Avenue, New York, NY 10065, USA*.

21. Suryamohan, K., Seshagiri, S., and Guillory, J. (2019). Nana_v5. *Molecular Biology, Genentech Inc., 1 DNA Way, South San Francisco, CA 94403, USA*.

22. Hillier, L. W., Miller, W., Birney, E., Warren, W., Hardison, R. C., Ponting, C. P., Bork, P., Burt, D. W., Groenen, M. A. M., Delany, M. E., Dodgson, J. B., Chinwalla, A. T., Cliften, P. F., Clifton, S. W., Delehaunty, K. D., Fronick, C., Fulton, R. S., Graves, T. A., Kremitzki, C., … Wilson, R. K. (2004). Sequence and comparative analysis of the chicken genome provide unique perspectives on vertebrate evolution. *Nature*, *432*, 695–716. https://doi.org/10.1038/nature03154

23. Brian Simison, W., Parham, J. F., Papenfuss, T. J., Lam, A. W., and Henderson, J. B. (2020). An Annotated Chromosome-Level Reference Genome of the Red-Eared Slider Turtle (Trachemys scripta elegans). *Genome Biology and Evolution*, *12*, 456–462. https://doi.org/10.1093/gbe/evaa063

24. Murphy, B., Edwards, T., Rhie, A., Koren, S., Phillippy, A., Fedrigo, O., Haase, B.,

Mountcastle, J., Lewin, H., Damas, J., Howe, K., Formenti, G., Myers, G., Durbin, R., and Jarvis, E. D. (2019). G10K-VGP Goodes thornscrub tortoise genome, primary haplotype. *Vertebrate Genomes Project, G10K, 1230 York Avenue, New York, NY 10065, USA*.

25. Omoroske, L., Fedrigo, O., Mountcastle, J., Uliano Da Silva, M., Haase, B., Formenti, G., Chow, W., Howe, K., Gilbert, M. T. P., Flicek, P., Gemmell, N., Marques, T., Scott, A., Murphy, R., Bjorndal, K., Braun, E., Rhie, A., Phillippy, A., and Jarvis, E. D. (2019). (UPDATE)Dermochelys coriacea (Leatherback Sea Turtle) genome, rDerCor1, primary haplotype, v3. *Vertebrate Genomes Project, G10K, 1230 York Avenue, New York, NY 10065, USA*.

26. Putnam, N. H., O'Connell, B. L., Stites, J. C., Rice, B. J., Blanchette, M., Calef, R., Troll, C. J., Fields, A., Hartley, P. D., Sugnet, C. W., Haussler, D., Rokhsar, D. S., and Green, R. E. (2016). Chromosome-scale shotgun assembly using an in vitro method for long-range linkage. *Genome Research*, *26*, 342–350. https://doi.org/10.1101/gr.193474.115

27. Pianka, E. R. (1991). Phrynosoma platyrhinos Girard Desert Horned Lizard. *Catalogue of American Amphibians and Reptiles*, *1981*, 1–4.

28. Leaché, A. D., and Linkem, C. W. (2015). Phylogenomics of Horned Lizards (Genus: Phrynosoma) Using Targeted Sequence Capture Data. *Copeia*, *103*, 586–594. https://doi.org/10.1643/CH-15-248

29. Srikulnath, K., Nishida, C., Matsubara, K., Uno, Y., Thongpan, A., Suputtitada, S., Apisitwanich, S., and Matsuda, Y. (2009). Karyotypic evolution in squamate reptiles: comparative gene mapping revealed highly conserved linkage homology between the butterfly lizard (Leiolepis reevesii rubritaeniata, Agamidae, Lacertilia) and the Japanese four-striped rat snake (Elaphe quadrivirg. *Chromosome Research*, *17*, 975–986. https://doi.org/10.1007/s10577-009-9101-7

30. Rovatsos, M., Pokorná, M., Altmanová, M., and Kratochvíl, L. (2014). Cretaceous park of sex determination: sex chromosomes are conserved across iguanas. *Biology Letters*, *10*, 20131093. https://doi.org/10.1098/rsbl.2013.1093

31. Grabherr, M. G. ., Brian J. Haas, Moran Yassour Joshua Z. Levin, Dawn A. Thompson, Ido Amit, Xian Adiconis, Lin Fan, Raktima Raychowdhury, Qiandong Zeng, Zehua Chen, Evan Mauceli, Nir Hacohen, Andreas Gnirke, Nicholas Rhind, Federica di Palma, Bruce W., N., and Friedman, and A. R. (2013). Trinity: reconstructing a full-length transcriptome without a genome from RNA-Seq data. *Nature Biotechnology*, *29*, 644–652. https://doi.org/10.1038/nbt.1883.Trinity

32. Cantarel, B. L., Korf, I., Robb, S. M. C., Parra, G., Ross, E., Moore, B., Holt, C., Sanchez Alvarado, A., and Yandell, M. (2007). MAKER: An easy-to-use annotation pipeline designed for emerging model organism genomes. *Genome Research*, *18*, 188–196. https://doi.org/10.1101/gr.6743907

33. Stanke, M., and Morgenstern, B. (2005). AUGUSTUS: a web server for gene prediction in eukaryotes that allows user-defined constraints. *Nucleic Acids Research*, *33*, W465–W467. https://doi.org/10.1093/nar/gki458

34. Blum, M., Chang, H.-Y., Chuguransky, S., Grego, T., Kandasaamy, S., Mitchell, A., Nuka, G., Paysan-Lafosse, T., Qureshi, M., Raj, S., Richardson, L., Salazar, G. A., Williams, L., Bork, P., Bridge, A., Gough, J., Haft, D. H., Letunic, I., Marchler-Bauer, A., … Finn, R. D. (2021). The InterPro protein families and domains database: 20 years on. *Nucleic Acids Research*, *49*, D344–D354. https://doi.org/10.1093/nar/gkaa977

35. Smit, A., and Hubley, R. (2015). *RepeatModeler* (Open-1.0. 2008-2015). http://www.repeatmasker.org

36. Smit, AFA, Hubley, R., and Green, P. (n.d.-aj). *RepeatMasker* (Open-4.0).

http://www.repeatmasker.org

37. Mi, H., Muruganujan, A., Casagrande, J. T., and Thomas, P. D. (2013). Large-scale gene function analysis with the PANTHER classification system. *Nature Protocols*, *8*, 1551–1566. https://doi.org/10.1038/nprot.2013.092

38. Mi, H., Ebert, D., Muruganujan, A., Mills, C., Albou, L.-P., Mushayamaha, T., and Thomas, P. D. (2021). PANTHER version 16: a revised family classification, tree-based classification tool, enhancer regions and extensive API. *Nucleic Acids Research*, *49*, D394–D403. https://doi.org/10.1093/nar/gkaa1106

39. Rhie, A., McCarthy, S. A., Fedrigo, O., Damas, J., Formenti, G., Koren, S., Uliano-Silva, M., Chow, W., Fungtammasan, A., Kim, J., Lee, C., Ko, B. J., Chaisson, M., Gedman, G. L., Cantin, L. J., Thibaud-Nissen, F., Haggerty, L., Bista, I., Smith, M., … Jarvis, E. D. (2021). Towards complete and error-free genome assemblies of all vertebrate species. *Nature*, *592*, 737–746. https://doi.org/10.1038/s41586-021-03451-0

40. da Silva, M. J., de Araújo Vieira, A. P., Galvão Cipriano, F. M., dos Santos Cândido, M. R., de Oliveira, E. H. C., Gimenez Pinheiro, T., and da Silva, E. L. (2020). The Karyotype of Salvator merianae (Squamata, Teiidae): Analyses by Classical and Molecular Cytogenetic Techniques. *Cytogenetic and Genome Research*, *160*, 94–99. https://doi.org/10.1159/000506140

41. Hill, M. O. (1973). Diversity and Evenness : A Unifying Notation and Its Consequences Author (s): M . O . Hill Published by : Ecological Society of America DIVERSITY AND EVENNESS : A UNIFYING NOTATION AND ITS CONSEQUENCES '. *Ecology*, *54*, 427–432.

42. Leaché, A.D., and Sites, Jr., J. W. (2009). Chromosome Evolution and Diversification in North American Spiny Lizards (Genus *Sceloporus*). *Cytogenetic and Genome Research*, *127*, 166–181. https://doi.org/10.1159/000293285

43. Backstrom, N., Forstmeier, W., Schielzeth, H., Mellenius, H., Nam, K., Bolund, E., Webster, M. T., Ost, T., Schneider, M., Kempenaers, B., and Ellegren, H. (2010). The recombination landscape of the zebra finch Taeniopygia guttata genome. *Genome Research*, *20*, 485–495. https://doi.org/10.1101/gr.101410.109

44. Huttener, R., Thorrez, L., in't Veld, T., Granvik, M., Snoeck, L., Van Lommel, L., and Schuit, F. (2019). GC content of vertebrate exome landscapes reveal areas of accelerated protein evolution. *BMC Evolutionary Biology*, *19*, 144. https://doi.org/10.1186/s12862-019-1469-1

45. Schield, D. R., Card, D. C., Hales, N. R., Perry, B. W., Pasquesi, G. M., Blackmon, H., Adams, R. H., Corbin, A. B., Smith, C. F., Ramesh, B., Demuth, J. P., Betrán, E., Tollis, M., Meik, J. M., Mackessy, S. P., and Castoe, T. A. (2019). The origins and evolution of chromosomes, dosage compensation, and mechanisms underlying venom regulation in snakes. *Genome Research*, *29*, 590–601. https://doi.org/10.1101/gr.240952.118

46. Damas, J., Kim, J., Farré, M., Griffin, D. K., and Larkin, D. M. (2018). Reconstruction of avian ancestral karyotypes reveals differences in the evolutionary history of macro- and microchromosomes. *Genome Biology*, *19*, 155. https://doi.org/10.1186/s13059-018-1544-8

47. Axelsson, E., Webster, M. T., Smith, N. G. C., Burt, D. W., and Ellegren, H. (2005). Comparison of the chicken and turkey genomes reveals a higher rate of nucleotide divergence on microchromosomes than macrochromosomes. *Genome Research*, *15*, 120–125. https://doi.org/10.1101/gr.3021305

48. Smith, J., Bruley, C. K., Paton, I. R., Dunn, I., Jones, C. T., Windsor, D., Morrice, D. R., Law, A. S., Masabanda, J., Sazanov, A., Waddington, D., Fries, R., and Burt, D. W. (2000). Differences in gene density on chicken macrochromosomes and microchromosomes. *Animal Genetics*, *31*, 96–103. https://doi.org/10.1046/j.1365-2052.2000.00565.x

49. Kuraku, S., Ishijima, J., Nishida-Umehara, C., Agata, K., Kuratani, S., and Matsuda, Y. (2006).

cDNA-based gene mapping and GC3 profiling in the soft-shelled turtle suggest a chromosomal size-dependent GC bias shared by sauropsids. *Chromosome Research*, *14*, 187–202. https://doi.org/10.1007/s10577-006-1035-8

50. Lieberman-Aiden, E., van Berkum, N. L., Williams, L., Imakaev, M., Ragoczy, T., Telling, A., Amit, I., Lajoie, B. R., Sabo, P. J., Dorschner, M. O., Sandstrom, R., Bernstein, B., Bender, M. A., Groudine, M., Gnirke, A., Stamatoyannopoulos, J., Mirny, L. A., Lander, E. S., and Dekker, J. (2009). Comprehensive Mapping of Long-Range Interactions Reveals Folding Principles of the Human Genome. *Science*, *326*, 289–293. https://doi.org/10.1126/science.1181369

51. Zaharia, M., Bolosky, W. J., Curtis, K., Fox, A., Patterson, D., Shenker, S., Stoica, I., Karp, R. M., and Sittler, T. (2011). *Faster and More Accurate Sequence Alignment with SNAP*. 1–10. http://arxiv.org/abs/1111.5572

52. Leaché, Adam D., Banbury, B. L., Linkem, C. W., and de Oca, A. N.-M. (2016). Phylogenomics of a rapid radiation: is chromosomal evolution linked to increased diversification in north american spiny lizards (Genus Sceloporus)? *BMC Evolutionary Biology*, *16*, 63. https://doi.org/10.1186/s12862-016-0628-x

53. Camacho, C., Coulouris, G., Avagyan, V., Ma, N., Papadopoulos, J., Bealer, K., and Madden, T. L. (2009). BLAST+: architecture and applications. *BMC Bioinformatics*, *10*, 421. https://doi.org/10.1186/1471-2105-10-421

54. Rice, E. S., Kohno, S., John, J. St., Pham, S., Howard, J., Lareau, L. F., O'Connell, B. L., Hickey, G., Armstrong, J., Deran, A., Fiddes, I., Platt, R. N., Gresham, C., McCarthy, F., Kern, C., Haan, D., Phan, T., Schmidt, C., Sanford, J. R., … Green, R. E. (2017). Improved genome assembly of American alligator genome reveals conserved architecture of estrogen signaling. *Genome Research*, *27*, 686–696. https://doi.org/10.1101/gr.213595.116

55. Bao, W., Kojima, K. K., and Kohany, O. (2015). Repbase Update, a database of repetitive elements in eukaryotic genomes. *Mobile DNA*, *6*, 11. https://doi.org/10.1186/s13100-015-0041-9

56. Simão, F. A., Waterhouse, R. M., Ioannidis, P., Kriventseva, E. V., and Zdobnov, E. M. (2015). BUSCO: assessing genome assembly and annotation completeness with single-copy orthologs. *Bioinformatics*, *31*, 3210–3212. https://doi.org/10.1093/bioinformatics/btv351

57. Georges, A., Li, Q., Lian, J., O'Meally, D., Deakin, J., Wang, Z., Zhang, P., Fujita, M., Patel, H. R., Holleley, C. E., Zhou, Y., Zhang, X., Matsubara, K., Waters, P., Graves, J. A. M., Sarre, S. D., and Zhang, G. (2015). High-coverage sequencing and annotated assembly of the genome of the Australian dragon lizard Pogona vitticeps. *GigaScience*, *4*, 45. https://doi.org/10.1186/s13742-015-0085-2

58. Liu, Y., Zhou, Q., Wang, Y., Luo, L., Yang, J., Yang, L., Liu, M., Li, Y., Qian, T., Zheng, Y., Li, M., Li, J., Gu, Y., Han, Z., Xu, M., Wang, Y., Zhu, C., Yu, B., Yang, Y., … Gu, X. (2015). Gekko japonicus genome reveals evolution of adhesive toe pads and tail regeneration. *Nature Communications*, *6*, 10033. https://doi.org/10.1038/ncomms10033

59. Castoe, T. A., de Koning, A. J., Hall, K. T., Yokoyama, K. D., Gu, W., Smith, E. N., Feschotte, C., Uetz, P., Ray, D. A., Dobry, J., Bogden, R., Mackessy, S. P., Bronikowski, A. M., Warren, W. C., Secor, S. M., and Pollock, D. D. (2011). Sequencing the genome of the Burmese python (Python molurus bivittatus) as a model for studying extreme adaptations in snakes. *Genome Biology*, *12*, 406. https://doi.org/10.1186/gb-2011-12-7-406

60. Edwards J, R. (2018). Direct Submission. *UNIVERSITY OF NEW SOUTH WALES, Edwards Lab, E26/2110 BioScience South, UNSW SYDNEY, NSW 2052, Australia*.

61. Aird, S. D., Arora, J., Barua, A., Qiu, L., Terada, K., and Mikheyev, A. S. (2017). Population Genomic Analysis of a Pitviper Reveals Microevolutionary Forces Underlying Venom

Chemistry. *Genome Biology and Evolution*, *9*, 2640–2649. https://doi.org/10.1093/gbe/evx199

62. Warren, W. C., and Wilson, R. K. (2015). Direct Submission. *The Genome Institute, Washington University School of Medicine, 4444 Forest Park, St. Louis, MO 63108, USA*.

63. St John, J. A., Braun, E. L., Isberg, S. R., Miles, L. G., Chong, A. Y., Gongora, J., Dalzell, P., Moran, C., Bed'Hom, B., Abzhanov, A., Burgess, S. C., Cooksey, A. M., Castoe, T. A., Crawford, N. G., Densmore, L. D., Drew, J. C., Edwards, S. V., Faircloth, B. C., Fujita, M. K., … Ray, D. A. (2012). Sequencing three crocodilian genomes to illuminate the evolution of archosaurs and amniotes. *Genome Biology*, *13*, 415. https://doi.org/10.1186/gb-2012-13-1-415

64. Wan, Q., Pan, S., Hu, L., Zhu, Y., Xu, P., Xia, J., Chen, H., He, G., He, J., Ni, X., Hou, H., Liao, S., Yang, H., Chen, Y., Gao, S., Ge, Y., Cao, C., Li, P., Fang, L., … Fang, S. (2013). The genome sequence of Chinese alligator (Alligator sinensis). *BGI-Shenzhen, Beishan Industrial Zone, Yantian District, Shenzhen, Guangdong 518083, China*.

65. Pham, S., Fiddes, I., Deran, A., Armstrong, J., Rice, E. S., and Paten, B. (2016). Comparative assembly of saltwater crocodile genome. *Biomolecular Engineering, University of California, Santa Cruz, 1156 High St., Santa Cruz, CA 95064, USA*.

66. Badenhorst, D., Hillier, L. W., Literman, R., Montiel, E. E., Radhakrishnan, S., Shen, Y., Minx, P., Janes, D. E., Warren, W. C., Edwards, S. V., and Valenzuela, N. (2015). Physical Mapping and Refinement of the Painted Turtle Genome (Chrysemys picta) Inform Amniote Genome Evolution and Challenge Turtle-Bird Chromosomal Conservation. *Genome Biology and Evolution*, *7*, 2038–2050. https://doi.org/10.1093/gbe/evv119

67. Deem, S. L., and Warren, W. C. (2018). Direct Submission. *McDonnell Genome Institute, Washington University School of Medicine, 4444 Forest Park, St. Louis, MO 63108, USA*.

68. Wang, Z., Pascual-anaya, J., Zadissa, A., Li, W., and Niimura, Y. (2014). *Europe PMC Funders Group The draft genomes of soft – shell turtle and green sea turtle yield insights into the development and evolution of the turtle – specific body plan*. *45*, 701–706. https://doi.org/10.1038/ng.2615.The

69. Venter, J. C., Adams, M. D., Myers, E. W., Li, P. W., Mural, R. J., Sutton, G. G., Smith, H. O., Yandell, M., Evans, C. A., Holt, R. A., Gocayne, J. D., Amanatides, P., Ballew, R. M., Huson, D. H., Wortman, J. R., Zhang, Q., Kodira, C. D., Zheng, X. H., Chen, L., … Zhu, X. (2001). The Sequence of the Human Genome. *Science*, *291*, 1304–1351. https://doi.org/10.1126/science.1058040

70. Brent, M. R., Birren, B. W., Antonarakis, S. E., Alexandersson, M., Zody, M., Birney, E., Baertsch, R., Cuff, J., Parra, G., Slater, G., Waterston, R. H., Lindblad-Toh, K., Rogers, J., Abril, J. F., Agarwal, P., Agarwala, R., Ainscough, R., An, P., Attwood, J., … Lander, E. S. (2002). Initial sequencing and comparative analysis of the mouse genome. *Nature*, *420*, 520–562. http://www.nature.com/nature/journal/v420/n6915/full/nature01262.html

71. Bateman, A. (2019). UniProt: a worldwide hub of protein knowledge. *Nucleic Acids Research*, *47*, D506–D515. https://doi.org/10.1093/nar/gky1049

72. Jones, P., Binns, D., Chang, H.-Y., Fraser, M., Li, W., McAnulla, C., McWilliam, H., Maslen, J., Mitchell, A., Nuka, G., Pesseat, S., Quinn, A. F., Sangrador-Vegas, A., Scheremetjew, M., Yong, S.-Y., Lopez, R., and Hunter, S. (2014). InterProScan 5: genome-scale protein function classification. *Bioinformatics*, *30*, 1236–1240. https://doi.org/10.1093/bioinformatics/btu031

73. McKenna, D. D., Scully, E. D., Pauchet, Y., Hoover, K., Kirsch, R., Geib, S. M., Mitchell, R. F., Waterhouse, R. M., Ahn, S.-J., Arsala, D., Benoit, J. B., Blackmon, H., Bledsoe, T., Bowsher, J. H., Busch, A., Calla, B., Chao, H., Childers, A. K., Childers, C., … Richards, S.

(2016). Genome of the Asian longhorned beetle (Anoplophora glabripennis), a globally significant invasive species, reveals key functional and evolutionary innovations at the beetle–plant interface. *Genome Biology*, *17*, 227. https://doi.org/10.1186/s13059-016-1088-8

74. Hedges, S. B., Dudley, J., and Kumar, S. (2006). TimeTree: a public knowledge-base of divergence times among organisms. *Bioinformatics*, *22*, 2971–2972. https://doi.org/10.1093/bioinformatics/btl505

FIGURES

Figure 1. For each major clade, we list diploid chromosome numbers, macrochromosome numbers, and microchromosome numbers based on previous research [1]. The phylogeny was adapted from [2].

Figure 2. The genome content of *P. platyrhinos.* The outer circle shows gene density on each chromosome, the middle circle shows repeat element density, and the inner one shows GC content. Each estimate is calculated per 1 million base pair window in each chromosome. “Ma” indicates macrochromosomes and “mi” stands for microchromosomes. Two scaffolds for macrochromosome 3 are attached together (the black line) and two microchromosomes (mi6 and mi10) resulted from a single scaffold were showed separately and in size order with the rest of the microchromosomes.

Figure 3. Synteny between *P. platyrhinos* and 12 reptilian taxa: three snakes (*N. naja*, *T. elegance*, and *C. viridis*), five lizards *(A. carolinensis, L. agilis, Z. vivipara, P. muralis*, and *S. merianae*), three turtles (*T. scripta, G. evgoodei*, and *D. coriacea*), and a bird (*G. gallus*). The cladogram shows the phylogenetic relationships among the sampled taxa [74] (two scaffolds for macrochromosome 3 (3a and 3b) are concatenated in this figure).

Figure 4. Effective number of chromosomes (C) assessed using the dominance analysis. Values close to 1 represent full dominance (homologies from a given *P. platyrhinos* chromosome are contained within a single chromosome/scaffold of another species). Values higher than 1 mean a spread of homologies across multiple chromosomes/scaffolds.

Figure 5. Summary of the effective number of chromosomes of *P. platyrhinos* in comparison with the 12 target species based on SR a) Mean and SD of SR for each chromosome among 12 species. Values close to 1 represent full dominance (homologies from a given *P. platyrhinos* chromosome are contained within a single chromosome/scaffold). Values higher than 1 mean a spread of homologies across multiple chromosomes/scaffolds. b) Cumulative SR for chromosomes of 12 reptilian species. The total amount of SR at greater phylogenetic distances, is higher (cumulative SR ~ 30 in turtles) and showing greater rearrangements and partitions of syntenic blocks in macrochromosomes than in microchromosomes

Figure 6. Synteny between *P. platyrhinos* potential microchromosomes (before assigning scaffolds to specific chromosomes) and the 12 reptilian genomes. The cladogram shows the phylogenetic relationships among the assessed taxa [74].

Figure S1: Repeat elements, GC content, and gene density calculated in 1Mb windows for each chromosome of *P. platyrhinos* (two scaffolds for macrochromosome 3 are concatenated) *.*

Figure S2: Proportion of identified gene IDs from protein-coding annotation to unidentified gene IDs by PANTHER a) across the chromosomes (Ma stands for macrochromosome, and mi stands for microchromosome). b) between two groups of chromosomes (Macros = macrochromosomes, and Micros = microchromosomes).

Figure S2. Investigating potential misassembled point on a final scaffold. a) Chicago scaffolds assembled to a final scaffold “Sc4326_4427” were used to investigate a possible misassembled point. b) repeat elements, GC content, and gene density calculated in 1Mb windows were used as evidence to find break point on this final scaffold. Outlined cells are where the breakpoint was placed. Then microchromosomes were numbered based on size so these two scaffolds were numbered as microchromosome 10 (left portion) and microchromosome 6 (right portion).

Figure S4: Distribution of *P. platyrhinos* total annotated protein coding genes with identified IDs in PANTHER database. Among 164 PANTHER pathways assigned to *P. platyrhinos* protein coding genes, each pathway accounts for different number of genes (2< genes per pathway <759) that may belong to a specific chromosome (24 pathways only on macrochromosomes, and 3 only on microchromosomes) or group of chromosomes (13 pathways only in macrochromosomes group).

TABLES

Table 1. Basic information about the *P. platyrhinos* genome assembly.

Assembly	Chicago Assembly	Chicago + Hi-C Assembly
Longest Scaffold (bp)	361,415,485	396,190.715
Number of Scaffolds	5,458	5,294
Number of Scaffolds > 1 kb	5,458	5,294
Contig N50 (kb)	12.04	12.04
Scaffold N50 (kb)	63,431	273,213
Number of Gaps	258,150	258,317
Percent of Genome in Gaps	1.54%	1.54%

Table 2: BUSCO summary results.

BUSCO benchmark	Number	Percentage
**Present BUSCOs**	4,324	81.5%
Complete BUSCOs	3640	68.6%
Complete single-copy BUSCOs	3609	68.0%
Complete duplicated BUSCOs	31	0.6%
Fragmented BUSCOs	684	12.9%
**Missing BUSCOs**	986	18.5%
Total BUSCO groups searched	5310	100

Table 3**.** Sequencing libraries used for the genome assembly of *P. platyrhinos.*

Library	Read Type	Number of Reads	Assembly Version	NCBI accession number
Shotgun library 1 (150 bp)	paired end	311,540,000	Primary	SRR16071941
Shotgun library 2 (150 bp)	paired end	239,630,000	Primary	SRR16071940
Shotgun library 3 (150 bp)	paired end	308,750,000	Primary	SRR16071939
Chicago library 1 (151 bp)	paired end	402,000,000	Intermediate	SRR13811242
Chicago library 2 (151 bp)	paired end	398,000,000	Intermediate	SRR13811241
Chicago library 3 (151 bp)	paired end	256,000,000	Intermediate	SRR13811240
Hi-C library 1 (151 bp)	paired end	332,000,000	Final	SRR13811239
Hi-C library 2 (151 bp)	paired end	374,000,000	Final	SRR13811238
Hi-C library 3 (151 bp)	paired end	324,000,000	Final	SRR13811237

Table 4. Number of reads obtained from 8 tissues of *P. platyrhinos*, used for transcriptome assembly.

Sample ID	Tissue	Raw Reads	Quality Trimmed Reads	NCBI accession number
TRO180600001	liver	49,736,350	47,699,266	SRR13326553
TRO180600002	lungs	40,643,066	39,124,052	SRR13326552
TRO180600003	brain	85,097,044	81,754,486	SRR13326551
TRO180600004	muscle	37,712,026	34,653,428	SRR13326550
TRO180600005	testes	62,536,762	58,283,654	SRR13326549
TRO180600006	heart	34,757,154	32,027,338	SRR13326548
TRO180600007	eyes	46,140,488	42,334,272	SRR13326547
TRO180600008	kidneys	41,776,926	38,635,176	SRR13326546

Table S1. The corresponding scaffolds (first column) for each chromosome of *P. platyrhinos* (second column) and scaffold length (third column) in base pairs. *This scaffold was broken down into two microchromosomes (6 and 10).

Scaffold name	Chromosome(s) name	length (in base pairs)
Sc3291_377	Chromosome 1	396,190,715
Sc439 _455	Chromosome 2	336,734,411
Sc1234_1274	Chromosome 3-a	178,616,284
Sc1882_1940	Chromosome 3-b	123,146,639
Sc5292_5410	Chromosome 4	273,212,746
Sc5293_5450	Chromosome 5	219,432,639
Sc521_540	Chromosome 6	129,273,435
Sc3285_3371	Microchromosome 1	31,685,405
Sc3778_3872	Microchromosome 2	28,086,253
Sc415_430	Microchromosome 3	27,277,973
Sc35_37	Microchromosome 4	27,087,043
Sc3441_3531	Microchromosome 5	26,097,904
Sc4326_4427*		
Sc4326a4427	Microchromosome 10	11,894,615
Sc4326b4427	microchromosome 6	23,702,528
Sc26_27	Microchromosome 7	20,466,995
Sc5294_5452	Microchromosome 8	16,009,790
Sc1213_1253	Microchromosome 9/X	15,721,303
Sc953_986	Microchromosome 11	8,897,685

Table S2. Best blast hits of cDNA [29] and * indicates sex linked markers [30] from *A. carolinensis* and *L. reevesii* against the genome of *P. platyrhinos*.

Marker	Accession	Chromosomal location			E-value
		A. carolinensis	*L. reevesii*	*P. platyrhinos*	
DYNC1H1	AB490348		1q	Chr1	2.95E-179
ESR1	AB490345		1p	Chr1	1.02E-113
WT1	XM_016992885	1		Chr1	2.19E-158
WT1	AB490347		1q	Chr1	7.53E-80
XAB1	AB490344		1p	Chr1	2.31E-35
CHD1	XM_008103079	2		Chr2	0
CHD1	AB480289		2p	Chr2	1.25E-144
DMRT1	XM_003216553	2		Chr2	0
DMRT1	AB480288		2p	Chr2	2.15E-64
GHR	XM_008102837	2		Chr2	0
GHR	AB480290		2p	Chr2	1.01E-104
RPS6	XM_003216606	2		Chr2	5.32E-123
RPS6	AB480287		2p	Chr2	2.39E-88
RUFY1	XM_008104854	2		Chr2	0
RUFY1	AB490352		2q	Chr2	3.45E-22
EIF2S3	XM_003218845	3		Chr3-a	0
EIF2S3	AB490361		3q	Chr3-a	5.58E-104
OCA2	XM_008107106	3		Chr3-a	0
OCA2	AB490360		3q	Chr3-a	1.78E-89
SH3PXD2A	XM_016992171	3		Chr3-b	0
SH3PXD2A	AB490356		3p	Chr3-b	5.98E-166
TLOC1	AB490355	3p		Chr3-b	1.71E-79
HDAC3	XM_003219886	4		Chr4	0
HDAC3	AB490365		4p	Chr4	4.16E-97
RBM12	XM_008109953	4		Chr4	0
RBM12	AB490367		4q	Chr4	3.92E-137

SS18	XM_003219645	4		Chr4	0
SS18	AB490397		4p	Chr4	1.75E-70
ZNF326	XM_008109275	4		Chr4	0
ZNF326	AB490366		4q	Chr4	1.00E-128
ACSL1	XM_008111814	5		Chr5	0
ACSL1	AB490370		5p	Chr5	1.00E-95
DCLK2	XM_008111991	5		Chr5	0
DCLK2	AB490369		5p	Chr5	2.06E-73
EXOC1	XM_008111693	5		Chr5	0
EXOC1	AB490371		5p	Chr5	3.08E-176
RANGAP1	XM_008110743	5		Chr5	0
RANGAP1	AB490374		5q	Chr5	6.70E-80
SOX5	XM_008110345	5		Chr5	0
SOX5	AB490376		5q	Chr5	1.78E-104
UCHL1	XM_003221541	5		Chr5	2.55E-63
UCHL1	AB490372		5p	Chr5	3.46E-59
CTNNB1	AB490379		6q	Chr6	0
GAD2	XM_003222133	6		Chr6	0
GAD2	AB490380		6q	Chr6	1.98E-76
MYST2	AB490378		6p	Chr6	0
WAC	XM_008112381	6		Chr6	0
WAC	AB490381		6q	Chr6	3.60E-159
AR	AB490385		micro	microchr3	2.72E-152
TMEM132D*	XM_008113640.2	micro “b”/X		microchr9/X	0
FZD10*	XM_003222753.3	micro “b”/X		microchr9/X	0
ATP2A2*	XM_008113715	micro “b”/X		microchr9/X	0
ATP2A2	AB490391		micro	microchr9/X	4.05E-167
ATRX	AB490386		micro	microchr3	7.88E-127
BRD7	AB490390		micro	microchr2	3.95E-68
HSPA8	XM_003222794	micro “a”		Chr1	0
HSPA8	AB490395		micro	microchr4	3.70E-162

Table S3. Number, length, and percentage of annotated repeat elements identified.

Families of repeat elements	Numbers of elements	Length masked (bp)	% of sequence	% element masked
**Retroelements**	2,082,017	451,287,018	23.83	20.37
**SINEs**	648,720	89,280,596	4.72	6.35
Penelope	254,722	35,799,757	1.89	2.50
**LINEs**	1,311,944	319,965,632	16.90	12.84
L2/CR1/Rex	702,907	160,952,766	8.50	6.88
R1/LOA/Jockey	36	3,068	0.00	0.00
R2/R4/NeSL	5,129	640,551	0.03	0.05
RTE/Bov-B	257,696	83,172,778	4.39	2.52
L1/CIN4	87,958	38,708,200	2.04	0.86
**LTR elements**	121,353	42,040,790	2.22	1.19
BEL/Pao	4,074	768,559	0.04	0.04
Ty1/Copia	18,376	7,918,963	0.42	0.18
Gypsy/DIRS1	39,227	14,661,509	0.77	0.38
Retroviral	34,521	5,663,234	0.30	0.34
**DNA transposons**	1,527,111	204,435,133	10.80	14.94
hobo-Activator	610,832	73,847,731	3.90	5.98
Tc1-IS630-Pogo	314,462	42,728,561	2.26	3.08
PiggyBac	1,795	445,424	0.02	0.02
Tourist/Harbinger	500,329	78,020,620	4.12	4.90
**Unclassified**	828,472	146,176,330	7.72	8.11
**Total interspersed repeats**	9,351,681	801,898,481	42.35	91.51
Small RNA	33,490	3,376,969	0.18	0.33
Satellites	51,860	7,242,936	0.38	0.51
Simple repeats	705,413	27,116,672	1.43	6.90
Low complexity	77,452	3,957,871	0.21	0.76

**Total masked**	10,219,896	841,750,763	44.45	100.00

Table S4: Comparison of molecular pathways analysis on macrochromosomes and microchromosomes. Second column shows the specific pathways identified on each chromosome. Third column shows the pathways that belong to specific group of chromosomes.

Chromosome location	Specific pathways for each chromosome	Specific pathways for macros versus micros
Chromosome 1	Allantoin degradation (P02725), Methionine biosynthesis (P02753)	5-Hydroxytryptamine biosynthesis (P04371), Acetate utilization (P02722), Activin beta signaling pathway (P06210), Anandamide degradation (P05728), Androgen/estrogene/progesterone biosynthesis (P02727), Ascorbate degradation (P02729), ATP synthesis (P02721), Biotin biosynthesis (P02731), BMP/activin signaling pathway-drosophila (P06211), DPP signaling pathway (P06213), DPP-SCW signaling pathway (P06212), Glutamine glutamate conversion (P02745), Isoleucine biosynthesis (P02748), Leucine biosynthesis (P02749), Methylmalonyl pathway (P02755), Proline biosynthesis (P02768), Purine metabolism (P02769), Pyridoxal phosphate salvage pathway (P02770), Pyridoxal-5-phosphate biosynthesis (P02759), SCW signaling pathway (P06216), Succinate to proprionate conversion (P02777), Toll pathway-drosophila (P06217), Valine biosynthesis (P02785), and Vitamin B6 metabolism (P02787)
Chromosome 2	ALP23B signaling pathway (P06209), GBB signaling pathway (P06214), MYO signaling pathway (P06215)	
Chromosome 3	Cysteine biosynthesis (P02737), Lysine biosynthesis (P02751)	
Chromosome 4	Thiamin metabolism (P02780)	
Chromosome 5	Cobalamin biosynthesis (P02735), Sulfate assimilation (P02778)	
Chromosome 6	Carnitine metabolism (P02733), Coenzyme A linked carnitine metabolism (P02732), and Threonine biosynthesis (P02781)	
Microchromosome 1	None.	None.
Microchromosome 2	Tyrosine biosynthesis (P02784)	
Microchromosome 3	None.	

Microchromosome 4	Bupropion degradation (P05729)
Microchromosome 5	Triacylglycerol metabolism (P02782)
Microchromosome 6	None.
Microchromosome 7	None.
Microchromosome 8	None.
Microchromosome 9/X	None.
Microchromosome10	None
Microchromosome 11	None.

Table S5. Genome assemblies and number of markers used for *in silico* painting. All assemblies are available through NCBI under the appropriate accession.

Organism	Potential single markers	Total confirmed (5 consecutive) markers	Scaffolds with confirmed homologies	Confirmed markers in Scaffolds (%)	Assembly accession
A. carolinensis	2,616,045	87,155	13	57,006 (65.41)	GCA_000090745.2
S. merianae	390,847	31,955	19	31,805 (99.53)	GCA_003586115.2
L. agilis	755,639	44,200	20	44,199 (99.99)	GCA_009819535.1
P. muralis	719,822	46,093	19	45,731 (99.21)	GCA_004329235.1
Z. vivipara	751,121	43,371	19	42,224 (97.35)	GCA_011800845.1
C. viridis	299,173	18,161	18	17,891 (98.51)	GCA_003400415.2
T. elegans	282,458	17,817	18	17,725 (99.48)	GCA_009769535.1
N. naja	291, 209	19,898	19	19,805 (99.52)	GCA_009733165.1
T. scripta	177,241	15,287	25	15,252 (99.77)	GCA_013100865.1
G. evgoodei	152,748	14,864	24	14,614 (98.32)	GCA_007399415.1
D. coriacea	137,161	14,075	29	14,075 (100.00)	GCA_009764565.3
G. gallus	88,397	10,934	33	10,934 (100.00)	GCA_000002315.5

Figure1

Click here to access/download;Figure;fig1.docx

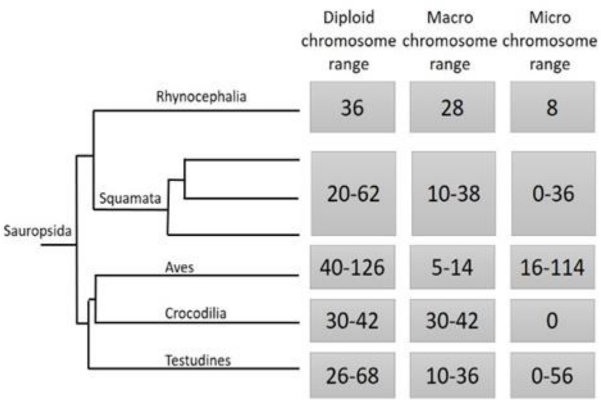

Figure2 Click here to access/download;Figure;Figure2.jpg

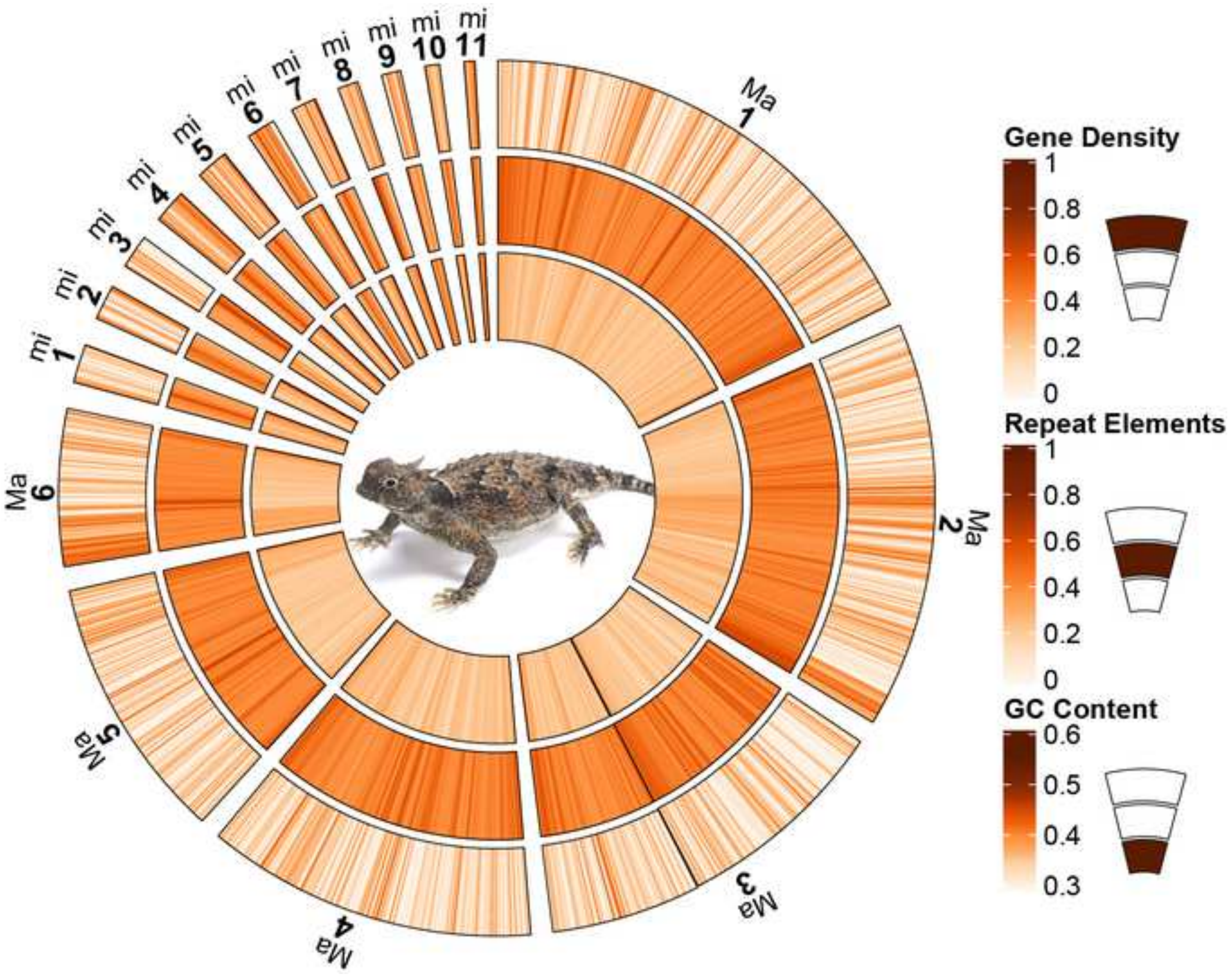

Figure3

Click here to access/download;Figure;Figure3.jpg

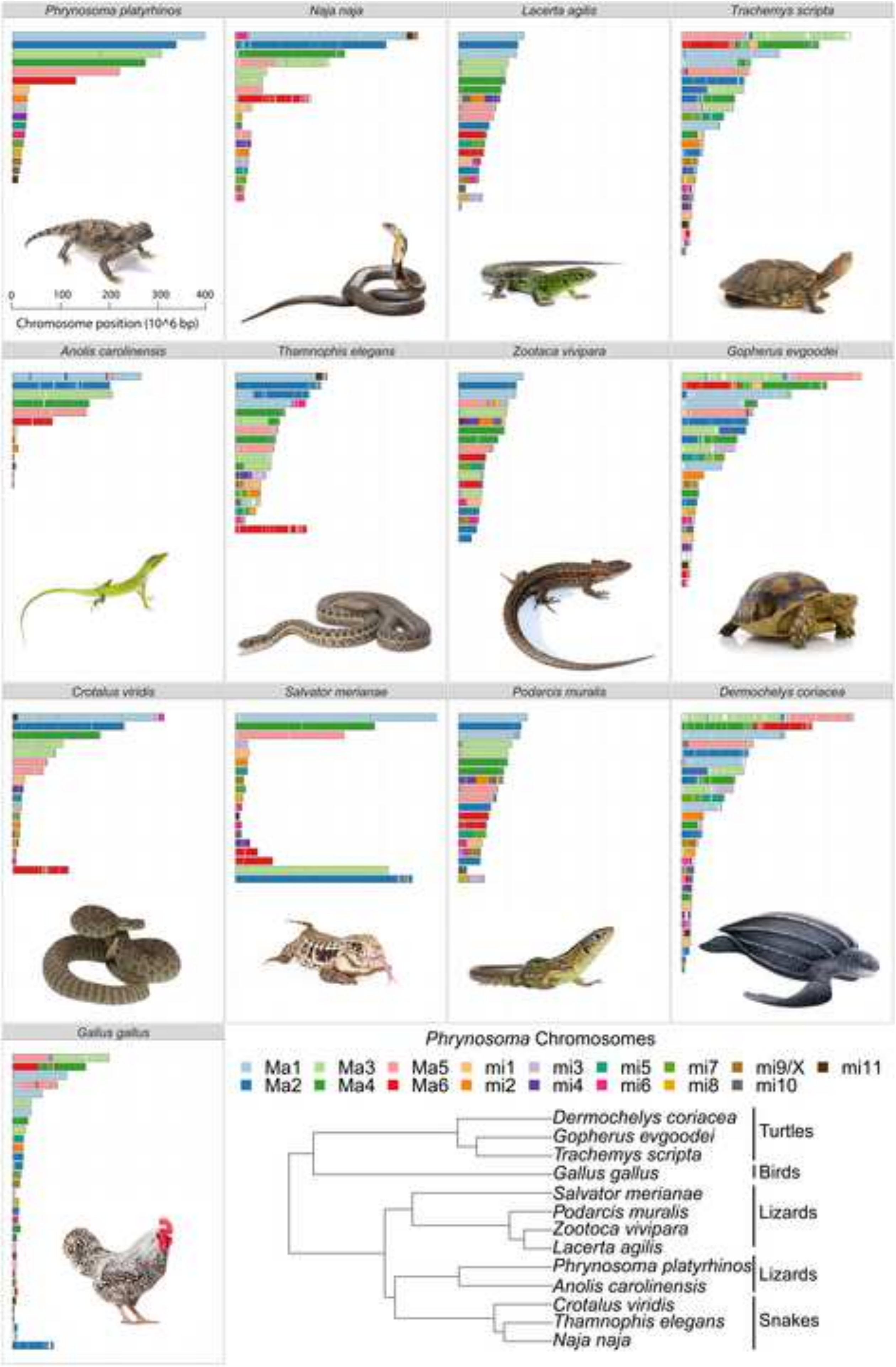

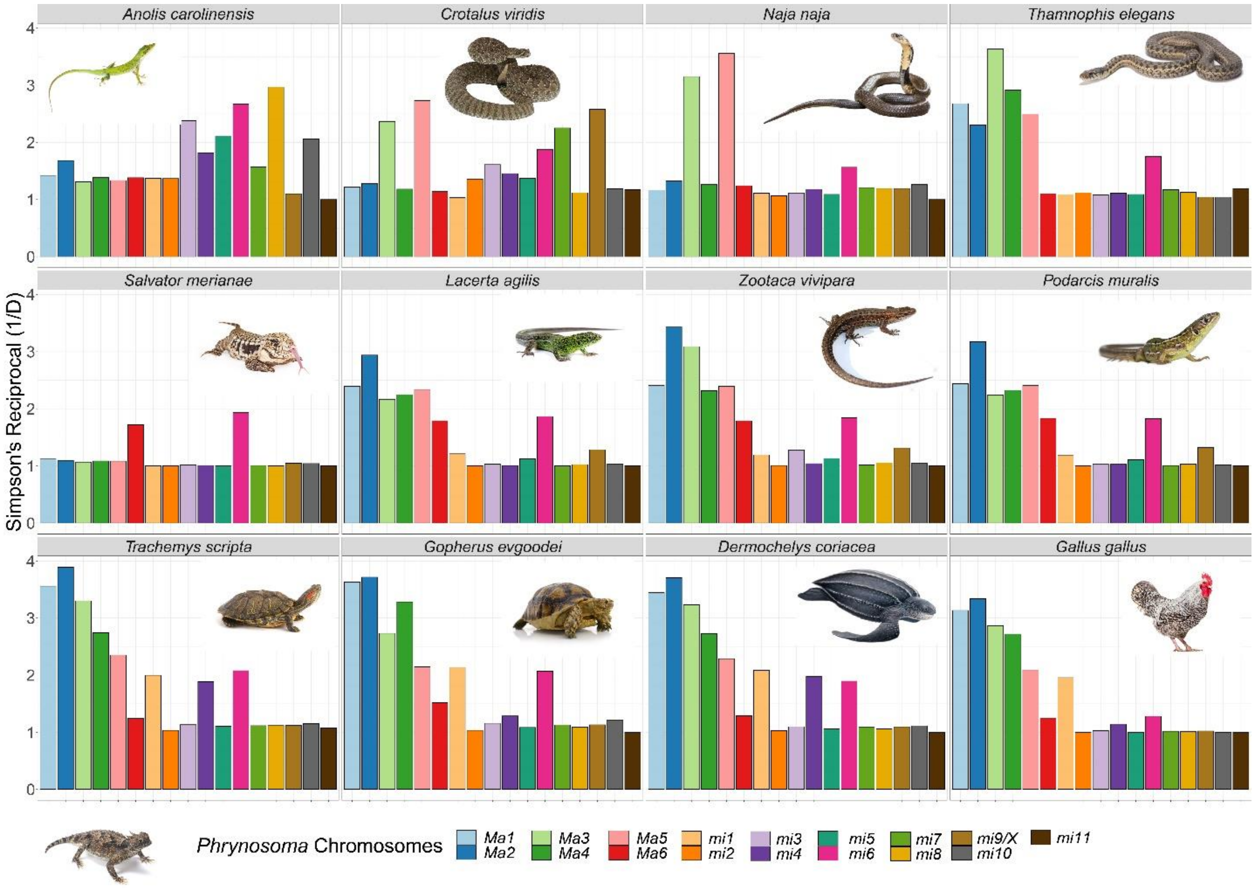
Anolis carolinensis
Crotalus viridis
Naja naja
Thamnophis elegans
Salvator merianae
Lacerta agilis
Zootaca vivipara
Podarcis muralis
Trachemys scripta
Gopherus evgoodei
Dermochelys coriacea
Gallus gallus
Simpson's Reciprocal (1/D)
0
1
2
3
4
Phrynosoma Chromosomes
Ma1
Ma2
Ma3
Ma4
Ma5
Ma6
mi1
mi2
mi3
mi4
mi5
mi6
mi7
mi8
mi9/X
mi10
mi11

Click here to access/download;Figure;Figure5a.jpg

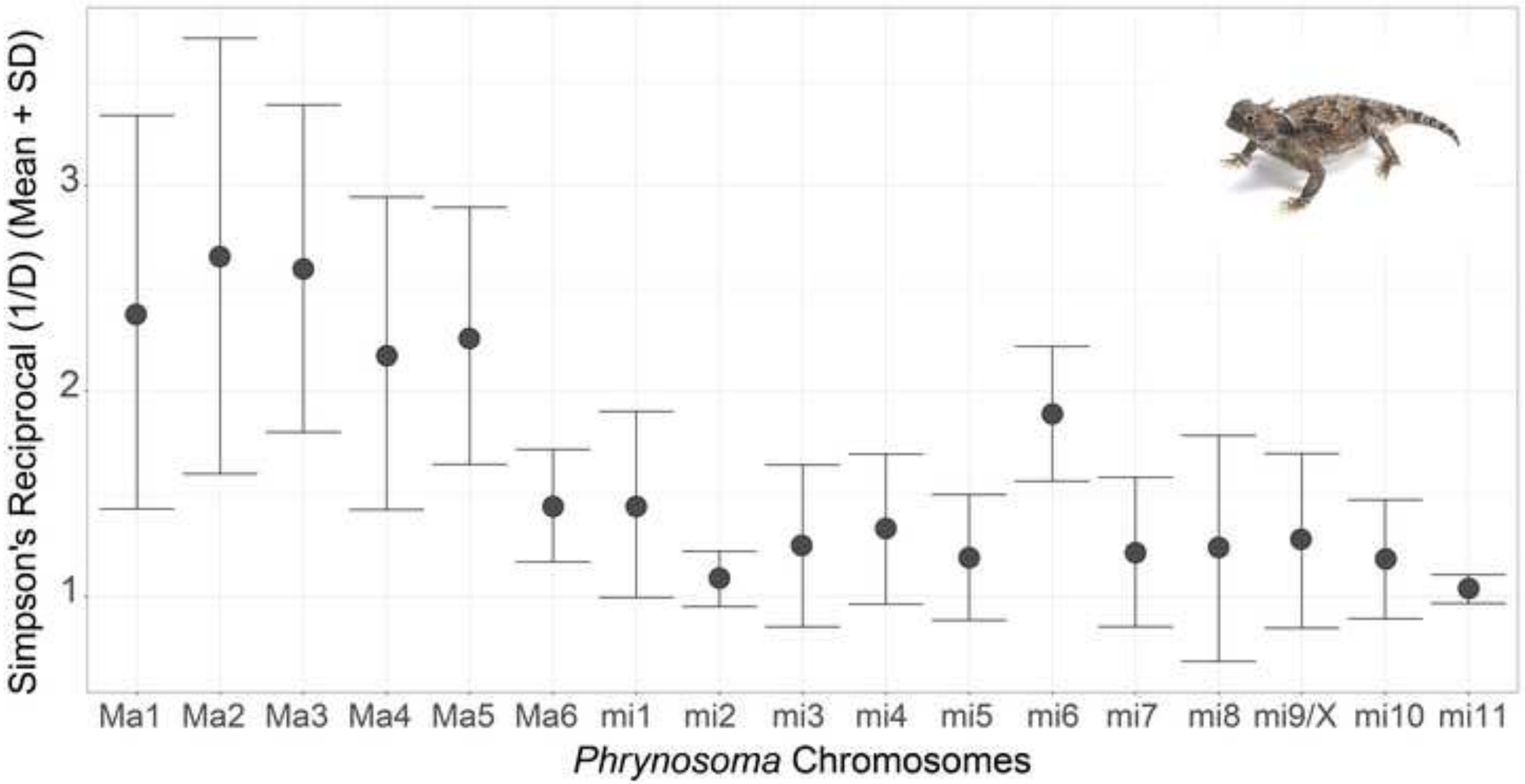

Figure5b

Click here to access/download;Figure;Figure5b.jpg

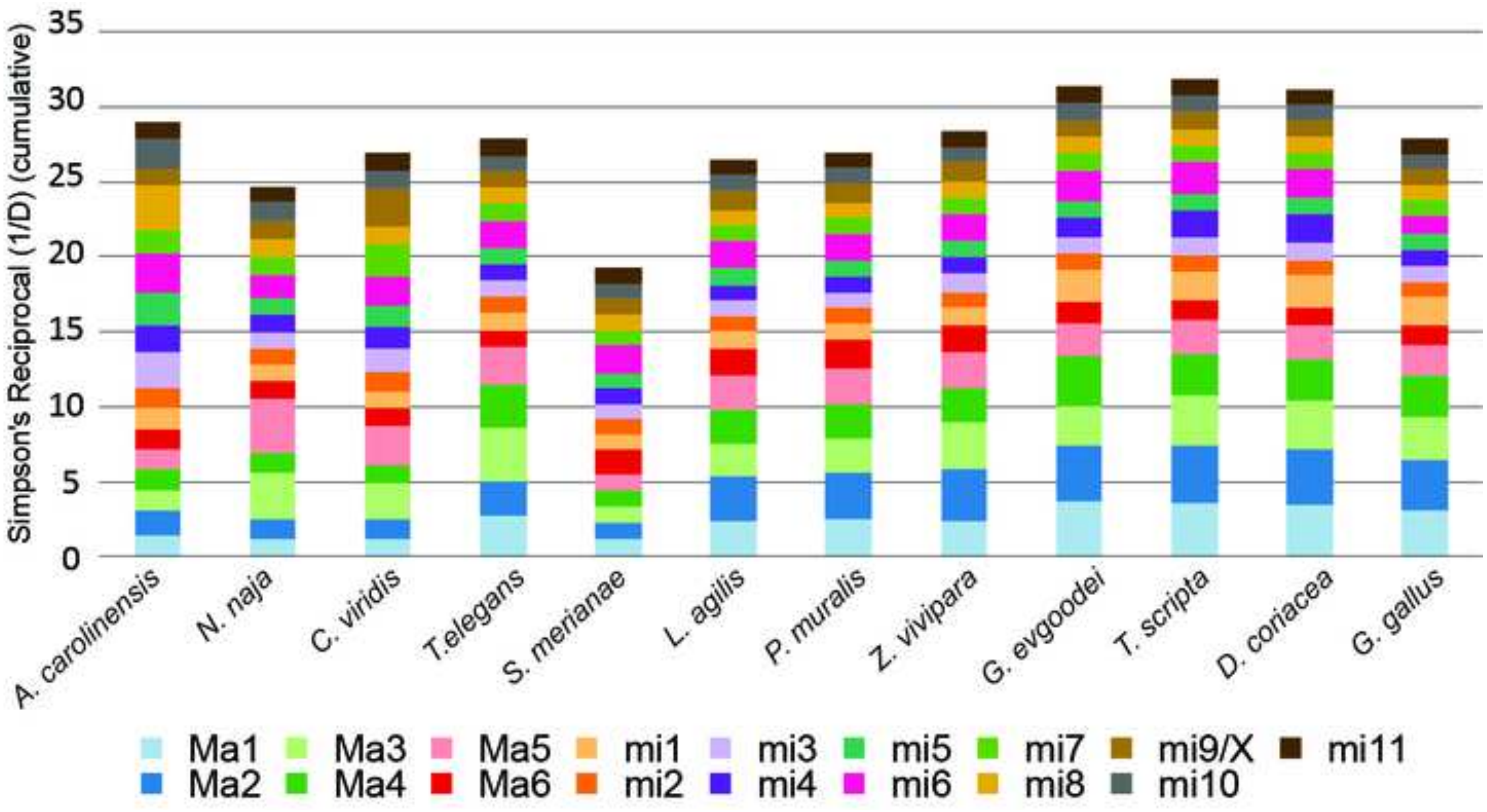

Figure6 Click here to access/download;Figure;Figure6.jpg

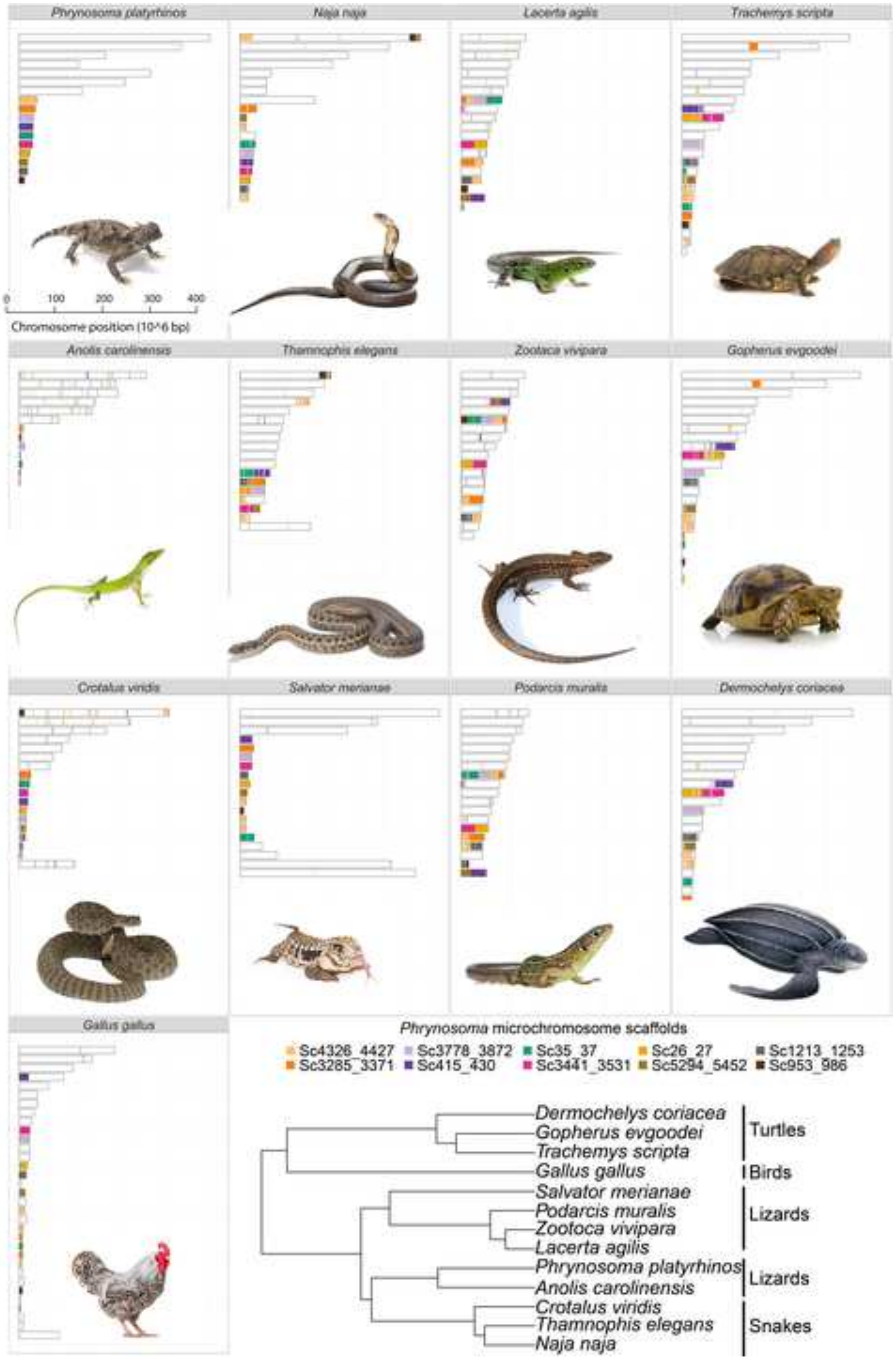

FigurS1

Click here to access/download;Figure;FigureS1.jpg

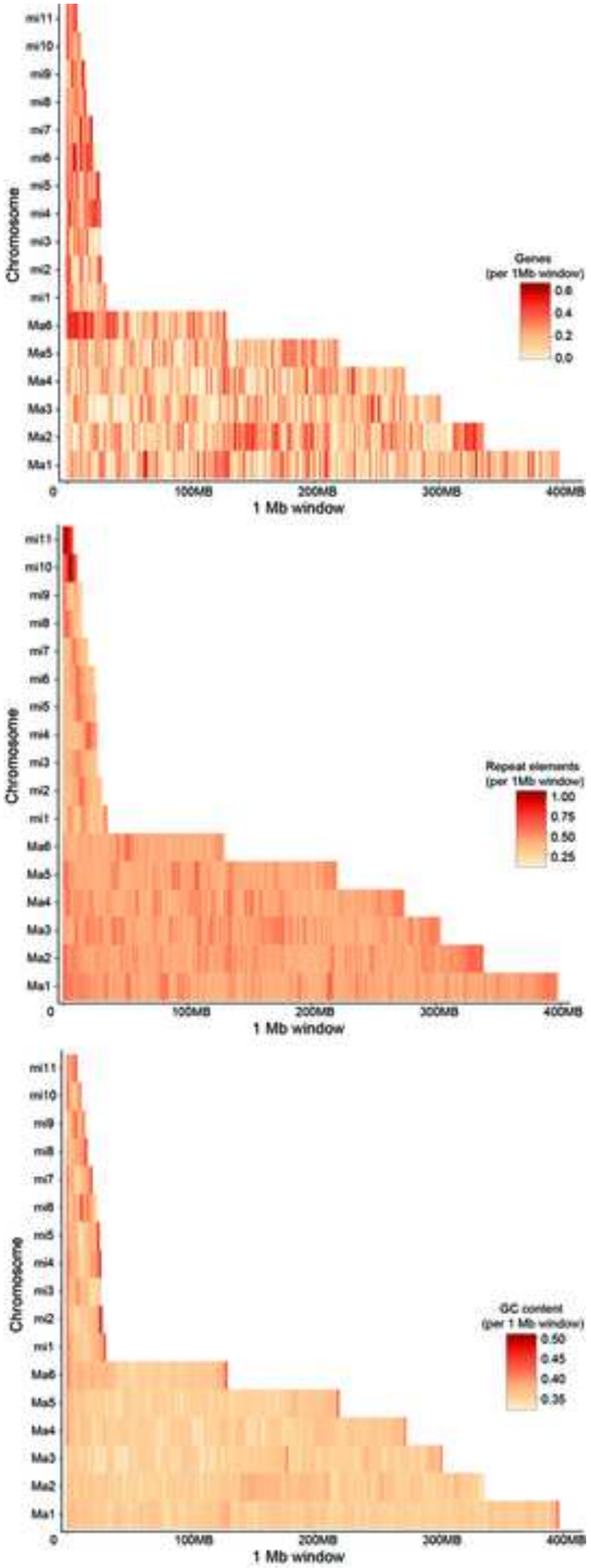

FigurS2a

Click here to access/download;Figure;FigureS2a.jpg

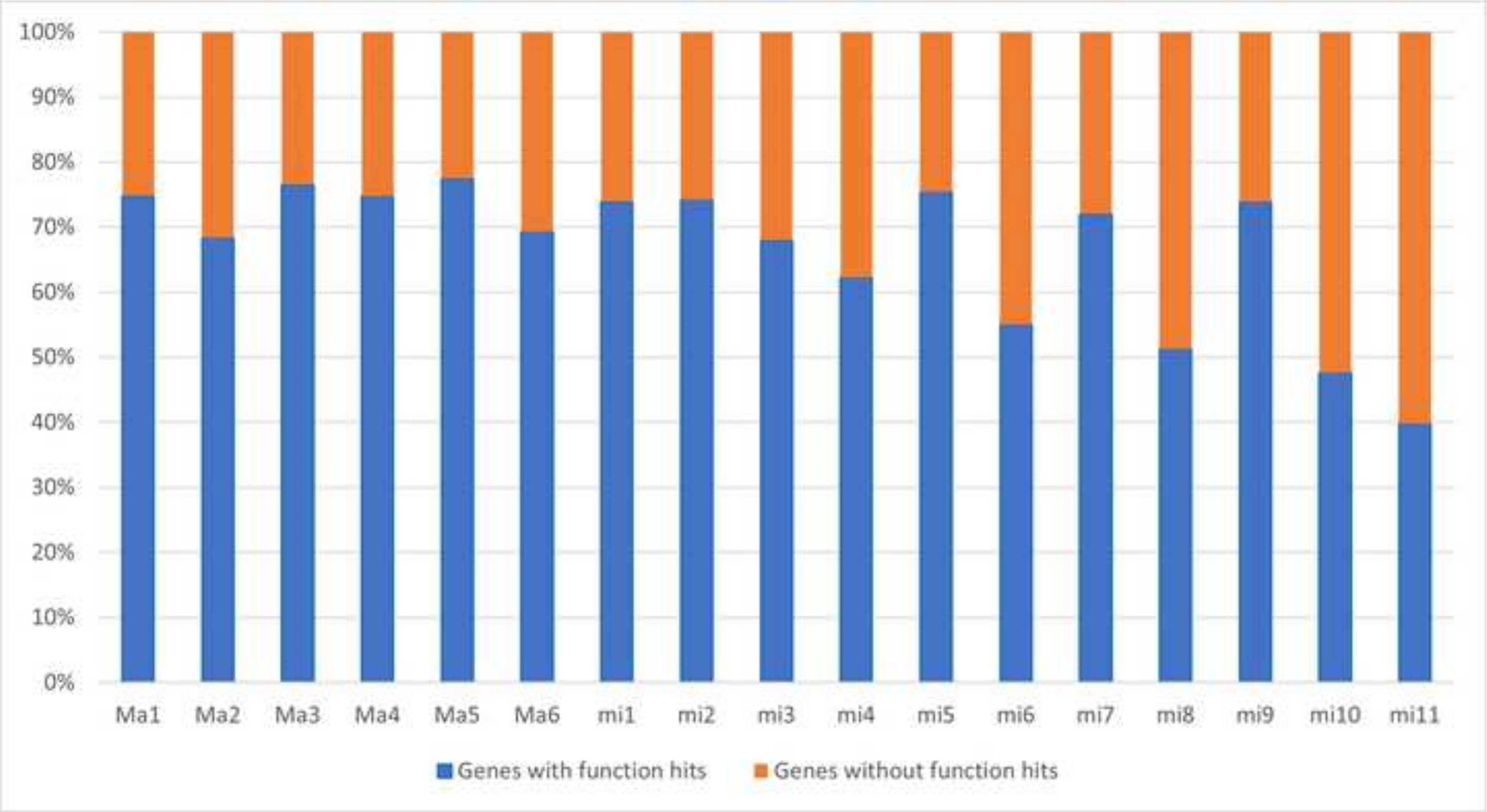

Click here to access/download;Figure;FigureS2b.jpg

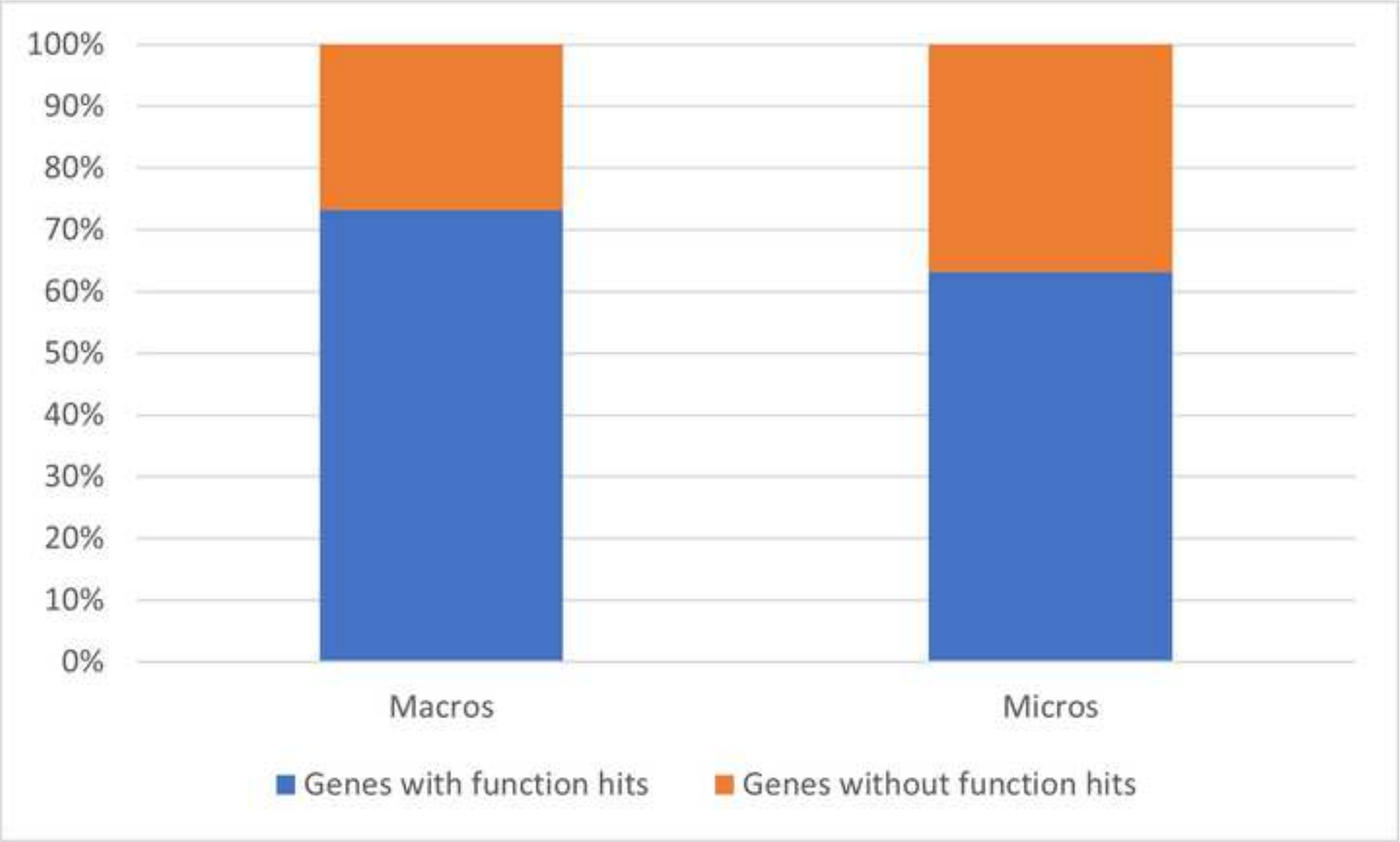

Click here to access/download;Figure;FigureS3.jpg

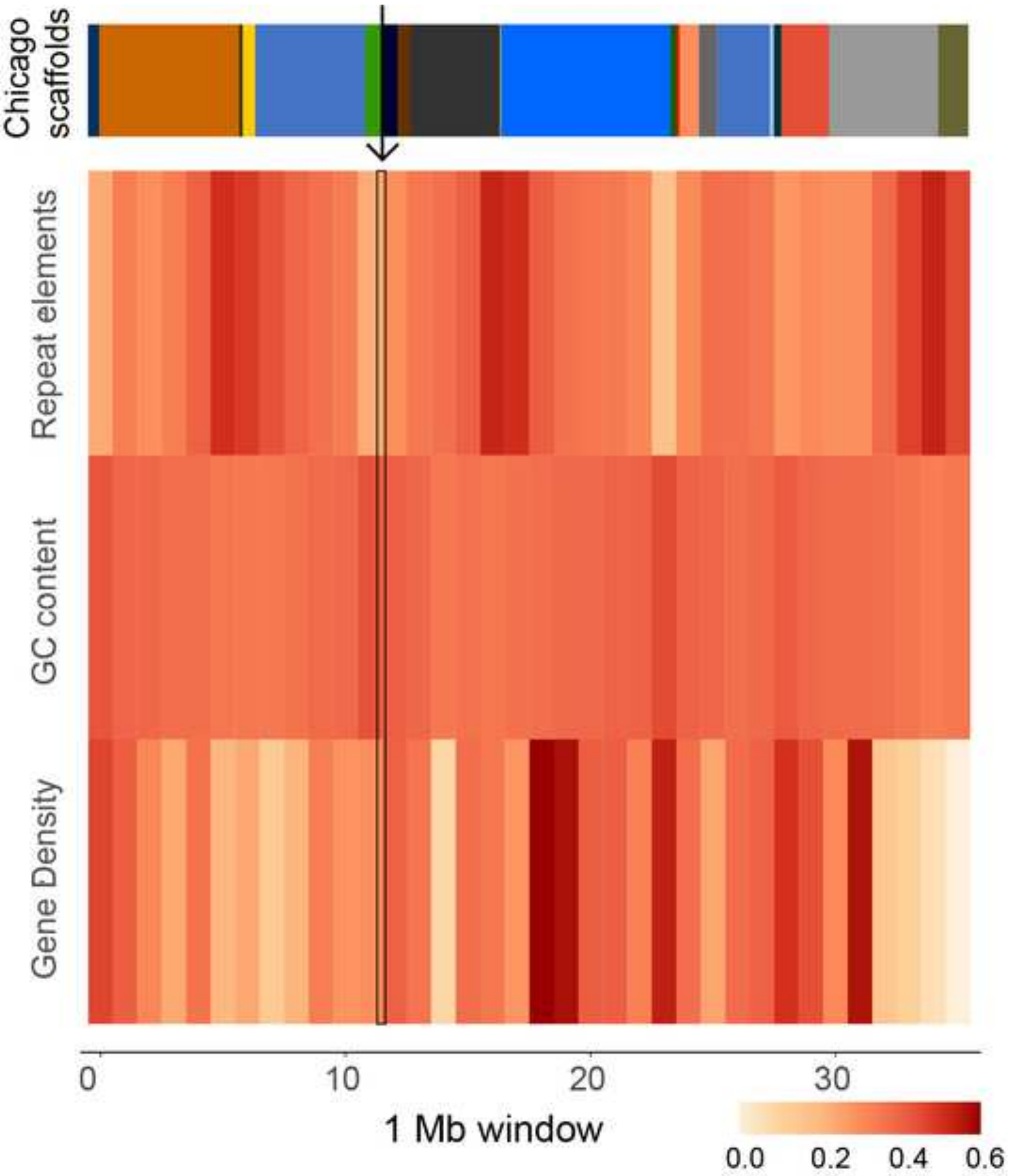

FigurS4 Click here to access/download;Figure;FigureS4.jpg

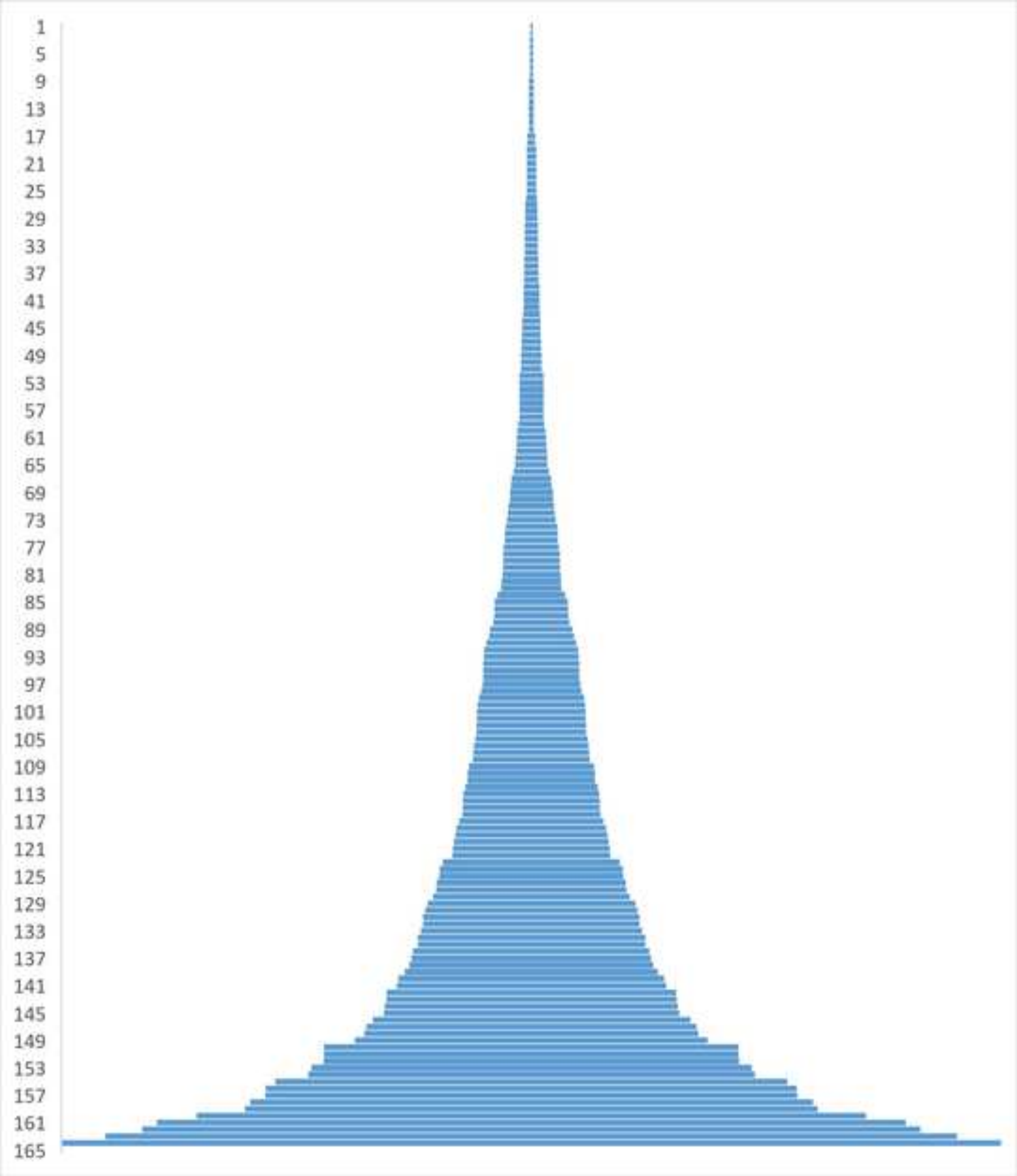

Supplement: giab098_GIGA-D-21-00044_Revision_1 [file giab098_giga-d-21-00044_revision_1.pdf]
