# Supplementary material for: A chromosome-level genome assembly and annotation of the desert horned lizard, Phrynosoma platyrhinos, provides insight into chromosomal rearrangements among reptiles
Source: Gigascience. 2022 Feb 4;11:giab098. doi: 10.1093/gigascience/giab098 (PMC8848323; doi:10.1093/gigascience/giab098)
Supplement: giab098_Supplemental_Tables_and_Figures [file giab098_supplemental_tables_and_figures.zip › giab098_Supplemental_Tables.docx]

Table S1. The corresponding scaffolds (first column) for each chromosome of P. platyrhinos (second column) and scaffold length (third column) in base pairs. *This scaffold was broken down into two microchromosomes (6 and 10).

| Scaffold name | Chromosome(s) name | length (in base pairs) |
| --- | --- | --- |
| Sc3291_377 | Chromosome 1 | 396,190,715 |
| Sc439 _455 | Chromosome 2 | 336,734,411 |
| Sc1234_1274 | Chromosome 3-a | 178,616,284 |
| Sc1882_1940 | Chromosome 3-b | 123,146,639 |
| Sc5292_5410 | Chromosome 4 | 273,212,746 |
| Sc5293_5450 | Chromosome 5 | 219,432,639 |
| Sc521_540 | Chromosome 6 | 129,273,435 |
| Sc3285_3371 | Microchromosome 1 | 31,685,405 |
| Sc3778_3872 | Microchromosome 2 | 28,086,253 |
| Sc415_430 | Microchromosome 3 | 27,277,973 |
| Sc35_37 | Microchromosome 4 | 27,087,043 |
| Sc3441_3531 | Microchromosome 5 | 26,097,904 |
| Sc4326_4427*  Sc4326a4427  Sc4326b4427 | Microchromosome 10 microchromosome 6 | 11,894,615  23,702,528 |
| Sc26_27 | Microchromosome 7 | 20,466,995 |
| Sc5294_5452 | Microchromosome 8 | 16,009,790 |
| Sc1213_1253 | Microchromosome 9/X | 15,721,303 |
| Sc953_986 | Microchromosome 11 | 8,897,685 |

Table S2. Best blast hits of cDNA [29] and * indicates sex linked markers [30] from *A. carolinensis* and *L. reevesii*  against the genome of *P. platyrhinos*.

| Marker | Accession | Chromosomal location | | | E-value |
| --- | --- | --- | --- | --- | --- |
|  |  | *A. carolinensis* | *L. reevesii* | *P. platyrhinos* |  |
| DYNC1H1 | AB490348 |  | 1q | Chr1 | 2.95E-179 |
| ESR1 | AB490345 |  | 1p | Chr1 | 1.02E-113 |
| WT1 | XM_016992885 | 1 |  | Chr1 | 2.19E-158 |
| WT1 | AB490347 |  | 1q | Chr1 | 7.53E-80 |
| XAB1 | AB490344 |  | 1p | Chr1 | 2.31E-35 |
| CHD1 | XM_008103079 | 2 |  | Chr2 | 0 |
| CHD1 | AB480289 |  | 2p | Chr2 | 1.25E-144 |
| DMRT1 | XM_003216553 | 2 |  | Chr2 | 0 |
| DMRT1 | AB480288 |  | 2p | Chr2 | 2.15E-64 |
| GHR | XM_008102837 | 2 |  | Chr2 | 0 |
| GHR | AB480290 |  | 2p | Chr2 | 1.01E-104 |
| RPS6 | XM_003216606 | 2 |  | Chr2 | 5.32E-123 |
| RPS6 | AB480287 |  | 2p | Chr2 | 2.39E-88 |
| RUFY1 | XM_008104854 | 2 |  | Chr2 | 0 |
| RUFY1 | AB490352 |  | 2q | Chr2 | 3.45E-22 |
| EIF2S3 | XM_003218845 | 3 |  | Chr3-a | 0 |
| EIF2S3 | AB490361 |  | 3q | Chr3-a | 5.58E-104 |
| OCA2 | XM_008107106 | 3 |  | Chr3-a | 0 |
| OCA2 | AB490360 |  | 3q | Chr3-a | 1.78E-89 |
| SH3PXD2A | XM_016992171 | 3 |  | Chr3-b | 0 |
| SH3PXD2A | AB490356 |  | 3p | Chr3-b | 5.98E-166 |
| TLOC1 | AB490355 | 3p |  | Chr3-b | 1.71E-79 |
| HDAC3 | XM_003219886 | 4 |  | Chr4 | 0 |
| HDAC3 | AB490365 |  | 4p | Chr4 | 4.16E-97 |
| RBM12 | XM_008109953 | 4 |  | Chr4 | 0 |
| RBM12 | AB490367 |  | 4q | Chr4 | 3.92E-137 |
| SS18 | XM_003219645 | 4 |  | Chr4 | 0 |
| SS18 | AB490397 |  | 4p | Chr4 | 1.75E-70 |
| ZNF326 | XM_008109275 | 4 |  | Chr4 | 0 |
| ZNF326 | AB490366 |  | 4q | Chr4 | 1.00E-128 |
| ACSL1 | XM_008111814 | 5 |  | Chr5 | 0 |
| ACSL1 | AB490370 |  | 5p | Chr5 | 1.00E-95 |
| DCLK2 | XM_008111991 | 5 |  | Chr5 | 0 |
| DCLK2 | AB490369 |  | 5p | Chr5 | 2.06E-73 |
| EXOC1 | XM_008111693 | 5 |  | Chr5 | 0 |
| EXOC1 | AB490371 |  | 5p | Chr5 | 3.08E-176 |
| RANGAP1 | XM_008110743 | 5 |  | Chr5 | 0 |
| RANGAP1 | AB490374 |  | 5q | Chr5 | 6.70E-80 |
| SOX5 | XM_008110345 | 5 |  | Chr5 | 0 |
| SOX5 | AB490376 |  | 5q | Chr5 | 1.78E-104 |
| UCHL1 | XM_003221541 | 5 |  | Chr5 | 2.55E-63 |
| UCHL1 | AB490372 |  | 5p | Chr5 | 3.46E-59 |
| CTNNB1 | AB490379 |  | 6q | Chr6 | 0 |
| GAD2 | XM_003222133 | 6 |  | Chr6 | 0 |
| GAD2 | AB490380 |  | 6q | Chr6 | 1.98E-76 |
| MYST2 | AB490378 |  | 6p | Chr6 | 0 |
| WAC | XM_008112381 | 6 |  | Chr6 | 0 |
| WAC | AB490381 |  | 6q | Chr6 | 3.60E-159 |
| AR | AB490385 |  | micro | microchr3 | 2.72E-152 |
| TMEM132D* | XM_008113640.2 | micro “b”/X |  | microchr9/X | 0 |
| FZD10* | XM_003222753.3 | micro “b”/X |  | microchr9/X | 0 |
| ATP2A2* | XM_008113715 | micro “b”/X |  | microchr9/X | 0 |
| ATP2A2 | AB490391 |  | micro | microchr9/X | 4.05E-167 |
| ATRX | AB490386 |  | micro | microchr3 | 7.88E-127 |
| BRD7 | AB490390 |  | micro | microchr2 | 3.95E-68 |
| HSPA8 | XM_003222794 | micro “a” |  | Chr1 | 0 |
| HSPA8 | AB490395 |  | micro | microchr4 | 3.70E-162 |

Table S3. Number, length, and percentage of annotated repeat elements identified.

| Families of repeat elements | Numbers of elements | Length masked (bp) | % of sequence | % element masked |
| --- | --- | --- | --- | --- |
| **Retroelements** | 2,082,017 | 451,287,018 | 23.83 | 20.37 |
| **SINEs** | 648,720 | 89,280,596 | 4.72 | 6.35 |
| Penelope | 254,722 | 35,799,757 | 1.89 | 2.50 |
| **LINEs** | 1,311,944 | 319,965,632 | 16.90 | 12.84 |
| L2/CR1/Rex | 702,907 | 160,952,766 | 8.50 | 6.88 |
| R1/LOA/Jockey | 36 | 3,068 | 0.00 | 0.00 |
| R2/R4/NeSL | 5,129 | 640,551 | 0.03 | 0.05 |
| RTE/Bov-B | 257,696 | 83,172,778 | 4.39 | 2.52 |
| L1/CIN4 | 87,958 | 38,708,200 | 2.04 | 0.86 |
| **LTR elements** | 121,353 | 42,040,790 | 2.22 | 1.19 |
| BEL/Pao | 4,074 | 768,559 | 0.04 | 0.04 |
| Ty1/Copia | 18,376 | 7,918,963 | 0.42 | 0.18 |
| Gypsy/DIRS1 | 39,227 | 14,661,509 | 0.77 | 0.38 |
| Retroviral | 34,521 | 5,663,234 | 0.30 | 0.34 |
| **DNA transposons** | 1,527,111 | 204,435,133 | 10.80 | 14.94 |
| hobo-Activator | 610,832 | 73,847,731 | 3.90 | 5.98 |
| Tc1-IS630-Pogo | 314,462 | 42,728,561 | 2.26 | 3.08 |
| PiggyBac | 1,795 | 445,424 | 0.02 | 0.02 |
| Tourist/Harbinger | 500,329 | 78,020,620 | 4.12 | 4.90 |
| **Unclassified** | 828,472 | 146,176,330 | 7.72 | 8.11 |
| **Total interspersed repeats** | 9,351,681 | 801,898,481 | 42.35 | 91.51 |
| Small RNA | 33,490 | 3,376,969 | 0.18 | 0.33 |
| Satellites | 51,860 | 7,242,936 | 0.38 | 0.51 |
| Simple repeats | 705,413 | 27,116,672 | 1.43 | 6.90 |
| Low complexity | 77,452 | 3,957,871 | 0.21 | 0.76 |
| **Total masked** | 10,219,896 | 841,750,763 | 44.45 | 100.00 |

Table S4: Comparison of molecular pathways analysis on macrochromosomes and microchromosomes. Second column shows the specific pathways identified on each chromosome. Third column shows the pathways that belong to specific group of chromosomes.

| Chromosome location | Specific pathways for each chromosome | Specific pathways for macros versus micros |
| --- | --- | --- |
| Chromosome 1 | Allantoin degradation (P02725), Methionine biosynthesis (P02753) | 5-Hydroxytryptamine biosynthesis (P04371), Acetate utilization (P02722), Activin beta signaling pathway (P06210), Anandamide degradation (P05728), Androgen/estrogene/progesterone biosynthesis (P02727), Ascorbate degradation (P02729), ATP synthesis (P02721), Biotin biosynthesis (P02731), BMP/activin signaling pathway-drosophila (P06211), DPP signaling pathway (P06213), DPP-SCW signaling pathway (P06212), Glutamine glutamate conversion (P02745), Isoleucine biosynthesis (P02748), Leucine biosynthesis (P02749), Methylmalonyl pathway (P02755), Proline biosynthesis (P02768), Purine metabolism (P02769), Pyridoxal phosphate salvage pathway (P02770), Pyridoxal-5-phosphate biosynthesis (P02759), SCW signaling pathway (P06216), Succinate to proprionate conversion (P02777), Toll pathway-drosophila (P06217), Valine biosynthesis (P02785), and Vitamin B6 metabolism (P02787) |
| Chromosome 2 | ALP23B signaling pathway (P06209), GBB signaling pathway (P06214), MYO signaling pathway (P06215) |  |
| Chromosome 3 | Cysteine biosynthesis (P02737), Lysine biosynthesis (P02751) |  |
| Chromosome 4 | Thiamin metabolism (P02780) |  |
| Chromosome 5 | Cobalamin biosynthesis (P02735), Sulfate assimilation (P02778) |  |
| Chromosome 6 | Carnitine metabolism (P02733), Coenzyme A linked carnitine metabolism (P02732), and Threonine biosynthesis (P02781) |  |
| Microchromosome 1 | None. | None. |
| Microchromosome 2 | Tyrosine biosynthesis (P02784) |  |
| Microchromosome 3 | None. |  |
| Microchromosome 4 | Bupropion degradation (P05729) |  |
| Microchromosome 5 | Triacylglycerol metabolism (P02782) |  |
| Microchromosome 6 | None. |  |
| Microchromosome 7 | None. |  |
| Microchromosome 8 | None. |  |
| Microchromosome 9/X | None. |  |
| Microchromosome10 | None |  |
| Microchromosome 11 | None. |  |

Table S5. Genome assemblies and number of markers used for *in silico* painting. All assemblies are available through NCBI under the appropriate accession.

| Organism | Potential single markers | Total confirmed (5 consecutive) markers | Scaffolds with confirmed homologies | Confirmed markers in Scaffolds (%) | Assembly accession |
| --- | --- | --- | --- | --- | --- |
| *A. carolinensis* | 2,616,045 | 87,155 | 13 | 57,006  (65.41) | GCA_000090745.2 |
| *S. merianae* | 390,847 | 31,955 | 19 | 31,805  (99.53) | GCA_003586115.2 |
| *L. agilis* | 755,639 | 44,200 | 20 | 44,199  (99.99) | GCA_009819535.1 |
| *P. muralis* | 719,822 | 46,093 | 19 | 45,731  (99.21) | GCA_004329235.1 |
| *Z. vivipara* | 751,121 | 43,371 | 19 | 42,224  (97.35) | GCA_011800845.1 |
| *C. viridis* | 299,173 | 18,161 | 18 | 17,891  (98.51) | GCA_003400415.2 |
| *T. elegans* | 282,458 | 17,817 | 18 | 17,725  (99.48) | GCA_009769535.1 |
| *N. naja* | 291, 209 | 19,898 | 19 | 19,805  (99.52) | GCA_009733165.1 |
| *T. scripta* | 177,241 | 15,287 | 25 | 15,252  (99.77) | GCA_013100865.1 |
| *G. evgoodei* | 152,748 | 14,864 | 24 | 14,614  (98.32) | GCA_007399415.1 |
| *D. coriacea* | 137,161 | 14,075 | 29 | 14,075  (100.00) | GCA_009764565.3 |
| *G. gallus* | 88,397 | 10,934 | 33 | 10,934  (100.00) | GCA_000002315.5 |
