# Supplementary figures and images for: A chromosome-level genome assembly and annotation of the desert horned lizard, Phrynosoma platyrhinos, provides insight into chromosomal rearrangements among reptiles
Source: Gigascience. 2022 Feb 4;11:giab098. doi: 10.1093/gigascience/giab098 (PMC8848323; doi:10.1093/gigascience/giab098)

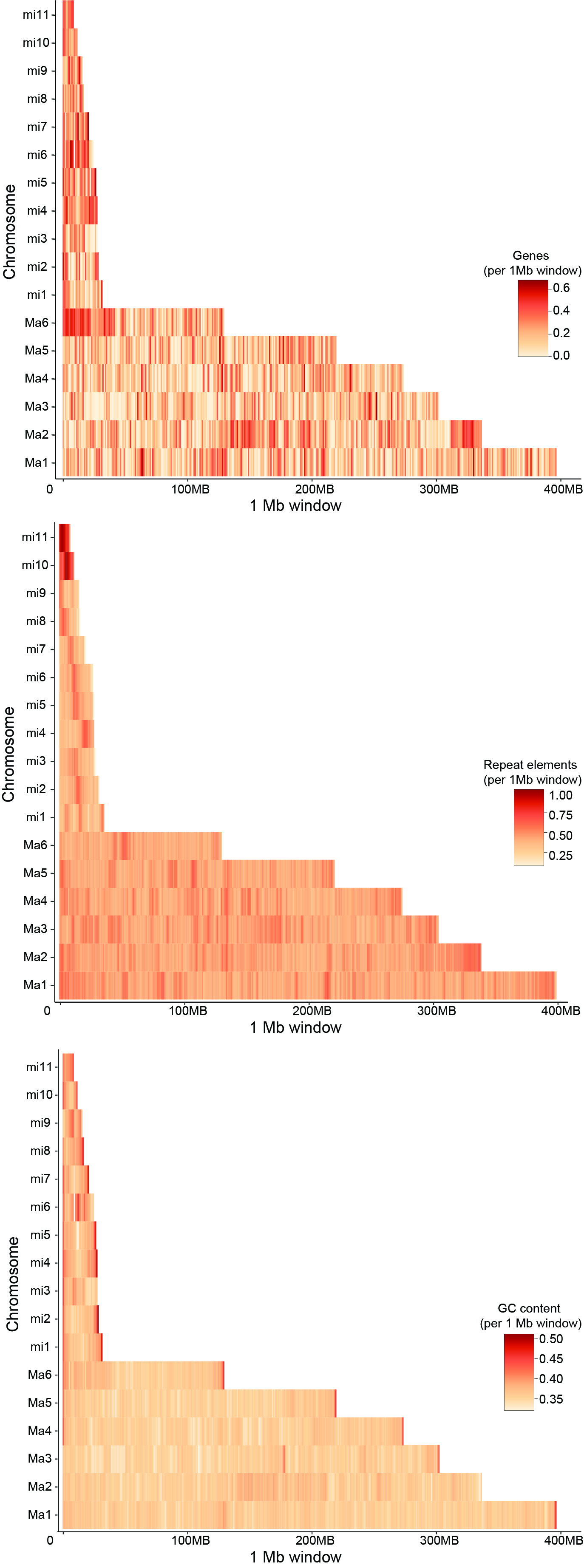

Supplement: giab098_Supplemental_Tables_and_Figures [file giab098_supplemental_tables_and_figures.zip › FigureS1.jpg]

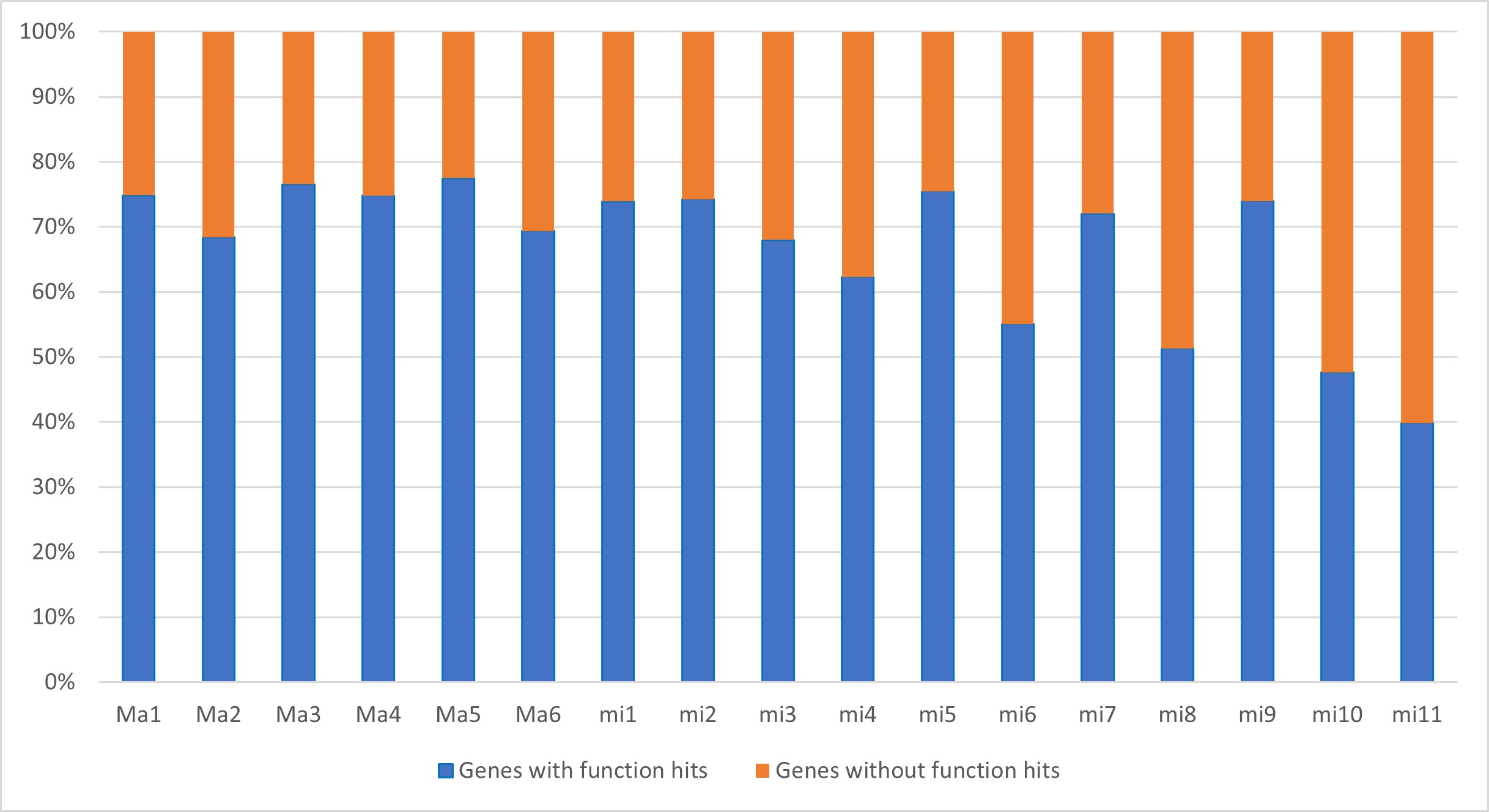

Supplement: giab098_Supplemental_Tables_and_Figures [file giab098_supplemental_tables_and_figures.zip › FigureS2a.jpg]

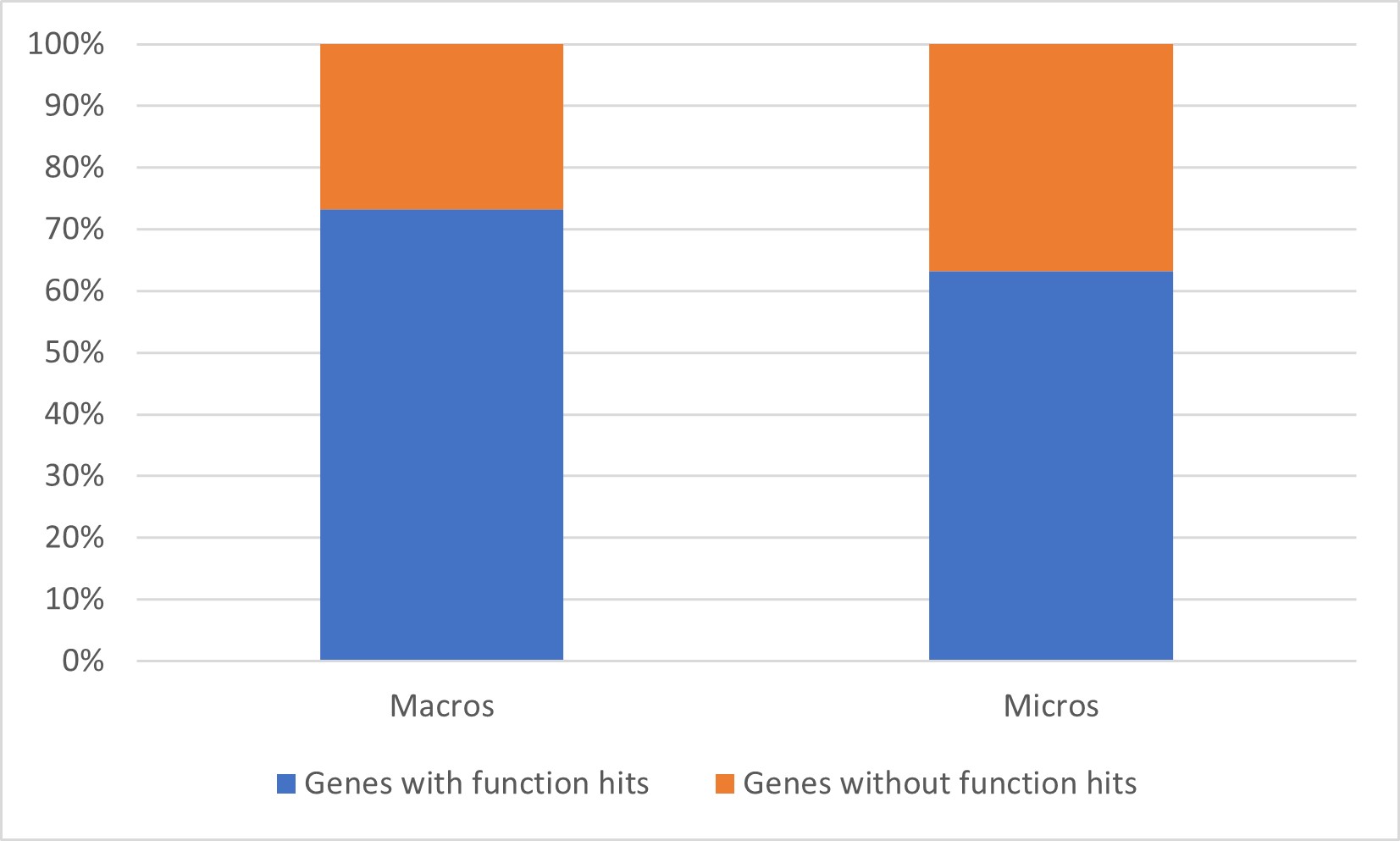

Supplement: giab098_Supplemental_Tables_and_Figures [file giab098_supplemental_tables_and_figures.zip › FigureS2b.jpg]

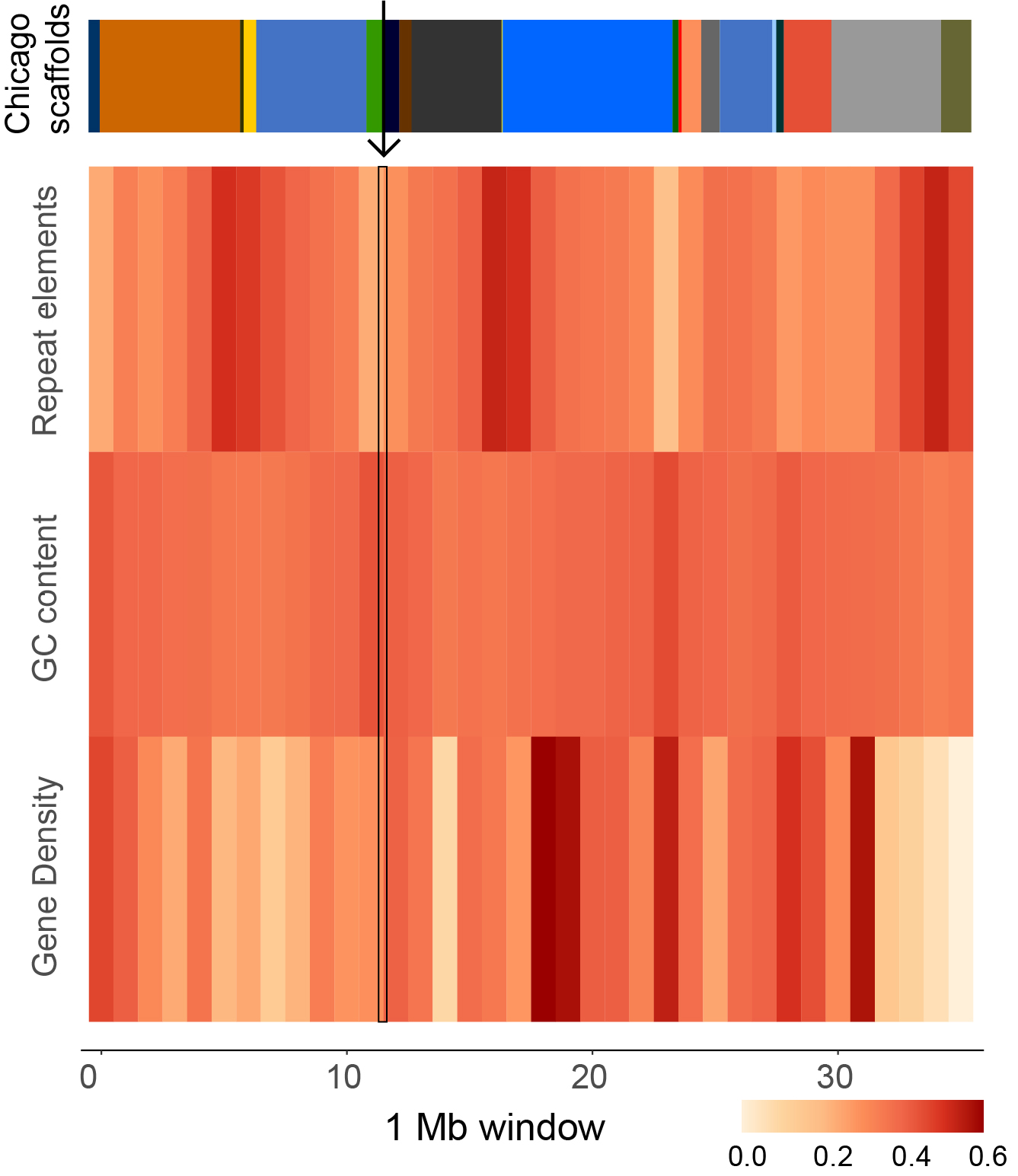

Supplement: giab098_Supplemental_Tables_and_Figures [file giab098_supplemental_tables_and_figures.zip › FigureS3.jpg]

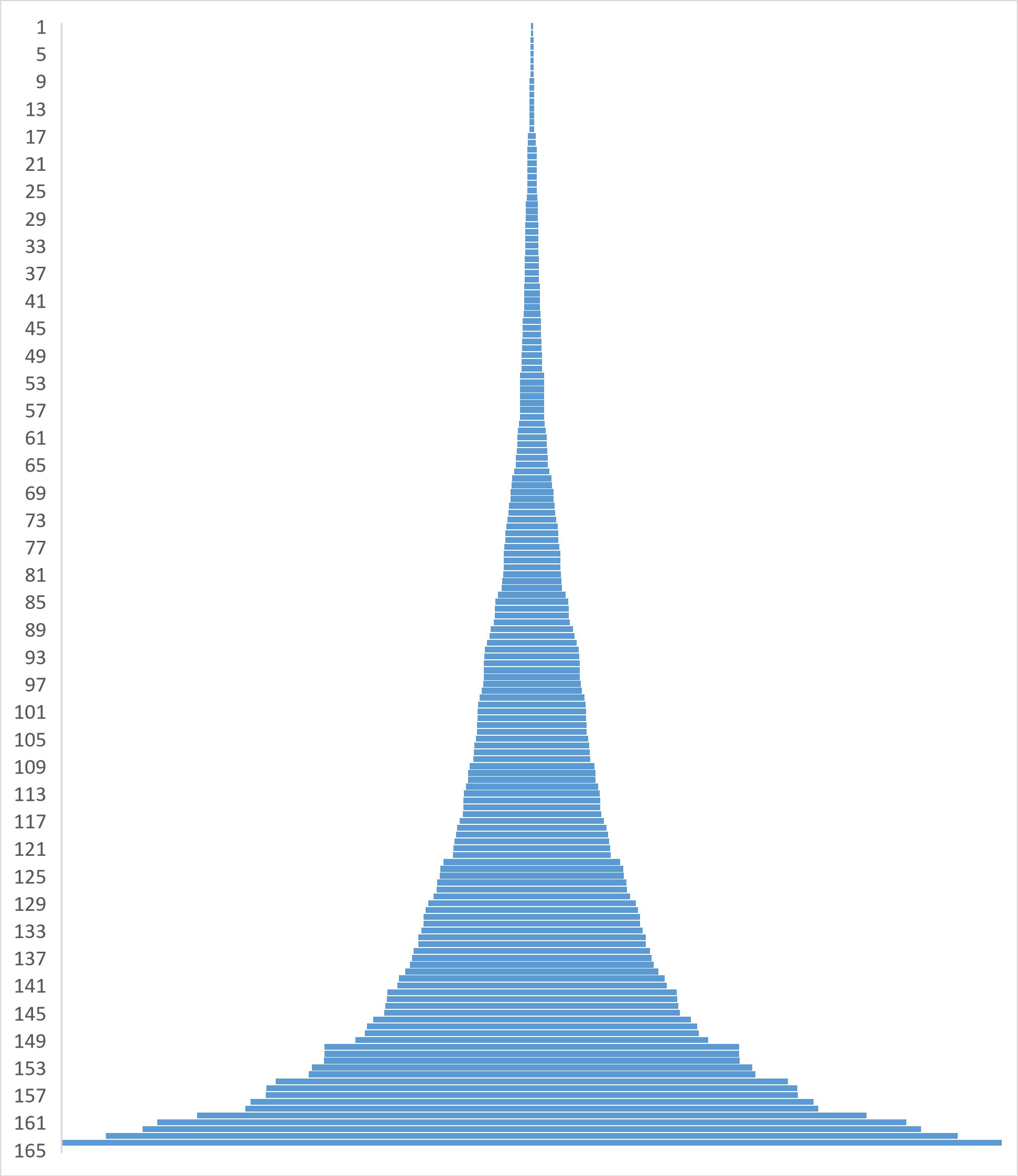

Supplement: giab098_Supplemental_Tables_and_Figures [file giab098_supplemental_tables_and_figures.zip › FigureS4.jpg]
