# Supplementary material for: A chromosome-level genome assembly and annotation of the desert horned lizard, Phrynosoma platyrhinos, provides insight into chromosomal rearrangements among reptiles
Source: Gigascience. 2022 Feb 4;11:giab098. doi: 10.1093/gigascience/giab098 (PMC8848323; doi:10.1093/gigascience/giab098)
Supplement: giab098_Response_to_Reviewer_Comments_Revision_1 [file giab098_response_to_reviewer_comments_revision_1.pdf]

# Author's Response To Reviewer Comments

Close

Point-By-Point Response to Reviews
Reviewer reports:
Reviewer #1: I want to thank authors for carefully considering all the comments raised by reviewers. This manuscript will substantially add to the genomic resources for further studies. Most importantly, micro chromosome work will indeed pave way to new research in understanding their nuanced biology and evolution. Great work and congratulations.
*** We are pleased that the reviewer was satisfied with our revisions and supportive of the foundational nature of the work on microchromosomes, and we thank them for their time and feedback.

Reviewer #2: The authors have done a good job with the revision. I only have a few points that I would like to emphasize and suggest they address further.
*** We are happy to hear that the reviewer thought that our previous revision was well done, and thank them for their additional feedback here.

1. In comparison to BUSCO results from other recent squamate genomes (see Figure 2d in https://www.biorxiv.org/content/10.1101/2021.09.28.462146v1.full for a comparison) the BUSCO results from this genome assembly suggest it is not considerably high quality assembly (relatively). The 16% missing and only 64% complete BUSCOs suggest this assembly is relatively incomplete and is a lower quality assembly (more fragmented) than many of the other squamate genomes. This should be explained and discussed in the text, particularly in how this may affect their conclusions about defining the microchromosomes, their synteny analysis, and their pathway analysis.
*** In the discussion we now mention the possible effect of the incomplete assembly on our results (lines 234-239, and 284-286)

2. It should be further emphasized throughout that the microchromosome naming designations are putative (and very possibly may not correspond to the karyotype) due to (1) the incomplete nature of the assembly, (2) the splitting of scaffolds into microchromosomes based on bioinformatic predictions, (3) and their naming based on length when some of their lengths are very similar (many less then 200,000 bp difference, Table S1).
*** We added this as suggested to the discussion (lines 234-239).

3. Line 181, I think you are missing a number for the upper limit. To define the 14 scaffolds that are > 6mb and < #?
*** The upper limit for these 14 scaffolds now been added (75 Mb, line 181).

4. Line 193, I think 'm' is supposed to be a number here.
*** The index is explained a couple lines above (line 190). In this case, “m” is a variable based on the species chromosome number.

Close
