# Supplementary material for: A chromosome-level genome assembly and annotation of the desert horned lizard, Phrynosoma platyrhinos, provides insight into chromosomal rearrangements among reptiles
Source: Gigascience. 2022 Feb 4;11:giab098. doi: 10.1093/gigascience/giab098 (PMC8848323; doi:10.1093/gigascience/giab098)
Supplement: giab098_Reviewer_1_Report_Original_Submission — Hardip Patel -- 4/22/2021 Reviewed [file giab098_reviewer_1_report_original_submission.pdf]

Reviewer Report

Title: A chromosome-level genome assembly and annotation of the desert horned lizard, Phrynosoma platyrhinos, provides insight into chromosomal rearrangements among reptiles

Version: Original Submission **Date:** 4/22/2021

Reviewer name: Hardip Patel

Reviewer Comments to Author:

Authors present the chromosome level genome assembly of the desert horned lizard. They have used Chicago and HiC libraries to construct the genome assembly, which is used for exploratory analyses of chromosomal conservation patterns and describe properties of microchromosomes compositions (repeat, gene and GC%). The manuscript requires substantial revision and report plenty of details that are missing in methods section.

General comments:

I had difficulty following the manuscript given the substantial number of technical details were lacking. Broadly, large number of conclusions seemed to be derived from visual observations of figures and graphs without quantitative analyses to back those claims. I also noticed that there were sections in discussions that could be transferred to results (analyses) section. I also found repetitive content in discussions and analyses section. Overall, the manuscript writing style was not up to the level expected of a scientific descriptive paper. Major revision in style of writing is essential to ensure completeness and accuracy of information for readers.

Karyotype for the desert horned lizard is assumed from previous study, but not substantiated. Generally, it is OK. However, at least mentioning this in discussion and how those assumptions can have implications in understanding homology are not discussed. Similarly, the quality of the assembly is not verified by any orthogonal method and therefore some of the claims in the manuscript may be wrong. I would encourage authors to discuss their results in the context of the quality of the assembly.

One of the major points I would like to raise is about the use of genome assemblies that Vertebrate Genome Project (Genome 10K) have generated for this kind of global analyses work. They have an embargo on the use of data as per documentation at https://genome10k.soe.ucsc.edu/data-use-policies/. First, I would like to state that, I am not a member of any of the Genome10K, VGP or other associated projects.

The embargo on data use is protected by the Fort Lauderdale Agreement (https://www.genome.gov/Pages/Research/WellcomeReport0303.pdf). Please refer to the page 4, section C. "Resource Users" (points 2 and 3) as a shared responsibility by end users of the data. I personally prefer that the Fort Lauderdale Agreement be revised in the interest of research because embargo periods are substantially larger compared to the pace of data production these days. However, we collectively as scientists need to bring this change together in the interest of advancing science fairly for all. I leave it up to authors, journal and the editors to make the judgement call on the use of data that is under embargo.

Specific comments:

Below I provide a list of specific comments.

Title: Please change common name to lower case.

Introduction:

1. Page 3, Line 59-60: Either use average for both or range for both to be consistent
2. Line 77: Convention is to say "ZW sex chromosome". Perhaps authors can change if they wish.

Analysis:

1. Line 109: Mean 0.18. The metric for gene density is unclear. Is it per Mb, or per 100Kb? Also, you have GC on the same fractional scale. Perhaps best to report GC as percentage as it is the widely accepted unit and gene content per Mb (or some other fixed scale). Same is the case for repeat content. Please specify the scales appropriately. Repeats being variable in size as units, perhaps report it as a proportion of the "chromosome" length.
2. Line 111:112: "elements identified 44.5%" is unclear. Is it that of all repeats, 44.5% repeats were identified and rest 55.5% missing from annotation? Or alternately and probably the case that 44.5% of the genome is composed of repeat elements.
3. Line 113: If repeat content is compared for macro vs micro, the results don't seem to be significant considering the SD of 0.056 for the macro. 0.45 - 3*0.056 is 0.28, lower than the 0.39 for micro. Differences are not significant I guess on this scale.
4. GO analyses is not performed using statistics. Mere assignments to GO terms is a stretch. Statistical test is not listed. Perhaps authors should list the test.
5. Line 178 - 181: Please provide information about what chromosome numbers are you talking about here. It is very difficult to read figures with large number of chromosomes and colors.

Discussion:

1. Line 211: gBGC acronym is used only once. Please remove it.
2. Figure 2: What is the black line for in the inner circle? Circos plots are pleasant to look at but don't convey the message using the heatmaps in this figure. Perhaps authors may consider redrawing this figure with a line plot using karyoploteR package in R (https://www.bioconductor.org/packages/release/bioc/html/karyoploteR.html).
3. Line 214: I had difficulty in observing higher GC content pattern in subtelomeric regions. Authors must provide statistical calculations to show if the pattern is statistically significant or not. Otherwise, they should remove the reference to this discussion point.
4. Line 218 - 225 should be migrated to the analysis section.
5. Line 236: regarding "several microchromosomes", please provide specific chromosome numbers and perhaps think about moving factual informationyou're your observations of the data into result section.

Methods:

1. Line 284: Ethics approval for the male for transcriptome sequencing is not mentioned. Please list it.
2. Methods are very light on data and assembly generation. This requires major effort. Please see https://academic.oup.com/g3journal/article/10/4/1159/6026169 for an example of how this section should be described. DNA extraction method, fragment size for library prep, read lengths targeted, paired vs single end mode for sequencing, sequencing platform (x10, hiseq2500, novaseq), library method in details.
3. Genome and transcriptome assembly section: Methods are not very clear. It is mentioned that HiRise Scaffolding pipeline was used. No reference, no command line settings, availability of the software is

listed.
4. Line 293: Karyotype information is not clear. The paper cited states, "The ancestral 2n = 34 (12M + 20m + XY) phrynosomatid karyotype that is found in several of the basal lineages of Sceloporus differs from the standard iguanian karyotype by the loss of a single pair of microchromosomes.
This to me suggests a karyotype of 6 macro and 10 micro-chromosomes including the sex chromosomes. Also, the main thesis of the work is about variable karyotype configurations in reptiles. It would be good if authors discussed accuracy of this information. If karyotype cannot be produced, then authors can at least discuss this limitation.
5. Line 296: "Best BLAST" needs to be elaborated clearly with version numbers of assemblies and annotations used for such analyses. Details of software and parameter settings need to be described.
6. Line 303: S. Merianae genome is used as a source of truth. However, that genome is fragmented as well with 4512 scaffolds. Therefore, the statement in the next line "For example, in S. merianae, three microchromosome account for this scaffold" cannot be robust. This leads to the fact that lines 306-308 cannot be trusted. If authors insist on splitting automatically generated scaffolds using manual curation, then the curation should be applied consistently across the genome and not at handpicked locations. This causes confusion for downstream use of the genome reference.
7. Line 310: How was this performed?
8. Line 316: Replace "unknown" with "novel".
9. Line 349: The RBB pipeline is nowhere described at the link provided as reference. The link only provides information about how to create annotation tracks. Please detail methods clearly.
10. Gene ontology section: Needs more detail about the software version, parameters, commands, and essential thresholds used to determine significance of enrichment or depletion.
11. Line 361-362: Please describe the command used to calculate GC content, gene density, repeat elements etc. What were the sources of these annotations to be used with markwindows tool?
12. Line 363 - 367: Please list assembly version for posterity.
13. Line 368: reference for painting method appears to be incorrect. Please provide accurate reference for the in-silico painting method.
14. Painting method requires substantial addition in how the BLAST was performed. What was the tool (blastn, megablast, dcmegablast) used? If default parameters used, then say so.
References:
1. Page, issue and volume numbers are present in some but not all references.
2. Remove letters next to the year. Perhaps something to do with the reference manager.
3. Reference 2, 11, 14, 20, 54 are examples of references without complete list of authors. Use consistent style.
4. Reference 18 has a typo for the species name. Please correct it.

Methods

Are the methods appropriate to the aims of the study, are they well described, and are necessary controls included? Choose an item.

Conclusions

Are the conclusions adequately supported by the data shown? Choose an item.

Reporting Standards

Does the manuscript adhere to the journal's guidelines on minimum standards of reporting? Choose an item.

Choose an item.

Statistics

Are you able to assess all statistics in the manuscript, including the appropriateness of statistical tests used? Choose an item.

Quality of Written English

Please indicate the quality of language in the manuscript: Choose an item.

Declaration of Competing Interests

Please complete a declaration of competing interests, considering the following questions:

- Have you in the past five years received reimbursements, fees, funding, or salary from an organisation that may in any way gain or lose financially from the publication of this manuscript, either now or in the future?
- Do you hold any stocks or shares in an organisation that may in any way gain or lose financially from the publication of this manuscript, either now or in the future?
- Do you hold or are you currently applying for any patents relating to the content of the manuscript?
- Have you received reimbursements, fees, funding, or salary from an organization that holds or has applied for patents relating to the content of the manuscript?
- Do you have any other financial competing interests?
- Do you have any non-financial competing interests in relation to this paper?

If you can answer no to all of the above, write 'I declare that I have no competing interests' below. If your reply is yes to any, please give details below.

I declare that I have no competing interests

I agree to the open peer review policy of the journal. I understand that my name will be included on my report to the authors and, if the manuscript is accepted for publication, my named report including any attachments I upload will be posted on the website along with the authors' responses. I agree for my report to be made available under an Open Access Creative Commons CC-BY license (http://creativecommons.org/licenses/by/4.0/). I understand that any comments which I do not wish to

be included in my named report can be included as confidential comments to the editors, which will not be published.

Choose an item.

To further support our reviewers, we have joined with Publons, where you can gain additional credit to further highlight your hard work (see: https://publons.com/journal/530/gigascience). On publication of this paper, your review will be automatically added to Publons, you can then choose whether or not to claim your Publons credit. I understand this statement.

Yes Choose an item.
