# Supplementary material for: A chromosome-level genome assembly and annotation of the desert horned lizard, Phrynosoma platyrhinos, provides insight into chromosomal rearrangements among reptiles
Source: Gigascience. 2022 Feb 4;11:giab098. doi: 10.1093/gigascience/giab098 (PMC8848323; doi:10.1093/gigascience/giab098)
Supplement: giab098_Reviewer_2_Report_Original_Submission — Tonia Schwartz -- 5/11/2021 Reviewed [file giab098_reviewer_2_report_original_submission.pdf]

Reviewer Report

Title: A chromosome-level genome assembly and annotation of the desert horned lizard, Phrynosoma platyrhinos, provides insight into chromosomal rearrangements among reptiles

Version: Original Submission **Date:** 5/11/2021

Reviewer name: Tonia Schwartz

Reviewer Comments to Author:

This is a review of the manuscript entitled "A chromosome-level genome assembly and annotation of the Desert Horned Lizard, Phrynosoma platyrhinos, provides insight into chromosomal rearrangements among reptiles". This manuscript presents the genome assembly and annotation of the desert horned lizard. Besides providing these resources the authors conduct some analyses that bring insight into micro and macrochromosime evolution. They indicate that gene density seems higher on the microchromosomes, and microchromosomes are more conserved as blocks based on the synteny analysis. The use of an ecological statistics is a clever way for assessing chromosomal dispersion. Overall I think this manuscript a very useful contribution to the field, however, a number of areas in this manuscript need to be clarified and perhaps reevaluated, as described below. I hope the authors find these useful for improving their manuscript.

Major Points:

1. In general, the methods section needs more details, and since the Analysis section is presented first it needs to be sufficient for understanding how you arrived at your findings without having read the methods that are at the end of the manuscript. In many sections I had to read the Methods section alongside the Analysis section to understand the Analysis section.

2. Page 13, methods for breaking scaffold 8.

a. Can you further explain or provide references for why high GC, and low repeats would indicate a good break point. This is not intuitive in the context of telomeres at the end of chromosomes having high repeats of AT rich regions.

b. For transparency, indicate on Figure 2 and Table 2 which microchromosomes derived from the scaffold 8 that was "broken".

c. Indicate on Supplemental Figure 1 where the scaffold was broken and label the ends with their respective microchromosome designations.

d. Why do you think these were put into the same scaffold? This information could be useful for others in trying to understand their assemblies

3. Scaffolds or Chromosomes: In Figure 2 it is confusing that the two scaffolds you think are chromosome 3 are separated, but scaffold 8 has been broken into the proposed macrochromosomes. I suggest you either use this figure to represent scaffolds with scaffolds for proposed chromosome 3a and 3b separated and the full scaffold 8 intact, OR you use this figure to represent proposed chromosomes with the two scaffolds representing proposed chromosome 3 together and the scaffold 8 broken to represent the proposed microcromosomes. Regardless, for transparency you should have both scaffold

and chromosome labels around the circle.
4. Gene Ontology. I think this is interesting but more details are needed on the GO Function analysis, and I suggest backing off on some conclusions or putting them in the context of the limitations of the study. For example, in the Annotation section it is mentioned that 20,764 protein coding genes were annotated, but in the gene ontology only ~11,000 (~1/2 were used). Why is this the case? Are they predicted proteins without gene "names". And then further only 7000 (1/3 of the annotated protein coding genes) were able to be assigned a molecular function.
a. If you are only able to use 1/3 of the annotated genes in your analysis, how confident are you in these results when most of the data are missing?
b. Is this 1/3 of genes that can be included in your analytical tests evenly distributed among the chromosomes? For example, are 1/3 of the annotated genes on chromosome 1 included in your test, and are 1/3 of the annotated genes on microchromosome 3 included in your test? OR are these proportions very different across the chromosomes? If they are different what bias does that introduce in this test?
c. What statistical model was used for testing for different molecular functions associated with the micro vs macrochromosomes? How many tests were completed: 8 level 1, 42 level 2, 142 level 3 = 192 statistical tests? Was a false discovery rate used in determining statistical significance?
d. Table S3 needs to have the adjusted P-values or FDR for statistical significance included as a column.
e. Clarify how the "activating / positive regulatory" and "repressive/negative regulatory" roles are being defined?
5. Page 6, Synteny analysis. It seems circular to say A. carolinensis had the same macrochromsome structure when you used genes from A. carolinensis to define the P. platyrhinos macrochromosomes (including the 3a and 3b). If you disagree, please explain to me and other readers why this would not be the case.
6. Metrics for quality of the assembly are needed. BUSCOs were run (in the online data) but the results not reported in the manuscript. Many BUSCOs are missing
C:46.7%[S:46.2%,D:0.5%],F:7.2%,M:46.1%,n:5310
This is much more than I would expect if this is a high quality chromosome-level assembly. Why do you think this is? Was this run on the complete assembly or only the "chromosome" scaffolds? Include a description in the text of this BUSCO analysis and include a summary table of the BUSCO results. If there are other metrics you could use to further understand the quality of this assembly it would be encouraged.
Finer points.
1. Page 13: In methods define the best blast parameters.
2. Synteny Figures: the phylogeny lines are very faint and didn't show up on a print out
3. Table 1: Percent of genome in gaps for Chicago + Hi-C assembly is missing. It is interesting there are MORE gaps in the Chicago + Hi-C Assembly. Why is this?
4. Table 2, it seems this information could easily be incorporated into Figure 2.
5. Table S1 could use a much better description. Is chromosome relative to Anole and Scaffold relative to P. platyrhinos? You have chromosome names rather than scaffold names, this is confusing because the terms are not interchangeable, especially when you are combining and splitting scaffolds to "define" the proposed chromosomes.

6. Figure 3. Why are the chromosomes in some species not sorted by size, when they seem to be in all the other species - is this meaning full in some way?
7. Indicate that you concatenated the 3a and 3b in the synteny figure legends.
8. Describe either in the text or in the readme, the organization of the final assembly. Are the scaffolds organized by size? What are the scaffold names that correspond to each chromosome (this could be included a table or a figure). If the scaffold containing the two microscrosomes is still intact as a single scaffold, report the point at which you think it should be broken. That will be useful information for anyone wanting to use your assembly.
9. Be sure to include a description for all the online files in the Read.me

Methods

Are the methods appropriate to the aims of the study, are they well described, and are necessary controls included? Choose an item.

Conclusions

Are the conclusions adequately supported by the data shown? Choose an item.

Reporting Standards

Does the manuscript adhere to the journal's guidelines on minimum standards of reporting? Choose an item.

Choose an item.

Statistics

Are you able to assess all statistics in the manuscript, including the appropriateness of statistical tests used? Choose an item.

Quality of Written English

Please indicate the quality of language in the manuscript: Choose an item.

Declaration of Competing Interests

Please complete a declaration of competing interests, considering the following questions:

- Have you in the past five years received reimbursements, fees, funding, or salary from an organisation that may in any way gain or lose financially from the publication of this manuscript, either now or in the future?
- Do you hold any stocks or shares in an organisation that may in any way gain or lose financially from the publication of this manuscript, either now or in the future?

- Do you hold or are you currently applying for any patents relating to the content of the manuscript?
- Have you received reimbursements, fees, funding, or salary from an organization that holds or has applied for patents relating to the content of the manuscript?
- Do you have any other financial competing interests?
- Do you have any non-financial competing interests in relation to this paper?

If you can answer no to all of the above, write 'I declare that I have no competing interests' below. If your reply is yes to any, please give details below.

I declare that I have no competing interests

I agree to the open peer review policy of the journal. I understand that my name will be included on my report to the authors and, if the manuscript is accepted for publication, my named report including any attachments I upload will be posted on the website along with the authors' responses. I agree for my report to be made available under an Open Access Creative Commons CC-BY license (http://creativecommons.org/licenses/by/4.0/). I understand that any comments which I do not wish to be included in my named report can be included as confidential comments to the editors, which will not be published.

Choose an item.

To further support our reviewers, we have joined with Publons, where you can gain additional credit to further highlight your hard work (see: https://publons.com/journal/530/gigascience). On publication of this paper, your review will be automatically added to Publons, you can then choose whether or not to claim your Publons credit. I understand this statement.

Yes Choose an item.
